# Mycorrhizal association and life form dominantly control plant litter lignocellulose concentration at the global scale

**DOI:** 10.3389/fpls.2022.926941

**Published:** 2022-07-22

**Authors:** Yan Peng, Ji Yuan, Petr Heděnec, Kai Yue, Xiangyin Ni, Wang Li, Dingyi Wang, Chaoxiang Yuan, Siyi Tan, Fuzhong Wu

**Affiliations:** ^1^Key Laboratory for Humid Subtropical Eco-Geographical Processes of the Ministry of Education, School of Geographical Sciences, Fujian Normal University, Fuzhou, China; ^2^Institute of Tropical Biodiversity and Sustainable Development, University Malaysia Terengganu, Kuala Terengganu, Malaysia; ^3^State Key Laboratory of Remote Sensing Science, Aerospace Information Research Institute, Chinese Academy of Sciences, Beijing, China

**Keywords:** lignin, cellulose, hemicellulose, mycorrhizal association, lifeform, climate, soil properties

## Abstract

Lignocellulose is a major component of plant litter and plays a dominant role in regulating the process of litter decomposition, but we lack a global perspective on plant litter initial lignocellulose concentration. Here, we quantitatively assessed the global patterns and drivers of litter initial concentrations of lignin, cellulose, and hemicellulose using a dataset consisting of 6,021 observations collected from 795 independent publications. We found that (1) globally, the median concentrations of leaf litter lignin, cellulose, and hemicellulose were 20.3, 22.4, and 15.0% of litter mass, respectively; and (2) litter initial concentrations of lignin, cellulose, and hemicellulose were regulated by phylogeny, plant functional type, climate, and soil properties, with mycorrhizal association and lifeform the dominant predictors. These results clearly highlighted the importance of mycorrhizal association and lifeform in controlling litter initial lignocellulose concentration at the global scale, which will help us to better understand and predict the role of lignocellulose in global litter decomposition models.

## Introduction

Litter decomposition is one of the most important processes of carbon (C) and nutrient cycling in terrestrial ecosystems, contributing to the formation of soil organic matter and providing necessary nutrients for plant growth (Berg and McClaugherty, 2020). Litter initial quality is a major factor in controlling plant litter decomposition, and thus affects the availability of soil nutrients, plant nutrient uptake strategies, and nutrient cycling processes across the ecosystem (McClaugherty et al., 1985; Soong et al., 2016). Lignin, cellulose, and hemicellulose, collectively called lignocellulose, are the main abundant components affecting plant litter quality (Sanderson, 2011; Wang et al., 2020). Cellulose and hemicellulose are the main structural components of plant cells and provide vital C sources for fungi (Schwarz, 2001; He et al., 2015), while lignin is one of the most abundant components in plant litter, and can accounts for as much as 30% of the C sequestered in plant litter (Boerjan et al., 2003; He et al., 2021). The dominant role of lignocellulose, especially lignin, in controlling litter decomposition has been widely recognized (Berg and McClaugherty, 2020), and a clear perspective on the global spectrums of plant litter initial concentrations of lignin, cellulose, and hemicellulose will help us to better understand and predict litter decomposition process. However, till now, the global patterns and drivers of plant litter initial lignocellulose concentration still remain elusive.

Lignocellulose is an important source of soil organic C (SOC) and a major component of plant litter (Berg and McClaugherty, 2020). Among the components of lignocellulose, lignin protects cellulose and hemicellulose from enzymatic hydrolysis due to its high resistance to degradation, so cellulose and hemicellulose do not degrade independently of lignin (Kögel-Knabner, 2017; Berg and McClaugherty, 2020). Litter lignocellulose concentration can be affected by a variety of factors, such as phylogeny (e.g., gymnosperm *vs*. angiosperm), plant functional type (PFT, e.g., leaf type, lifeform, and mycorrhizal association), climate, and soil properties (McClaugherty et al., 1985; Aerts, 1997; Cornwell et al., 2008). It is usually acknowledged that litter from tree and shrub species have higher lignocellulose concentration than from herbaceous species, and broadleaved species litter have lower lignocellulose concentration than coniferous species litter (Berg and McClaugherty, 2020). However, it is not clear if litter lignocellulose concentration varied among gymnosperm and angiosperm species. Climate is commonly a determinant factor for plant growth, and it is directly related to plant phenology including the senescence of organs that affect the quantity of litterfall and quality of plant litter (Estiarte and Peñuelas, 2015; Shen et al., 2019). Soil properties can be also important driving factors of litter lignocellulose concentration, among which SOC concentration, moisture, and pH would be the most important ones because they are closely related to the availability of water and essential plant nutrients.

Recently, an increasing number of studies have found that mycorrhizal association is an important factor controlling the absorption and utilization of soil nutrients (Chen et al., 2019; Frey, 2019; Tedersoo and Bahram, 2019), and it may thus also affect the initial concentration of litter lignocellulose. More than 80% of terrestrial vascular plants are associated with mycorrhizas, with arbuscular mycorrhiza (AM) and ectomycorrhiza (ECM) the two dominant types (Tedersoo et al., 2020). AM plants have an advantage in accessing inorganic nutrients, while ECM plants are more capable of mineralizing nutrients directly from organic matter (Liu et al., 2018; Gibert et al., 2019). Also, AM plants prefer moist and warm conditions, but ECM plants prefer dry and cold conditions (Zhong et al., 2021). These differences result in a general pattern of ECM plants being predominant in ecosystems at high latitudes where nutrient cycling is slow, while AM plants are predominant in ecosystems at low latitudes where nutrient cycling is rapid (Zhang et al., 2018). Although studies have found that litter from AM species usually have higher litter quality (Lin et al., 2017), i.e., lower lignocellulose concentration, we still lack a quantitative assessment on the effects of mycorrhizal association, and even know less about the relative importance of mycorrhizal association compared with other factors such as climate and soil properties.

In this study, we quantitatively assessed the initial lignocellulose concentration at the global scale with 6,021 observations collected from 795 peer-reviewed publications. The objectives of this study were to (1) calculate the mean initial concentrations of plant litter lignin, cellulose, and hemicellulose at the global scale; and (2) evaluate the effects and relative importance of multiple driving factors including phylogeny, PFT, climate, and soil properties.

## Materials and methods

### Data collection

**Figure 1:**
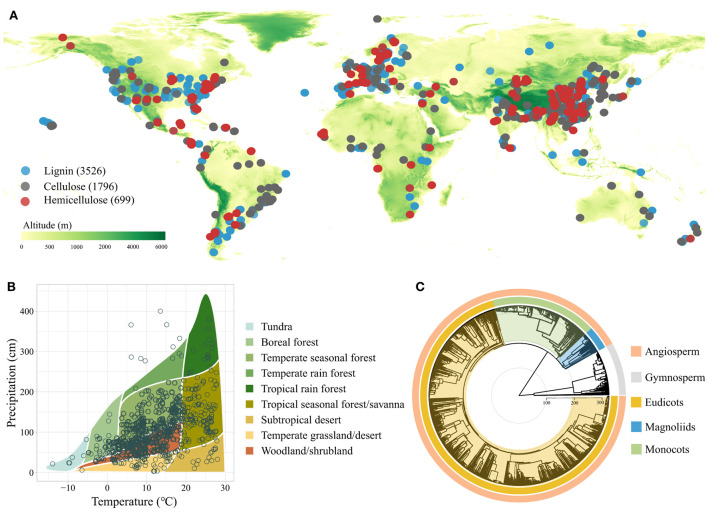
Global map showing the distribution of study sites included in the dataset **(A)**, mean annual temperature and precipitation of study sites by biome **(B)**, and phylogenetic tree of plant species included in the study **(C)**.

Peer-reviewed articles and academic dissertations were searched on *Google Scholar* and *China National Knowledge Infrastructure* (CNKI) at the end of November 2021. The terms used for search were (litter OR leaf OR bark OR branch OR wood) AND (decay OR decomposition OR processing) and their equivalents in Chinese. To collect appropriate data, only primary studies that satisfied the following criteria were included in our database: (1) litter lignocellulose data were obtained through field experiments or observational studies rather than being estimated or remote sensing data; (2) at least one response variable of interest, i.e., initial concentrations of litter lignin, cellulose, or hemicellulose, was reported; and (3) the Latin names and plant litter types (i.e., bark, branch, leaf, root, stem, and wood) associated with litter lignocellulose data were clearly reported. After extraction, a total of 6,021 observations (3,526 for lignin, 1,796 for cellulose, and 699 for hemicellulose) from 795 publications that represented 1,196 species met the criteria (Figure 1, Appendix 1), and were thus included in our database for analyses.

We used a currently published peer-reviewed database named FungalRoot (Soudzilovskaia et al., 2020) to determine the mycorrhizal association of the species included in our database into AM, ECM, and both (i.e., species associated with both AM and ECM fungi) based on the standardized Latin names according to the World Flora Online (www.worldfloraonline.org). Soil property data including SOC concentration, moisture, and pH of the topsoil (0–30 cm) were obtained from the SoilGrids 2.0 database (Poggio et al., 2021) at a 250 m spatial resolution based on the geographic coordinates of the study sites included in our database (Appendix 1). As to climate data, we considered 6 climatic variables, i.e., mean annual temperature (MAT), maximum temperature of the warmest month (TWM), minimum temperature of the coldest month (TCM), mean annual precipitation (MAP), precipitation of the wettest month (PWM), and precipitation of the driest month (PDM), and download these data from the WorldClim v.2 database (Fick and Hijmans, 2017). The aridity index and potential evapotranspiration were obtained from the CGIAR-CSI v.2 database (Trabucco and Zomer, 2019), and both databases provide climate data with a resolution of 1 km^2^. In addition, we determined lifeform following a previous review (Richardson and Rejmánek, 2011), and classified phylogeny and leaf type according to online botanical databases of *Missouri Botanical Garden* (http://www.missouribotanicalgarden.org), *eFloras* (http://www.efloras.org), and *Identification guide for the wild trees of the Canary Archipelago* (https://www.arbolappcanarias.es) in case that such information was not directly reported in the primary studies.

### Data analyses

To assess the effects of litter type, phylogeny, lifeform, leaf type, mycorrhizal association, climate, and soil properties on litter lignocellulose concentrations, we first ran linear mixed-effects models with the *lme4* package (Bates et al., 2015) by fitting each predictor variable as a fixed-effect factor and the identity of primary studies from which data were collected as a random effect factor, which explicitly accounted for the potential dependence of data points collected from a single primary study. To further evaluate the relative importance of variables that showed significant effects on litter lignocellulose concentration, we used linear mixed-effects model selection approach with the *glmulti* package (Calcagno and de Mazancourt, 2010). A cutoff of 0.8 for the Akaike weights was set to differentiate the essential and non-essential predictor variables (Yue et al., 2021). Because of the limited data points for bark, branch, root, stem, and wood litter, analyses for assessing the effects of driving factors were only conducted for leaf litter. All statistical analyses were performed using R version 4.1.2 (R Core Team, 2021).

## Results

### Patterns of litter lignocellulose concentration

**Figure 2:**
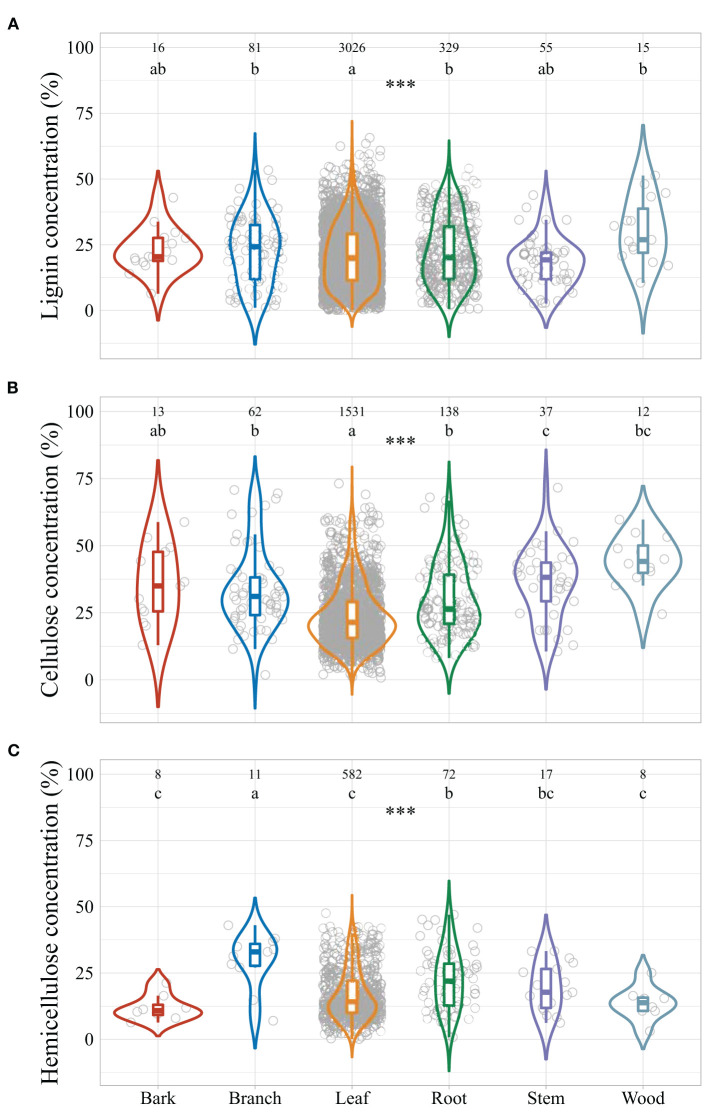
Violin plots of the concentrations of plant litter lignin **(A)**, cellulose **(B)**, and hemicellulose **(C)** grouped by litter types. Asterisks indicate significant effects of litter type, and different letters indicate significant differences among different litter types at α = 0.05. ***p < 0.001.

Averaged across all the observations, global leaf litter lignin concentration ranged from 0.2 to 65.7% of litter mass (median: 20.3%), cellulose concentrations from 0.8 to 73.2% (median: 22.4%), and hemicellulose concentrations from 0.1 to 47.6% (median: 15.0%) (Figure 2). Litter lignocellulose concentrations varied among litter types, with highest lignin concentrations in branch, stem, and root litter but lowest in leaf litter. Cellulose concentrations in branch and root litter were similar to those in bark and wood litter, but higher than in leaf litter and lower than in stem litter. Root litter hemicellulose concentration was similar to stem litter, but was lower than bark, leaf, and wood litter and higher than branch litter.

**Figure 3:**
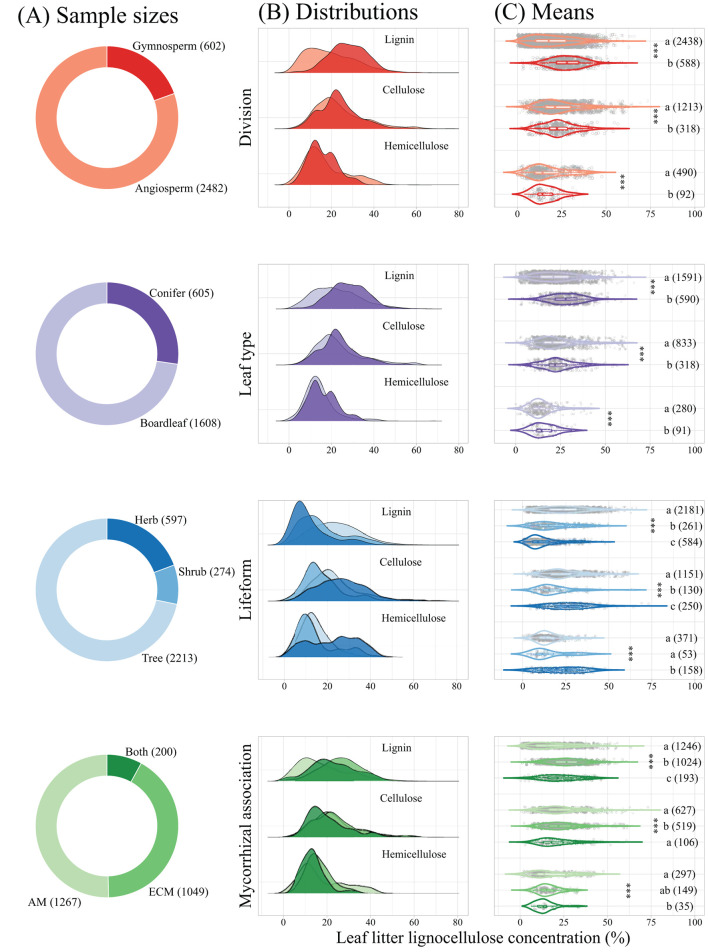
Sample size, distribution, and leaf litter lignocellulose concentration estimates across taxonomic division, leaf type, lifeform, and mycorrhizal association. Panel **(A)** presents sample sizes, Panel **(B)** presents kernel density estimates fit to subsets of the dataset (based on the sample sizes), and Panel **(C)** represents violin plots of leaf litter lignocellulose concentrations. Asterisks indicate significant effects of taxonomic division, leaf type, lifeform, or mycorrhizal association, and different letters indicate significant differences at α = 0.05. ***p < 0.001. AM, arbuscular mycorrhiza; ECM, ectomycorrhiza; Both, plants associated with both AM and ECM fungi.

Taxonomic division, leaf type, lifeform, and mycorrhizal association significantly affected leaf litter lignocellulose concentration (Figure 3). The concentrations of leaf litter lignin and cellulose from gymnosperm species were significantly higher than from angiosperm species, while leaf litter hemicellulose concentration showed an opposite trend. Leaf litter lignin, cellulose, and hemicellulose concentrations were all significantly higher in coniferous species than in broadleaved species, and varied significantly among trees, shrubs, and herbs, with higher cellulose and hemicellulose but lower lignin concentrations in herbs compared with trees. Mycorrhizal association significantly affected leaf litter lignocellulose concentration, with lower hemicellulose concentration of AM plants comparted with ECM plants and plants associated with both AM and ECM fungi. However, leaf litter lignin and cellulose concentrations of ECM plants was significantly higher than that of AM plants and plants associated both AM and ECM fungi.

### Factors explaining variations in leaf litter lignocellulose concentration

**Figure 4:**
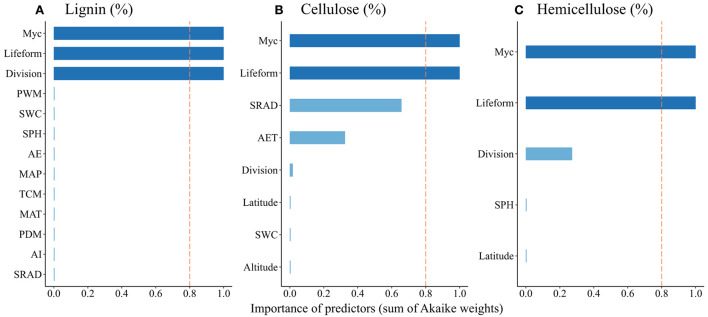
Model-averaged importance of multiple predictors of litter initial concentrations of lignin **(A)**, cellulose **(B)**, and cellulose **(C)**. Cutoff (red dashed line) is set at 0.8 to explore the essential (deep blue) and non-essential (light blue) predictors. Myc, mycorrhizal association; PWM, precipitation of the wettest month; SWC, soil water content; SPH, soil pH; AET, annual evapotranspiration; MAP, mean annual precipitation; TCM, minimum temperature of the coldest month; MAT, mean annual temperature; PDM, precipitation of the driest month; ADI, aridity index; DSR, daily mean solar radiation.

Leaf litter lignin concentration was negatively affected by annual evapotranspiration (AET), daily mean solar radiation (DSR), and soil pH, but positively affected by MAT, TCM, MAP, PWM, PDM, aridity index (ADI), and soil moisture (Table 1). Leaf litter cellulose concentration was positively affected by AET, DSR, soil moisture and latitude, but negatively influenced by altitude. Leaf litter hemicellulose concentration was positively related to latitude and soil pH. As to the relative importance of the factors that showed significant effects, mycorrhizal association, lifeform, and taxonomic division were the essential predictors of leaf litter lignin concentration, while leaf litter cellulose and hemicellulose concentrations were best predicted by mycorrhizal association and lifeform (Figure 4).

**Table 1:** Effects of climate and soil properties on the initial concentrations of leaf litter lignin, cellulose, and hemicellulose as assessed by linear mixed-effects models.

**Predictor**	**Lignin**			**Cellulose**			**Hemicellulose**		
	**Estimate**	***p***	***n***	**Estimate**	***p***	***n***	**Estimate**	***p***	***n***
MAT	0.106	**0.014**	3024	0.065	0.271	1529	−0.011	0.897	580
TWM	0.013	0.834	3024	0.120	0.126	1529	0.164	0.166	580
TCM	0.087	**0.001**	3024	0.033	0.383	1529	−0.034	0.514	580
MAP	0.003	**<0.001**	3024	0.000	0.975	1529	−0.001	0.141	580
PWM	0.015	**<0.001**	3024	0.006	0.188	1529	−0.004	0.445	580
PDM	0.024	**0.021**	3024	0.008	0.625	1529	−0.029	0.201	580
ADI	0.000	**<0.001**	3024	−0.000	0.174	1529	−0.000	0.052	580
AET	−0.002	**0.006**	3024	0.002	**0.046**	1529	0.000	0.754	580
DSR	−0.000	**0.018**	3024	0.000	**0.032**	1529	0.000	0.835	580
SOC	−0.005	0.071	3021	−0.004	0.368	1526	0.004	0.441	582
pH	−1.697	**<0.001**	3021	0.340	0.513	1526	2.593	**<0.001**	582
Moisture	0.136	**<0.001**	3021	0.141	**0.009**	1526	0.066	0.326	582
Altitude	−0.000	0.718	3024	−0.001	**0.024**	1529	−0.001	0.282	580
Latitude	0.005	0.732	3026	0.062	**0.018**	1531	0.083	**0.008**	580

Estimate (i.e., slope of the model), p-value, and number of observations were reported. Bold indicate statistical significant effects.

MAT, mean annual temperature; TWM, maximum temperature of the warmest month; TCM, minimum temperature of the coldest month; MAP, mean annual precipitation; PWM, precipitation of the wettest month; PDM, precipitation of the driest month; ADI, aridity index; AET, annual evapotranspiration; DSR, daily mean solar radiation; SOC, soil organic carbon; pH, soil pH; moisture, soil moisture.

## Discussion

Our results showed that litter lignin concentration was higher in woody plants (i.e., trees and shrubs) than in herbaceous plants, but cellulose and hemicellulose concentrations showed a opposite trend, which may be mainly attributable to the presence of ligneous tissue in woody plant litter (Lorenz and Lal, 2005). Both litter cellulose and hemicellulose concentrations were higher in herbaceous plants than in woody plants, which was consistent with the results of previous studies (Ma et al., 2018). In general, woody plants have high lignin concentration because they typically require a proportional increase in C investment at the cellular level to synthesize lignin to support structures with relatively low growth rates. In contrast, the relatively high growth rates of herbaceous plants are associated with the low lignin and C concentrations (Ma et al., 2018).

Our results suggested that litter lignin and cellulose concentrations were higher in gymnosperms and conifers than in angiosperms and broadleaved trees, respectively, which may be partly attributed to their differences in functional traits such as leaf structure, photosynthetic capacity, and litter chemistry (Augusto et al., 2015). Conifers are generally characterized by lower litter quality (e.g., high lignin/N ratio) than broadleaved trees, and are generally recognized as undesirable substrates for decomposer communities (Binkley and Giardina, 1998; Prescott et al., 2004; Hobbie et al., 2006). Compared to conifers, broadleaved trees have a higher nutrient uptake capacity and higher litter quality (Wright et al., 2004; Freschet et al., 2012). Litter hemicellulose concentration was greater in angiosperms than in gymnosperms, while those in broadleaved trees were lower than those in conifers. Two plausible mechanisms may explain this result: (1) the unbalanced data points between the two levels of taxonomic division (490 for angiosperms and 92 for gymnosperms) or leaf type (280 for broadleaved trees and 91 conifers) may resulted in biased results; and (2) hemicellulose is present in all living plant tissues because it is an essential compound for primary and secondary plant cell walls (Schädel et al., 2010). Our findings that herbaceous plants and angiosperms had higher litter hemicellulose concentration than woody plants and gymnosperms, respectively, were in consistent with previous research (Hoch, 2007).

Recently, mycorrhizal association been has recognized as an important driver of ecosystem functions (Phillips et al., 2013; Peng et al., 2022). Our results from quantitative analyses at the global scale showed that litter lignin concentration of ECM plants were higher than AM plants, which was in accord with previous results (Lin et al., 2017; Sun et al., 2018. AM plants generally produce degradable litter with low lignin and cellulose concentrations, while slowly decomposing litter from ECM plants usually has higher lignin and cellulose concentrations (Ma et al., 2018). Also, the different litter decomposition rates of AM and ECM plants lead to distinctness in soil microbial community activities and soil properties (Brzostek et al., 2015; Cheeke et al., 2017), and in turn regulate litter quality including lignocellulose concentration. For example, although AM fungi do not directly participate in litter decomposition processes, they can indirectly influence litter decomposition through changing soil microbial activity and thus soil nutrient profiles (Paterson et al., 2016; Bunn et al., 2019). While ECM fungi can hydrolyze plant litter to obtain nutrients for growth and metabolic functions (Bödeker et al., 2016; Cheeke et al., 2017). Therefore, mycorrhizal association can affect litter lignin and cellulose concentrations by influencing soil nutrient profiles and plant nutrient uptake strategies (Cornelissen et al., 1999). In addition, we found that MAT, TCM, MAP, PWM, PDM, ADI, AET, and DSR had significant effects on litter lignin concentration, which may be attributed to that these factors are directly related to plant growth and nutrient uptake strategies. However, our findings showed no effect of climate factors on litter cellulose or hemicellulose concentration, indicating the independence of litter cellulose and hemicellulose concentration on climate.

In summary, our quantitative assessment on global plant litter lignocellulose concentration showed that the median of leaf litter lignin, cellulose, and hemicellulose concentrations were 20.3, 22.4, and 15.0%, respectively. Litter lignin concentration was affected by phylogeny, leaf type, lifeform, mycorrhizal association, climate, and soil properties, while litter cellulose and hemicellulose concentrations were affected by phylogeny, leaf type, lifeform, mycorrhizal association, and soil properties. Mycorrhizal association, lifeform, and phylogeny were the most important factors controlling litter initial lignocellulose concentration. Overall, our results clearly showed the global spectrums and underlying driving factors of the initial concentrations of litter lignin, cellulose, and hemicellulose, which highlighted the importance of mycorrhizal association and lifeform in controlling litter initial lignocellulose concentration. Our results will help us to better understand the role of lignocellulose in litter decomposition process and the related biogeochemical cycles.

## Data availability statement

Raw data used in this study were deposited in figshare with a DOI (https://doi.org/10.6084/m9.figshare.20210534.v1).

## Author contributions

YP, JY, KY, and FW conceived the study. JY, XN, DW, CY, and ST collected raw data. YP, JY, PH, WL, and KY performed statistical analyses and figure drawing. JY, YP, and FW wrote the first draft of the manuscript with contributions from all coauthors. YP and FW thoroughly revised the manuscript according to the comments from reviewers, with suggestions from all coauthors. All authors contributed to the article and approved the submitted version.

## Funding

This study was supported by the National Natural Science Foundation of China (31922052, 32011530426, 31800373, 32171641, 32022056, and 31800521) and the Youth Innovation Promotion Association Chinese Academy of Sciences (Grant 2018084).

## Conflict of interest

The authors declare that the research was conducted in the absence of any commercial or financial relationships that could be construed as a potential conflict of interest.

## Publisher's note

All claims expressed in this article are solely those of the authors and do not necessarily represent those of their affiliated organizations, or those of the publisher, the editors and the reviewers. Any product that may be evaluated in this article, or claim that may be made by its manufacturer, is not guaranteed or endorsed by the publisher.
