# Supplementary material for: Mycorrhizal association and life form dominantly control plant litter lignocellulose concentration at the global scale
Source: Front Plant Sci. 2022 Jul 22;13:926941. doi: 10.3389/fpls.2022.926941 (PMC9355614; doi:10.3389/fpls.2022.926941)
Supplement: Supplementary file 1 [file Data_Sheet_1.docx]

**Appendix 1.** A list of the primary studies from with raw data used in the meta-analysis were extracted.

1 Y. Xiang, H. Hu, T. Hu, J. Wan, M. Pu, Z. Yan, Y. Ding. (2011). Study on the characteristics of litter production and decomposition in plantations of Eucalyptus grandis in rainy area of west China. Journal of Sichuan Agricultural University. 29(4), 465-471. (in Chinese with English abstract)

2 Y. Liu, W. Yang, F. Wu, Z. Xu, F. Yang, B. Li, Y. Chang, B. Tan. (2017). Soil organic layer enzyme activities in subalpine coniferous forests of Western Sichuan, China. Ecology and Environmental Sciences. 26(5), 747-753. (in Chinese with English abstract)

3 Y. Qin, J. Ma, J. Mei, D. Yang, F. Zhuang, J. Su. (2017). The initial dynamic of litter decomposition of Loropetalum chinense communities amomg different recovery stages in karst area of Lijiang river watershed. Acta Ecologica Sinica. 37(20), 6792-6799. (in Chinese with English abstract)

4 H. Wang, P. Yan, P. Zhan, X. Zhang, Z. Liu, G. Yujing, D. Xiao. (2018). The relative contributions of plant quality，simulated rising temperature，and habitat to litter decomposition. Chinese Journal of Applied Ecology. 29(2), 474-482. (in Chinese with English abstract)

5 Y. Li, T. Xie, W. Shi, X. Li. (2019). Response of topsoil organic carbon mineralization to litter addition in the revegetation area in the southeastern fringe of the Tengger Desert. Journal of Desert Research. 39(5), 200-209. (in Chinese with English abstract)

6 H. Yang, Y. Li, Z. Ning, Z. Zhang. (2019). Effects of mixed litter on organic carbon mineralization in a dune grassland. Acta Ecologica Sinica. 39(7), 2510-2519. (in Chinese with English abstract)

7 X. Zhang, L. Zhang, H. Lei, S. Wang, Y. Dong, H. Mi, Z. Liu. (2020). Effects of combined remediation using grass litters and urea on the biochemical properties of petroleum-contaminated soil. Acta Ecologica Sinica. 40(8), 2715-2725. (in Chinese with English abstract)

8 A. Tian, J. Wang, Z. Han, J. Wu, W. Li. (2020). Impacts on decomposition of flower to leaf ration in the litter of rhododendron delavayi in baili azalea forest area of Guizhou Province. Scientia Silvae Sinicae. 56(8), 1-10. (in Chinese with English abstract)

9 W. Li. (2016). Effect of nutrient addition on litter decomposition in Stipa baicalensis steppe. Shenyang Agricultural University. (in Chinese with English abstract)

10 S. Li. (2015). Effects of substrata on riparian plant litter decomposition in a mountain stream, SW China. Central China Normal University. (in Chinese with English abstract)

11 C. Long. (2020). Litter decomposition processes and their microbiological characteristics under different land use types. Wuhan Botanical Garden, Chinese Academy of Sciences. (in Chinese with English abstract)

12 Z. Fu. (2019). Response characteristics of litter decomposition with different masses to altitude gradient. Fujian Normal University. (in Chinese with English abstract)

13 Y. Lv. (2013). Effect of nitrogen and acid deposition on litter decomposition in subtropocal forest ecosystem. Nanjing University. (in Chinese with English abstract)

14 K. Li. (2006). Nitrogen deposition affects carbon and nitrogen turnover on the early stage of forest litter decomposition. Northeast Forest University. (in Chinese with English abstract)

15 Y. Bai. (2020). Effects of Nitrogen deposition on litter decomposition of different communities in Changbai Montain tundra. Northeast Normal University. (in Chinese with English abstract)

16 S. Peng. (2016). Effects of nitrogen addition on litter decomposition in poplar plantations in a coastal area, China. Nanjing Forestry University. (in Chinese with English abstract)

17 J. Li. (2016). Interactions between invertebrate and microbial communities in decomposing litter along with the change of altitude. Sichuan Agricultural University. (in Chinese with English abstract)

18 R. Lv. (2010). The effect of thinning on litter decomposition of the coniferous plantation. Beijing Forestry University. (in Chinese with English abstract)

19 M. Pang. (2018). Relationship between litter decomposition and litter traits in subtropical evergreen broad-leaved forest in Gutianshan, China. Chongqing University. (in Chinese with English abstract)

20 J. Wu. (2015). The effect of light radiation on litter decomposition in an alpine meadow of the Tibetan Plateau. Lanzhou University. (in Chinese with English abstract)

21 R. Deng. (2008). Effect of Seasonal freeze-thaw on litter decomposition in the subalpine forest. Sichuan Agricultural University. (in Chinese with English abstract)

22 P. Liu. (2006). Characteristics and responses to changes in environmental conditions of litter decomposition in plant species of Stipa krylovii Roshev. steppe. Institute of Botany, the Chinese Academy of Sciences. (in Chinese with English abstract)

23 Z. Ma. (2015). Effect of simulating light intensity on forest litter decomposition. Sichuan Agricultural University. (in Chinese with English abstract)

24 H. Gao. (2019). Decomposition characteristics of common plant litters in the stipa breviflora desert grassland of Inner. Inner Mongolia Agricultural University. (in Chinese with English abstract)

25 L. Zhang. (2017). Study on decomposition of 15 common plant litter in Tibetan alpine meadow. Lanzhou University. (in Chinese with English abstract)

26 F. Wang. (2013). Effect of species richness and evenness on litter decomposition and N and P release in alpine meadow of the Tibetan Plateau. Lanzhou University. (in Chinese with English abstract)

27 Q. Yu. (2015). Effect of different micro-sites and soil fauna on litter decomposition in an alpine swamp meadow of the Tibetan Plateau. Lanzhou University. (in Chinese with English abstract)

28 J. Gu. (2016). Research on aquatic plant litter decomposition characteristics and mechanism of constructed wetlands clogging. Nanjing University of Information Science and Technology. (in Chinese with English abstract)

29 W. Wang. (2014). Contributions of soil fauna to litter decomposition in ropical evergreen broad-leaved forests in Sichuan basin. Sichuan Agricultural University. (in Chinese with English abstract)

30 S. Gong. (2015). Response of litter decomposition and soil biological activity to increased temperature and nitrogen deposition in the Songnen grassland. Northeast Normal University. (in Chinese with English abstract)

31 B. Song. (2008). Study on characteristics of soil fauna community and their function in litter decomposition in Leymus chinensis steppe, Songnen plain. Northeast Normal University. (in Chinese with English abstract)

32 Z. Wang. (2017). The analysis of soil microbe diversity during the decomposition of mixed litter under Cunninghamia lanceolata plantations in acid rain region. Fujian Agriculture & Forestry University. (in Chinese with English abstract)

33 L. Chen. (2014). The initial responses of simulated N deposition in litter decomposition of intercropping system Alnus formosana- Lolium multiflorum compound mode. Sichuan Agricultural University. (in Chinese with English abstract)

34 Y. Liu. (2010). Litter decomposition and nutrient dynamics on soil in complex ecosystem of Alnus formosana-Hemarthria compressa. Sichuan Agricultural University. (in Chinese with English abstract)

35 J. Fang. (2020). Effects of carbon, nitrogen addition and dry-wet alternations on litter decomposition of Robinia pseudoacacia forest in dump site. Liaoning Technical University. (in Chinese with English abstract)

36 X. Dong. (2018). Litter decomposition mechanism driven by temperature and nitrogen deposition. Heilongjiang University. (in Chinese with English abstract)

37 Y. Lu. (2020). Analysis of mixed effects of leaf litter decomposition and its main affecting factors of dominant tree species in Yaoxiang Forest Farm. Shandong Agricultural University. (in Chinese with English abstract)

38 Q. Meng. (2008). Effects of warming and nitrogen deposition on Leymus chinensis and Phragmites communis litter decomposition. Northeast Normal University. (in Chinese with English abstract)

39 Y. Jiang. (2013). Litter decomposition and functional role of soil fauna in decomposition in a Pinus koraiensis mixed broad-leaved forest of Changbai Mountains. Northeast Normal University. (in Chinese with English abstract)

40 Z. Wang. (2016). Litter decomposition and the effects of soil fauna on the litter decomposition and nutrient release in the coniferous forest of the Changbai Mountains, China. Northeast Normal University. (in Chinese with English abstract)

41 X. Shi. (2009). Study on decomposition of soil organic carbon and soil litters in typical regions of the eastern China. Nanjing Agricultural University. (in Chinese with English abstract)

42 H. Lin. (2017). The effects of soil fauna on litter decomposition and its driving mechanism in Zijin Mountains. Nanjing University. (in Chinese with English abstract)

43 Q. Liu, S. Peng, H. Bi, H. Zhang, Z. Li, W. Ma, N. Li. (2005). Nutrient dynamics of foliar litter in reciprocal decomposition in tropical and subtropical forests. Journal of Beijing Forestry University. 27(1), 24-32. (in Chinese with English abstract)

44 X. Li, S. Han, Z. Guo, X. Zheng, G. Song, K. Li. (2006). Decomposition of pine needles and twigs on and under the litter layer in the natural Korean pine broadleaved forests. Journal of Beijing Forestry University. 28(3), 8-13. (in Chinese with English abstract)

45 X. Deng, Y. Zhang, S. Han, X. Li, K. Li, X. Li. (2007). Effects of nitrogen addition on the early stage decomposition of Korean pine litter in the Changbaishan Mountains, northeastern China. Journal of Beijing Forestry University. 29(6), 16-22. (in Chinese with English abstract)

46 X. Li, S. Han, Y. Zhang. (2007). Indirect effects of precipitation on litter decomposition of Quercus mongolica. Chinese Journal of Applied Ecology. 18(2), 261-266. (in Chinese with English abstract)

47 Y. Xu. (2007). The effects of Thinning on litter decomposition of Pinus tabulaeformis plantation. Beijing Forestry University. (in Chinese with English abstract)

48 F. He, D. Wang, R. Lei, Y. Wang, G. Yu. (2008). Nutrient properties of litter leaves of major tree species at Qinling Mountains. Journal of Northwest Forestry University. 23(4), 30-33. (in Chinese with English abstract)

49 G. Lei, Y. Liu, R. Li, Y. Xu, B. Guo. (2008). Responses of decomposition rate, nutrient return and composition of leaf litter to thinning intensities in Pinus tabulaeformis plantation. Journal of Beijing Forestry University. 30(5), 52-57. (in Chinese with English abstract)

50 Y. Chang. (2009). Litter decomposition and its respnses to N deposition for coniferous forests of Qinling Western mountains. Lanzhou University. (in Chinese with English abstract)

51 Y. Ma, H. Jiang, S. Yu, D. Rongpeng, P. Guo, B. Wang. (2009). Leaf litter decomposition of plants with different origin time in the mid-subtropical China. Acta Ecologica Sinica. 29(10), 5237-5245. (in Chinese with English abstract)

52 X. Song, H. Song, S. Yu, Y. Ma, G. Zhou, R. Dou, P. Guo. (2009). Litter decomposition of dominant plant species in successional stages in mid-subtropical zone. Chinese Journal of Applied Ecology. 20(3), 537-542. (in Chinese with English abstract)

53 R. Dou, H. Jiang, S. Yu, Y. Ma, P. Guo, X. Song. (2010). Leaf litter decomposition of six trees in Mid-subtropical and tropical China. Acta Ecologica Sinica. 30(16), 4521-4528. (in Chinese with English abstract)

54 R. Dou, H. Jiang, S. Yu, Y. Ma, X. Song, P. Guo, X. Zhang. (2010). Leaf litter decomposition of four tree species in subtropical China. Journal of Zhejiang Forestry College. 27(2), 163-169. (in Chinese with English abstract)

55 H. Zhang. (2010). The effect of UV-B radiation on decomposition of leaf litter of main tree species in subtropical region. Zhejiang A&F University. (in Chinese with English abstract)

56 F. Chen, H. Zheng, B. Yang, Z. Ouyang, K. Zhang, Y. ZXiao, N. Tu. (2011). The decomposition of coniferous and broadleaf mixed litters significantly changes the carbon metabolism diversity of soil microbial communities in subtropical area，southern China. Acta Ecologica Sinica. 31(11), 3027-3035. (in Chinese with English abstract)

57 X. Guo. (2013). Comparative studies on potential carbon sequestration of lakeshore plants in plateau wetland, northwestern Yunnan. Southwest Forestry University. (in Chinese with English abstract)

58 G. Huang, L. Zhou, L. Tang, Y. Li, H. Zhao. (2013). Photodegradation of plant litter in a temperate desert along a precipitation gradient. Chinese Journal of Ecology. 32(10), 2574-2582. (in Chinese with English abstract)

59 R. Lyu, G. Li, Y. Liu, H. Jin, N. Lin. (2013). Study on initial decomposition characteristics of Pinus tabulaeformis needle litter at different stand age. Journal of Nanjing Forestry University ( Natural Sciences Edition). 37(2), 39-44. (in Chinese with English abstract)

60 y. Xiao, L. Tu, T. Hu, B. Chen, Y. Yang, J. Zhang, X. Li, H. Hu. (2013). Effects of simulated nitrogen deposition on substrate quality of litterfall in a Pleioblastus amarus plantation in Rainy Area of West China. Acta Ecologica Sinica. 33(20), 6587-6594. (in Chinese with English abstract)

61 q. Wu, F. Wu, W. Yang, Y. Zhao, W. He, M. He, J. Zhu. (2015). Effect of snow cover on phosphorus release from leaf litter in the alpine forest in eastern Qinghai-Tibet plateau. Acta Ecologica Sinica. 35(12), 4115-4127. (in Chinese with English abstract)

62 Y. Chen, Y. Liu, C. Fan, X. Li, Y. Liu, Z. Yang, J. Zhang. (2015). Litter decomposition and nutrient release dynamics in complex ecosystem of Alnus formosana-Hemarthria compressa. Journal of Nanjing Forestry University ( Natural Sciences Edition). 39(1), 49-54. (in Chinese with English abstract)

63 Y. Chen, R. He, Y. Liu, J. Zhang, C. Deng, X. Song, L. Yang, J. Liu. (2016). Leaf litter invertase activity in the early stage of litter decomposition in alpine timberline ecotone of western Sichuan, China. Acta Ecologica Sinica. 36(13), 4099-4108. (in Chinese with English abstract)

64 Y. Chen, F. Wang, Q. Mo, Q. Li, B. Zou, Y. Li, X. Li, J. Wu, Z. Li. (2015). Effects of nitrogen and phosphorus additions on woody debris decomposition ffects of nitrogen and phosphorus additions on woody debris decomposition in a secondary tropical forest of South China. Chinese Journal of Applied and Environmental Biology. 21(4), 747-753. (in Chinese with English abstract)

65 Y. Li, Q. Li, J. Yang, X. Lv, W. Liang, X. Han. (2016). Effect of simulated nitrogen deposition on litter quality in a temperate grassland. Chinese Journal of Ecology. 35(10), 2732-2737. (in Chinese with English abstract)

66 J. Li, Y. Zhang, X. Li, Y. Han. (2017). Impact of precipitation intensity on the decomposition of floor litter and the fine roots of Picea schrenkiana. Bulletin of Botanical Research. 37(3), 360-369. (in Chinese with English abstract)

67 S. Liao. (2017). Soil fauna effects on chemical compounds in foliar litters during decomposition in seven ecosystems along an altitudinal gradient in the Minjiang River basin. Sichuan Agricultural University. (in Chinese with English abstract)

68 K. L. Murphy, J. M. Klopatek, C. C. Klopatek. (1998). The effects of litter quality and climate on decomposition along an elevational gradient. Ecological Applications. 8(4), 1061-1071.

69 C. Smith, H. Gholz, F. de Assis Oliveira. (1998). Fine litter chemistry, early-stage decay, and nitrogen dynamics under plantations and primary forest in lowland Amazonia. Soil Biology and Biochemistry. 30(14), 2159-2169.

70 J. Anderson, S. Hetherington. (1999). Temperature, nitrogen availability and mixture effects on the decomposition of heather [Calluna vulgaris (L.) Hull] and bracken [Pteridium aquilinum (L.) Kuhn] litters. Functional Ecology. 13, 116-124.

71 G. Buamscha, M. Gobbi, M. Mazzarino, F. Laos. (1998). Indicators of nitrogen conservation in Austrocedrus chilensis forests along a moisture gradient in Argentina. Forest Ecology and Management. 112(3), 253-261.

72 M. Carreiro, K. Howe, D. Parkhurst, R. Pouyat. (1999). Variation in quality and decomposability of red oak leaf litter along an urban-rural gradient. Biology and Fertility of Soils. 30(3), 258-268.

73 M. F. Cotrufo, A. Raschi, M. Lanini, P. Ineson. (1999). Decomposition and nutrient dynamics of Quercus pubescens leaf litter in a naturally enriched CO2 Mediterranean ecosystem. Functional Ecology. 13(3), 343-351.

74 L. C. Duchesne, S. Wetzel. (1999). Effect of clear-cutting, prescribed burning and scarification on litter decomposition in an eastern Ontario jack pine (Pinus banksiana) ecosystem. International Journal of Wildland Fire. 9(3), 195-201.

75 D. Gillon, R. Joffre, A. Ibrahima. (1999). Can litter decomposability be predicted by near infrared reflectance spectroscopy? Ecology. 80(1), 175-186.

76 T. Henriksen, T. Breland. (1999). Decomposition of crop residues in the field: evaluation of a simulation model developed from microcosm studies. Soil Biology and Biochemistry. 31(10), 1423-1434.

77 T. M. Henriksen, T. A. Breland. (1999). Evaluation of criteria for describing crop residue degradability in a model of carbon and nitrogen turnover in soil. Soil Biology and Biochemistry. 31(8), 1135-1149.

78 V. Jamaludheen, B. M. Kumar. (1999). Litter of multipurpose trees in Kerala, India: variations in the amount, quality, decay rates and release of nutrients. Forest Ecology and Management. 115(1), 1-11.

79 N. Kaneko, E. Salamanca. (1999). Mixed leaf litter effects on decomposition rates and soil microarthropod communities in an oak–pine stand in Japan. Ecological Research. 14(2), 131-138.

80 J. Kanmegne, B. Duguma, J. Henrot, N. Isirimah. (1999). Soil fertility enhancement by planted tree-fallow species in the humid lowlands of Cameroon. Agroforestry Systems. 46(3), 239-249.

81 A. Kwabiah, R. Voroney, C. Palm, N. Stoskopf. (1999). Inorganic fertilizer enrichment of soil: effect on decomposition of plant litter under subhumid tropical conditions. Biology and Fertility of Soils. 30(3), 224-231.

82 C. Robinson, J. Kirkham, R. Littlewood. (1999). Decomposition of root mixtures from high arctic plants: a microcosm study. Soil Biology and Biochemistry. 31(8), 1101-1108.

83 G. Seneviratne, L. Van Holm, L. J. Balachandra, S. Kulasooriya. (1999). Differential effects of soil properties on leaf nitrogen release. Biology and fertility of Soils. 28(3), 238-243.

84 K. Singh, P. Singh, S. Tripathi. (1999). Litterfall, litter decomposition and nutrient release patterns in four native tree species raised on coal mine spoil at Singrauli, India. Biology and Fertility of Soils. 29(4), 371-378.

85 M. Wedderburn, J. Carter. (1999). Litter decomposition by four functional tree types for use in silvopastoral systems. Soil Biology and Biochemistry. 31(3), 455-461.

86 M. Diack, M. Sene, A. Badiane, M. Diatta, R. Dick. (2000). Decomposition of a native shrub, Piliostigma reticulatum, litter in soils of semiarid Senegal. Arid soil research and rehabilitation. 14(3), 205-218.

87 S. Frey, E. Elliott, K. Paustian, G. Peterson. (2000). Fungal translocation as a mechanism for soil nitrogen inputs to surface residue decomposition in a no-tillage agroecosystem. Soil Biology and Biochemistry. 32(5), 689-698.

88 H. L. Gholz, D. A. Wedin, S. M. Smitherman, M. E. Harmon, W. J. Parton. (2000). Long‐term dynamics of pine and hardwood litter in contrasting environments: toward a global model of decomposition. Global Change Biology. 6(7), 751-765.

89 S. E. Hobbie, P. M. Vitousek. (2000). Nutrient limitation of decomposition in Hawaiian forests. Ecology. 81(7), 1867-1877.

90 L. Isaac, C. W. Wood, D. A. Shannon. (2000). Decomposition and nitrogen release of prunings from hedgerow species assessed for alley cropping in Haiti. Agronomy Journal. 92(3), 501-511.

91 W. Liu, J. E. Fox, Z. Xu. (2000). Leaf litter decomposition of canopy trees, bamboo and moss in a montane moist evergreen broad-leaved forest on Ailao Mountain, Yunnan, south-west China. Ecological Research. 15(4), 435-447.

92 B. Longdoz, M. Yernaux, M. Aubinet. (2000). Soil CO2 efflux measurements in a mixed forest: impact of chamber disturbances, spatial variability and seasonal evolution. Global Change Biology. 6(8), 907-917.

93 A. H. Magill, J. D. Aber. (2000). Dissolved organic carbon and nitrogen relationships in forest litter as affected by nitrogen deposition. Soil Biology and Biochemistry. 32(5), 603-613.

94 M. W. Paschke, T. McLendon, E. F. Redente. (2000). Nitrogen availability and old-field succession in a shortgrass steppe. Ecosystems. 3(2), 144-158.

95 H. Chen, M. E. Harmon, J. Sexton, B. Fasth. (2002). Fine-root decomposition and N dynamics in coniferous forests of the Pacific Northwest, USA. Canadian Journal of Forest Research. 32(2), 320-331.

96 J. Cobo, E. Barrios, D. C. L. Kass, R. J. Thomas. (2002). Decomposition and nutrient release by green manures in a tropical hillside agroecosystem. Plant and Soil. 240(2), 331-342.

97 A. V. De Santo, F. A. Rutigliano, B. Berg, A. Fioretto, G. Puppi, A. Alfani. (2002). Fungal mycelium and decomposition of needle litter in three contrasting coniferous forests. Acta Oecologica. 23(4), 247-259.

98 B. John, H. Pandey, R. Tripathi. (2002). Decomposition of fine roots of Pinus kesiya and turnover of organic matter, N and P of coarse and fine pine roots and herbaceous roots and rhizomes in subtropical pine forest stands of different ages. Biology and Fertility of Soils. 35(4), 238-246.

99 G. Loranger, J.-F. Ponge, D. Imbert, P. Lavelle. (2002). Leaf decomposition in two semi-evergreen tropical forests: influence of litter quality. Biology and fertility of soils. 35(4), 247-252.

100 C. Mathuriau, E. Chauvet. (2002). Breakdown of leaf litter in a neotropical stream. Journal of the North American Benthological Society. 21(3), 384-396.

101 G. González, T. R. Seastedt. (2001). Soil fauna and plant litter decomposition in tropical and subalpine forests. Ecology. 82(4), 955-964.

102 S. Coq, J.-M. Souquet, E. Meudec, V. Cheynier, S. Hättenschwiler. (2010). Interspecific variation in leaf litter tannins drives decomposition in a tropical rain forest of French Guiana. Ecology. 91(7), 2080-2091.

103 A. E. Hartemink, J. O'sullivan. (2001). Leaf litter decomposition of Piper aduncum, Gliricidia sepium and Imperata cylindrica in the humid lowlands of Papua New Guinea. Plant and Soil. 230(1), 115-124.

104 S. E. Hobbie. (2000). Interactions between litter lignin and soil nitrogen availability during leaf litter decomposition in a Hawaiian montane forest. Ecosystems. 3, 484-494.

105 J. L. Funk. (2005). Hedychium gardnerianum invasion into Hawaiian montane rainforest: interactions among litter quality, decomposition rate, and soil nitrogen availability. Biogeochemistry. 76(3), 441-451.

106 B. Lemma, I. Nilsson, D. B. Kleja, M. Olsson, H. Knicker. (2007). Decomposition and substrate quality of leaf litters and fine roots from three exotic plantations and a native forest in the southwestern highlands of Ethiopia. Soil Biology and Biochemistry. 39(9), 2317-2328.

107 A. Tardif, B. Shipley. (2015). The relationship between functional dispersion of mixed‐species leaf litter mixtures and species' interactions during decomposition. Oikos. 124(8), 1050-1057.

108 B. R. Taylor, D. Parkinson, W. F. Parsons. (1989). Nitrogen and lignin content as predictors of litter decay rates: a microcosm test. Ecology. 70(1), 97-104.

109 X.-M. Zhang, Y.-D. Wang, Y. Zhao, X.-W. Xu, J.-Q. Lei, R. L. Hill. (2017). Litter decomposition and nutrient dynamics of three woody halophytes in the Taklimakan Desert Highway Shelterbelt. Arid Land Research and Management. 31(3), 335-351.

110 X. Geng, J. Pastor, B. Dewey. (1993). Decay and nitrogen dynamics of litter from disjunct, congeneric tree species in old-growth stands in northeastern China and Wisconsin. Canadian Journal of Botany. 71(5), 693-699.

111 G. González, C. M. Murphy, J. Belén. (2012). Direct and indirect effects of millipedes on the decay of litter of varying lignin Content. Tropical Forests. 2, 37-50.

112 N. J. Carnevale, J. P. Lewis. (2009). Leaf litter quality and litter decomposition of woody species in a seasonal subtropical forest (Argentina). Ecotropica. 15, 23-33.

113 P. Zan, T. Sun, Z. Mao. (2021). Effects of soil fauna on litter decomposition using ﬁeld microcosms across 16 co-occurring temperate tree species. Australian Forestry. 84(1), 33-38.

114 O. Butenschoen, V. Krashevska, M. Maraun, F. Marian, D. Sandmann, S. Scheu. (2014). Litter mixture effects on decomposition in tropical montane rainforests vary strongly with time and turn negative at later stages of decay. Soil Biology and Biochemistry. 77, 121-128.

115 Y. Liu, Y. Chen, J. Zhang, W. Yang, Z. Peng, X. He, C. Deng, R. He. (2016). Changes in foliar litter decomposition of woody plants with elevation across an alpine forest–tundra ecotone in eastern Tibet Plateau. Plant Ecology. 217(5), 495-504.

116 A. H. Magill, J. D. Aber. (1998). Long-term effects of experimental nitrogen additions on foliar litter decay and humus formation in forest ecosystems. Plant and Soil. 203(2), 301-311.

117 M. Carreiro, R. Sinsabaugh, D. Repert, D. Parkhurst. (2000). Microbial enzyme shifts explain litter decay responses to simulated nitrogen deposition. Ecology. 81(9), 2359-2365.

118 J. Kumar, P. Sajish, R. N. Kumar, R. K. Bhoi. (2010). Wood and leaf litter decomposition and nutrient release from Tectona grandis Linn. f. in a tropical dry deciduous forest of Rajasthan, Western India. Journal of Forest and Environmental Science. 26(1), 17-23.

119 G. G. Dossa, E. Paudel, K. Cao, D. Schaefer, R. D. Harrison. (2016). Factors controlling bark decomposition and its role in wood decomposition in five tropical tree species. Scientific Reports. 6(1), 1-9.

120 C. Walela, H. Daniel, B. Wilson, P. Lockwood, A. Cowie, S. Harden. (2014). The initial lignin: nitrogen ratio of litter from above and below ground sources strongly and negatively influenced decay rates of slowly decomposing litter carbon pools. Soil Biology and Biochemistry. 77, 268-275.

121 L. Wind, W. Hole. (2013). Initial chemistry and decay dynamics of deciduous leaf litter from a long-term soil-warming study in the Northeastern US. Meadville. Allegheny College.

122 G. Tian, F. Salako, F. Ishida. (2001). Replenishment of C, N, and P in a degraded alfisol under humid tropical conditions: effect of fallow species and litter polyphenols. Soil Science. 166(9), 614-621.

123 J. T. Anderson, L. M. Smith. (2002). The effect of flooding regimes on decomposition of Polygonum pensylvanicum in playa wetlands (Southern Great Plains, USA). Aquatic Botany. 74(2), 97-108.

124 G. Girisha, L. Condron, P. Clinton, M. Davis. (2003). Decomposition and nutrient dynamics of green and freshly fallen radiata pine (Pinus radiata) needles. Forest Ecology and Management. 179(1-3), 169-181.

125 G. Loranger, J.-F. Ponge, P. Lavelle. (2003). Humus forms in two secondary semi‐evergreen tropical forests. European Journal of Soil Science. 54(1), 17-24.

126 A. S. Moretto, R. A. Distel. (2003). Decomposition of and nutrient dynamics in leaf litter and roots of Poa ligularis and Stipa gyneriodes. Journal of Arid Environments. 55(3), 503-514.

127 C. A. d. Oliveira, M. R. S. Muzzi, H. A. Purcino, I. E. Marriel, N. M. H. d. Sá. (2003). Decomposition of Arachis pintoi and Hyparrhenia rufa litters in monoculture and intercropped systems under lowland soil. Pesquisa Agropecuária Brasileira. 38, 1089-1095.

128 C. E. Prescott, G. D. Hope, L. L. Blevins. (2003). Effect of gap size on litter decomposition and soil nitrate concentrations in a high-elevation spruce fir forest. Canadian Journal of Forest Research. 33(11), 2210-2220.

129 B. Reyes-Reyes, E. Zamora-Villafranco, M. Reyes-Reyes, J. Frías-Hernández, V. Olalde-Portugal, L. Dendooven. (2003). Decomposition of leaves of huisache (Acacia tortuoso) and mesquite (Prosopis spp) in soil of the central highlands of Mexico. Plant and Soil. 256(2), 359-370.

130 E. F. Salamanca, N. Kaneko, S. Katagiri. (2003). Rainfall manipulation effects on litter decomposition and the microbial biomass of the forest floor. Applied Soil Ecology. 22(3), 271-281.

131 S. N. Sall, D. Masse, F. Bernhard-Reversat, A. Guisse, J.-L. Chotte. (2003). Microbial activities during the early stage of laboratory decomposition of tropical leaf litters: the effect of interactions between litter quality and exogenous inorganic nitrogen. Biology and Fertility of Soils. 39(2), 103-111.

132 T. Sariyildiz, J. Anderson. (2003). Decomposition of sun and shade leaves from three deciduous tree species, as affected by their chemical composition. Biology and Fertility of Soils. 37(3), 137-146.

133 A. Arunachalam, N. Singh. (2004). Decomposition of Mesua ferrea litter in humid tropics of Arunachal Pradesh, India. Journal of Tropical Forest Science. 16(2), 151-159.

134 R. Chintu, A. Zaharah, A. Wan Rasidah. (2004). Decomposition and nitrogen release patterns of Paraserianthes falcataria tree residues under controlled incubation. Agroforestry Systems. 63(1), 45-52.

135 J. Gindaba, M. Olsson, F. Itanna. (2004). Nutrient composition and short-term release from Croton macrostachyus Del. and Millettia ferruginea (Hochst.) Baker leaves. Biology and fertility of soils. 40(6), 393-397.

136 A. Heim, B. Frey. (2004). Early stage litter decomposition rates for Swiss forests. Biogeochemistry. 70(3), 299-313.

137 S. E. Hobbie, L. Gough. (2004). Litter decomposition in moist acidic and non-acidic tundra with different glacial histories. Oecologia. 140(1), 113-124.

138 L. M. S. Galindo. (2021). Impacts of leaf litter diversity and root resources on microorganisms and microarthropods (Acari, Collembola) during early stages of decomposition in tropical montane rainforest ecosystems. Georg-August-Universität Göttingen.

139 T. Sariyildiz. (2008). Effects of gap-size classes on long-term litter decomposition rates of beech, oak and chestnut species at high elevations in Northeast Turkey. Ecosystems. 11(6), 841-853.

140 T. Sariyildiz, M. Küçük. (2009). Influence of slope position, stand type and rhododendron (Rhododendron ponticum) on litter decomposition rates of Oriental beech (Fagus orientalis Lipsky.) and spruce [Picea orientalis (L.) Link]. European Journal of Forest Research. 128(4), 351-360.

141 T. C. Sarker, G. Maisto, A. De Marco, F. Esposito, S. C. Panico, M. F. Alam, S. Mazzoleni, G. Bonanomi. (2019). Explaining trajectories of chemical changes during decomposition of tropical litter by 13C-CPMAS NMR, proximate and nutrients analysis. Plant and Soil. 436(1), 13-28.

142 B. Berg, H. Staaf, B. Wessen. (1987). Decomposition and nutrient release in needle litter from nitrogen‐fertilized Scots pine (Pinus sylvestris) stands. Scandinavian Journal of Forest Research. 2(1-4), 399-415.

143 M. Semmartin, L. A. Garibaldi, E. J. Chaneton. (2008). Grazing history effects on above-and below-ground litter decomposition and nutrient cycling in two co-occurring grasses. Plant and Soil. 303(1), 177-189.

144 R. Semwal, R. Maikhuri, K. Rao, K. Sen, K. Saxena. (2003). Leaf litter decomposition and nutrient release patterns of six multipurpose tree species of central Himalaya, India. Biomass and Bioenergy. 24(1), 3-11.

145 N. N. Setiawan, M. Vanhellemont, A. De Schrijver, S. Schelfhout, L. Baeten, K. Verheyen. (2016). Mixing effects on litter decomposition rates in a young tree diversity experiment. Acta Oecologica. 70, 79-86.

146 F. Wu, W. Yang, J. Zhang, R. Deng. (2010). Litter decomposition in two subalpine forests during the freeze–thaw season. Acta Oecologica. 36(1), 135-140.

147 T. Li-Hua, H. Hong-Ling, H. Ting-Xing, J. ZHANG, L. Xian-Wei, L. Li, X. Yin-Long, C. Gang, L. Ren-Hong. (2014). Litterfall, litter decomposition, and nutrient dynamics in two subtropical bamboo plantations of China. Pedosphere. 24(1), 84-97.

148 M. C. Mack, C. M. D’Antonio. (2003). The effects of exotic grasses on litter decomposition in a Hawaiian woodland: the importance of indirect effects. Ecosystems. 6(8), 723-738.

149 A. Pastor, Z. G. Compson, P. Dijkstra, J. L. Riera, E. Martí, F. Sabater, B. A. Hungate, J. C. Marks. (2014). Stream carbon and nitrogen supplements during leaf litter decomposition: contrasting patterns for two foundation species. Oecologia. 176(4), 1111-1121.

150 T. Sariyildiz. (2003). Litter decomposition of Picea orientalis, Pinus sylvestris and Castanea sativa trees grown in Artvin in relation to their initial litter quality variables. Turkish Journal of Agriculture and Forestry. 27(4), 237-243.

151 I. Naeem, T. Asif, X. Wu, N. Hassan, L. Yiming, H. Wang, L. Wang, D. Wang. (2021). Species diversity induces idiosyncratic effects on litter decomposition in a degraded meadow steppe. Frontiers in Environmental Science. 13.

152 X. Zhang, H. Lei, Y. Chong, J. Hu, W. Che, M. Hu, S. Xu, P. Zhang, L. Zhang, J. Xu. (2019). Do silvi-medicinal plantations affect tree litter decomposition and nutrient mineralization? Sustainability. 11(18), 5138.

153 X. Song, G. Zhou, H. Gu, L. Qi. (2015). Management practices amplify the effects of N deposition on leaf litter decomposition of the Moso bamboo forest. Plant and Soil. 395(1), 391-400.

154 Y. Song, C. Song, J. Ren, W. Tan, S. Jin, L. Jiang. (2018). Influence of nitrogen additions on litter decomposition, nutrient dynamics, and enzymatic activity of two plant species in a peatland in Northeast China. Science of the Total Environment. 625, 640-646.

155 T. Sariyildiz, A. TÜFEKÇİOĞLU, M. Küçük. (2005). Comparison of decomposition rates of beech (Fagus orientalis Lipsky) and spruce (Picea orientalis (L.) Link) litter in pure and mixed stands of both species in Artvin, Turkey. Turkish Journal of Agriculture and Forestry. 29(6), 429-438.

156 Y. Jia, X. Kong, M. D. Weiser, Y. Lv, S. Akbar, X. Jia, K. Tian, Z. He, H. Lin, Z. Bei. (2015). Sodium limits litter decomposition rates in a subtropical forest: additional tests of the sodium ecosystem respiration hypothesis. Applied Soil Ecology. 93, 98-104.

157 P. Wang, J. van Ruijven, M. M. Heijmans, F. Berendse, A. Maksimov, T. Maximov, L. Mommer. (2017). Short-term root and leaf decomposition of two dominant plant species in a Siberian tundra. Pedobiologia. 65, 68-76.

158 S. Singh, K. Pande, V. Upadhyay, J. Singh. (1990). Fungal communities associated with the decomposition of a common leaf litter (Quercus leucotrichophora A. Camus) along an elevational transect in the Central Himalaya. Biology and Fertility of Soils. 9(3), 245-251.

159 X. Song, H. Jiang, Z. Zhang, G. Zhou, S. Zhang, C. Peng. (2014). Interactive effects of elevated UV-B radiation and N deposition on decomposition of Moso bamboo litter. Soil Biology and Biochemistry. 69, 11-16.

160 Y.-Y. Song, C.-C. Song, J.-S. Ren, X.-H. Zhang, L. Jiang. (2019). Nitrogen input increases Deyeuxia angustifolia litter decomposition and enzyme activities in a marshland ecosystem in Sanjiang plain, northeast China. Wetlands. 39(3), 549-557.

161 A. S. Poi, M. E. Galassi, R. P. Carnevali, L. I. Gallardo. (2017). Leaf litter and invertebrate colonization: the role of macroconsumers in a Subtropical wetland (Corrientes, Argentina). Wetlands. 37(1), 135-143.

162 M. I. Rashid, T. Shahzad, M. Shahid, I. M. Ismail, G. M. Shah, T. Almeelbi. (2017). Zinc oxide nanoparticles affect carbon and nitrogen mineralization of Phoenix dactylifera leaf litter in a sandy soil. Journal of Hazardous Materials. 324, 298-305.

163 X. Song, Q. Li, H. Gu. (2017). Effect of nitrogen deposition and management practices on fine root decomposition in Moso bamboo plantations. Plant and Soil. 410(1), 207-215.

164 L. Yahdjian, P. M. Tognetti, E. J. Chaneton. (2017). Plant functional composition affects soil processes in novel successional grasslands. Functional Ecology. 31(9), 1813-1823.

165 J. Zheng, Y. Wang, N. Hui, H. Dong, C. Chen, S. Han, Z. Xu. (2017). Changes in CH4 production during different stages of litter decomposition under inundation and N addition. Journal of Soils and Sediments. 17(4), 949-959.

166 A. K. Anning, B. Gyamfi, A. T. Effah. (2018). Broussonetia papyrifera controls nutrient return to soil to facilitate its invasion in a tropical forest of Ghana. Journal of Plant Ecology. 11(6), 909-918.

167 A. Figueiredo, F. Augusto, L. D. Coletta, P. Duarte-Neto, E. Mazzi, L. Martinelli. (2018). Comparison of microbial processing of Brachiaria brizantha, a C4 invasive species and a rainforest species in tropical streams of the Atlantic Forest of south-eastern Brazil. Marine and Freshwater Research. 69(9), 1397-1407.

168 K. Hoeffner, C. Monard, M. Santonja, D. Cluzeau. (2018). Feeding behaviour of epi-anecic earthworm species and their impacts on soil microbial communities. Soil Biology and Biochemistry. 125, 1-9.

169 C.-h. Huangfu, Z.-s. Wei. (2018). Nitrogen addition drives convergence of leaf litter decomposition rates between Flaveria bidentis and native plant. Plant Ecology. 219(11), 1355-1368.

170 Y. Jiang, J. Wang, S. Muhammad, R. Hao, Y. Wu. (2018). How do earthworms affect decomposition of residues with different quality apart from fragmentation and incorporation? Geoderma. 326, 68-75.

171 L. Jiang, L. Kou, S. Li. (2018). Alterations of early-stage decomposition of leaves and absorptive roots by deposition of nitrogen and phosphorus have contrasting mechanisms. Soil Biology and Biochemistry. 127, 213-222.

172 L. Li, M. Xu, M. Eyakub Ali, W. Zhang, Y. Duan, D. Li. (2018). Factors affecting soil microbial biomass and functional diversity with the application of organic amendments in three contrasting cropland soils during a field experiment. PLoS One. 13(9), e0203812.

173 B. Mao, L. Zhao, Q. Zhao, D. Zeng. (2018). Effects of ultraviolet (UV) radiation and litter layer thickness on litter decomposition of two tree species in a semi-arid site of Northeast China. Journal of Arid Land. 10(3), 416-428.

174 E. W. Morrison, A. Pringle, L. T. van Diepen, S. D. Frey. (2018). Simulated nitrogen deposition favors stress-tolerant fungi with low potential for decomposition. Soil Biology and Biochemistry. 125, 75-85.

175 S. K. Naik, S. Maurya, D. Mukherjee, A. Singh, B. Bhatt. (2018). Rates of decomposition and nutrient mineralization of leaf litter from different orchards under hot and dry sub-humid climate. Archives of Agronomy and Soil Science. 64(4), 560-573.

176 L. Chao, Y. Liu, G. T. Freschet, W. Zhang, X. Yu, W. Zheng, X. Guan, Q. Yang, L. Chen, F. A. Dijkstra. (2019). Litter carbon and nutrient chemistry control the magnitude of soil priming effect. Functional Ecology. 33(5), 876-888.

177 K. Fujii, C. Hayakawa, Y. Inagaki, T. Kosaki. (2020). Effects of land use change on turnover and storage of soil organic matter in a tropical forest. Plant and Soil. 446(1), 425-439.

178 D. Kerdraon, J. Drewer, B. Castro, A. Wallwork, J. S. Hall, E. J. Sayer. (2019). Litter traits of native and non-native tropical trees influence soil carbon dynamics in timber plantations in Panama. Forests. 10(3), 209.

179 C. Wu, H. Wang, Q. Mo, Z. Zhang, G. Huang, F. Kong, Y. Liu, G. G. Wang. (2019). Effects of elevated UV-B radiation and N deposition on the decomposition of coarse woody debris. Science of the Total Environment. 663, 170-176.

180 Y. Xie, Y. Xie, H. Xiao, X. Chen, F. Li. (2019). The effects of simulated inundation duration and frequency on litter decomposition: A one-year experiment. Limnologica. 74, 8-13.

181 X.-M. Ye, Y. Zhang, F.-S. Chen, G. G. Wang, X.-M. Fang, X.-F. Lin, S.-Z. Wan, P. He. (2019). The effects of simulated deposited nitrogen on nutrient dynamics in decomposing litters across a wide quality spectrum using a 15N tracing technique. Plant and Soil. 442(1), 141-156.

182 X. Yu, L. Chao, W. Zhang, L. Chen, Q. Yang, G. Zhang, S. Wang. (2019). Effects of inorganic nitrogen and litters of Masson Pine on soil organic carbon decomposition. PLoS One. 14(9), e0222973.

183 J. Cao, X. He, Y. Chen, Y. Chen, Y. Zhang, S. Yu, L. Zhou, Z. Liu, C. Zhang, S. Fu. (2020). Leaf litter contributes more to soil organic carbon than fine roots in two 10-year-old subtropical plantations. Science of the Total Environment. 704, 135341.

184 P. Pavlović, O. Kostić, S. Jarić, G. Gajić, D. Pavlović, M. Pavlović, M. Mitrović. (2020). The effects of leaf litter chemistry and anatomical traits on the litter decomposition rate of Quercus frainetto Ten. and Quercus cerris L. in situ. Archives of Biological Sciences. 72(4), 543-553.

185 D. Ross, K. Tate, P. Newton, H. Clark. (2002). Decomposability of C3 and C4 grass litter sampled under different concentrations of atmospheric carbon dioxide at a natural CO2 spring. Plant and Soil. 240(2), 275-286.

186 L. Serrano, J. Penuelas, S. L. Ustin. (2002). Remote sensing of nitrogen and lignin in Mediterranean vegetation from AVIRIS data: Decomposing biochemical from structural signals. Remote sensing of Environment. 81(2-3), 355-364.

187 D. E. Rothstein, P. M. Vitousek, B. L. Simmons. (2004). An exotic tree alters decomposition and nutrient cycling in a Hawaiian montane forest. Ecosystems. 7(8), 805-814.

188 M. Růžek, K. Tahovská, G. Guggenberger, F. Oulehle. (2021). Litter decomposition in European coniferous and broadleaf forests under experimentally elevated acidity and nitrogen addition. Plant and Soil. 463(1), 471-485.

189 Z. Koukoura, A. Mamolos, K. Kalburtji. (2003). Decomposition of dominant plant species litter in a semi-arid grassland. Applied Soil Ecology. 23(1), 13-23.

190 Y.-j. Xie, Y.-h. Xie, H.-y. Xiao, Z.-m. Deng, Y. Pan, B.-h. Pan, J.-y. Hu. (2017). Inhibition of litter decomposition of two emergent macrophytes by addition of aromatic plant powder. Scientific Reports. 7(1), 1-8.

191 L. Gong, X. Chen, X. Zhang, X. Yang, Y. Cai. (2020). Schrenk spruce leaf litter decomposition varies with snow depth in the Tianshan Mountains. Scientific Reports. 10(1), 1-12.

192 S. Hättenschwiler, P. Gasser. (2005). Soil animals alter plant litter diversity effects on decomposition. Proceedings of the National Academy of Sciences. 102(5), 1519-1524.

193 H. Staaf. (1987). Foliage litter turnover and earthworm populations in three beech forests of contrasting soil and vegetation types. Oecologia. 72(1), 58-64.

194 J. G. Cepeda-Pizarro. (1993). Litter decomposition in deserts: an overview with an example from coastal arid Chile. Revista Chilena de Historia Natural. 66, 323-336.

195 M. Badejo, T. Nathaniel, G. Tian. (1998). Abundance of springtails (Collembola) under four agroforestry tree species with contrasting litter quality. Biology and Fertility of Soils. 27(1), 15-20.

196 F. Bernhard-Reversat. (1998). Changes in relationships between initial litter quality and CO2 release during early laboratory decomposition of tropical leaf litters. European Journal of Soil Biology. 34(3), 117-122.

197 A. Fioretto, A. Musacchio, G. Andolfi, A. V. De Santo. (1998). Decomposition dynamics of litters of various pine species in a Corsican pine forest. Soil Biology and Biochemistry. 30(6), 721-727.

198 F. R. Gahrooee. (1998). Impacts of elevated atmospheric CO2 on litter quality, litter decomposability and nitrogen turnover rate of two oak species in a Mediterranean forest ecosystem. Global Change Biology. 4(6), 667-677.

199 B. M. Kumar, J. K. Deepu. (1992). Litter production and decomposition dynamics in moist deciduous forests of the Western Ghats in Peninsular India. Forest Ecology and Management. 50(3-4), 181-201.

200 K. Thomas, C. Prescott. (2000). Nitrogen availability in forest floors of three tree species on the same site: the role of litter quality. Canadian Journal of Forest Research. 30(11), 1698-1706.

201 G. Tian, J. Olimah, G. Adeoye, B. Kang. (2000). Regeneration of earthworm populations in a degraded soil by natural and planted fallows under humid tropical conditions. Soil Science Society of America Journal. 64(1), 222-228.

202 L. Guo, R. Sims. (2002). Eucalypt litter decomposition and nutrient release under a short rotation forest regime and effluent irrigation treatments in New Zealand: II. Internal effects. Soil Biology and Biochemistry. 34(7), 913-922.

203 R. T. Martins, A. S. Melo, J. F. Gonçalves, C. M. Campos, N. Hamada. (2017). Effects of climate change on leaf breakdown by microorganisms and the shredder Phylloicus elektoros (Trichoptera: Calamoceratidae). Hydrobiologia. 789(1), 31-44.

204 A. C. Buch, C. L. S. Sisinno, M. E. F. Correia, E. V. Silva-Filho. (2018). Food preference and ecotoxicological tests with millipedes in litter contaminated with mercury. Science of the Total Environment. 633, 1173-1182.

205 D. Chellaiah, C. M. Yule. (2018). Litter decomposition is driven by microbes and is more influenced by litter quality than environmental conditions in oil palm streams with different riparian types. Aquatic Sciences. 80(4), 1-11.

206 T. Tanikawa, S. Fujii, L. Sun, Y. Hirano, Y. Matsuda, K. Miyatani, R. Doi, T. Mizoguchi, N. Maie. (2018). Leachate from fine root litter is more acidic than leaf litter leachate: a 2.5-year laboratory incubation. Science of the Total Environment. 645, 179-191.

207 B. A. Ball, M. P. Christman, S. J. Hall. (2019). Nutrient dynamics during photodegradation of plant litter in the Sonoran Desert. Journal of Arid Environments. 160, 1-10.

208 C. Biasi, G. Cogo, L. Hepp, S. Santos. (2019). Grass species as a source of allochthonous energy for shredders and fungal decomposers in a subtropical stream. Fundamental and Applied Limnology. 192, 331-341.

209 C. Biasi, G. B. Cogo, L. U. Hepp, S. Santos. (2019). Shredders prefer soft and fungal-conditioned leaves, regardless of their initial chemical traits. Iheringia. Série Zoologia. 109,

210 M. He, R. Zhao, Q. Tian, L. Huang, X. Wang, F. Liu. (2019). Predominant effects of litter chemistry on lignin degradation in the early stage of leaf litter decomposition. Plant and Soil. 442(1), 453-469.

211 V. C. Vos, J. van Ruijven, M. P. Berg, E. T. Peeters, F. Berendse. (2011). Macro‐detritivore identity drives leaf litter diversity effects. Oikos. 120(7), 1092-1098.

212 H. M. Siqueira da Silva, J. C. Batista Dubeux Jr, M. L. Silveira, M. V. Ferreira dos Santos, E. Viana de Freitas, M. de Andrade Lira. (2019). Root decomposition of grazed signalgrass in response to stocking and nitrogen fertilization rates. Crop Science. 59(2), 811-818.

213 J. M. Klopatek. (2008). Litter decomposition contrasts in second-and old-growth Douglas-fir forests of the Pacific Northwest, USA. Plant Ecology. 196(1), 123-133.

214 E. Gacheru, M. Rao. (2001). Managing Striga infestation on maize using organic and inorganic nutrient sources in western Kenya. International Journal of Pest Management. 47(3), 233-239.

215 A. Kwabiah, N. Stoskopf, R. Voroney, C. Palm. (2001). Nitrogen and Phosphorus Release from Decomposing Leaves under Sub‐Humid Tropical Conditions 1. Biotropica. 33(2), 229-240.

216 A. S. Moretto, R. A. Distel, N. G. Didone. (2001). Decomposition and nutrient dynamic of leaf litter and roots from palatable and unpalatable grasses in a semi-arid grassland. Applied Soil Ecology. 18(1), 31-37.

217 H. D. Arato, L. E. Dias, I. R. d. Assis, N. F. d. Barros, J. C. L. Neves, C. E. G. R. Schaefer. (2018). Leaf residue decomposition of selected atlantic forest tree species. Revista Árvore. 41,

218 C. Biasi, M. A. Graça, S. Santos, V. Ferreira. (2017). Nutrient enrichment in water more than in leaves affects aquatic microbial litter processing. Oecologia. 184(2), 555-568.

219 D. Čiuldienė, J. Aleinikovienė, M. Muraškienė, V. Marozas, K. Armolaitis. (2017). Release and retention patterns of organic compounds and nutrients after the cold period in foliar litterfall of pure European larch, common beech and red oak plantations in Lithuania. Eurasian Soil Science. 50(1), 49-56.

220 E. M. Corteselli, J. C. Burtis, A. K. Heinz, J. B. Yavitt. (2017). Leaf litter fuels methanogenesis throughout decomposition in a forested peatland. Ecosystems. 20(6), 1217-1232.

221 H. Li, Z. Wei, C. Huangfu, X. Chen, D. Yang. (2017). Litter mixture dominated by leaf litter of the invasive species, Flaveria bidentis, accelerates decomposition and favors nitrogen release. Journal of Plant Research. 130(1), 167-180.

222 D. Luo, R. Cheng, Z. Shi, W. Wang. (2017). Decomposition of leaves and fine roots in three subtropical plantations in China affected by litter substrate quality and soil microbial community. Forests. 8(11), 412.

223 B. Mao, R. Mao, D.-H. Zeng. (2017). Species diversity and chemical properties of litter influence non-additive effects of litter mixtures on soil carbon and nitrogen cycling. PLoS One

224 K. E. White, F. J. Coale, J. B. Reeves III. (2018). Degradation changes in plant root cell wall structural molecules during extended decomposition of important agricultural crop and forage species. Organic Geochemistry. 115, 233-245.

225 G. Zhou, J. Zhang, X. Qiu, F. Wei, X. Xu. (2018). Decomposing litter and associated microbial activity responses to nitrogen deposition in two subtropical forests containing nitrogen-fixing or non-nitrogen-fixing tree species. Scientific Reports. 8(1), 1-11.

226 J. Azeez. (2019). Recycling organic waste in managed tropical forest ecosystems: effects of arboreal litter types on soil chemical properties in Abeokuta, southwestern Nigeria. Journal of Forestry Research. 30(5), 1903-1911.

227 N. R. Murovhi, S. A. Materechera. (2015). Decomposition of subtropical fruit tree leaf litter at Nelspruit, South Africa. Communications in Soil Science and Plant Analysis. 46(7), 859-872.

228 B. Dhanya, S. Viswanath, S. Purushothaman. (2013). Decomposition and nutrient release dynamics of Ficus benghalensis L. litter in traditional agroforestry systems of Karnataka, Southern India. International Scholarly Research Notices. 2013,

229 F. P. Day Jr. (1982). Litter decomposition rates in the seasonally flooded Great Dismal Swamp. Ecology. 63(3), 670-678.

230 T. Osono, J.-i. Azuma, D. Hirose. (2014). Plant species effect on the decomposition and chemical changes of leaf litter in grassland and pine and oak forest soils. Plant and Soil. 376(1), 411-421.

231 Y. Xie, Y. Xie, C. Hu, X. Chen, F. Li. (2016). Interaction between litter quality and simulated water depth on decomposition of two emergent macrophytes. Journal of Limnology. 75(1),

232 N. E. Pettit, T. Davies, J. B. Fellman, P. F. Grierson, D. M. Warfe, P. M. Davies. (2012). Leaf litter chemistry, decomposition and assimilation by macroinvertebrates in two tropical streams. Hydrobiologia. 680(1), 63-77.

233 W.-F. Cong, J. van Ruijven, W. van der Werf, G. B. De Deyn, L. Mommer, F. Berendse, E. Hoffland. (2015). Plant species richness leaves a legacy of enhanced root litter-induced decomposition in soil. Soil Biology and Biochemistry. 80, 341-348.

234 J. DeMarco, M. C. Mack, M. S. Bret-Harte. (2014). Effects of arctic shrub expansion on biophysical vs. biogeochemical drivers of litter decomposition. Ecology. 95(7), 1861-1875.

235 N. KIZILDAĞ, C. Darici, H. A. SAĞLIKER. (2014). Influence of different parent materials on litter decomposition in the east mediterranean region. Pakistan Journal of Botany. 46(3), 875-879.

236 A. O'Connell. (1988). Decomposition of leaf litter in karri (Eucalyptus diversicolor) forest of varying age. Forest Ecology and Management. 24(2), 113-125.

237 J. O. Klemmedson. (1992). Decomposition and nutrient release from mixtures of Gambel oak and ponderosa pine leaf litter. Forest Ecology and Management. 47(1-4), 349-361.

238 Y. Chen, Y. Liu, J. Zhang, W. Yang, R. He, C. Deng. (2018). Microclimate exerts greater control over litter decomposition and enzyme activity than litter quality in an alpine forest-tundra ecotone. Scientific Reports. 8(1), 1-13.

239 F. Maunoury-Danger, V. Felten, C. Bojic, F. Fraysse, M. Cosin Ponce, O. Dedourge-Geffard, A. Geffard, F. Guérold, M. Danger. (2018). Metal release from contaminated leaf litter and leachate toxicity for the freshwater crustacean Gammarus fossarum. Environmental Science and Pollution Research. 25(12), 11281-11294.

240 H. H. L. Saulino, R. M. Thompson, S. Trivinho-Strxino. (2018). Herbivore functional traits and macroinvertebrate food webs have different responses to leaf chemical compounds of two macrophyte species in a tropical lake’s littoral zone. Aquatic Ecology. 52(2), 165-176.

241 J. M. Melillo, J. D. Aber, J. F. Muratore. (1982). Nitrogen and lignin control of hardwood leaf litter decomposition dynamics. Ecology. 63(3), 621-626.

242 T. A. Day, M. S. Bliss, A. R. Tomes, C. T. Ruhland, R. Guénon. (2018). Desert leaf litter decay: Coupling of microbial respiration, water‐soluble fractions and photodegradation. Global Change Biology. 24(11), 5454-5470.

243 W. Zhu, J. Wang, Z. Zhang, F. Ren, L. Chen, J.-S. He. (2016). Changes in litter quality induced by nutrient addition alter litter decomposition in an alpine meadow on the Qinghai-Tibet Plateau. Scientific Reports. 6(1), 1-10.

244 J. B. Winbourne, B. Z. Houlton. (2018). Plant‐soil feedbacks on free‐living nitrogen fixation over geological time. Ecology. 99(11), 2496-2505.

245 Z. Yang, W. Song, Y. Zhao, J. Zhou, Z. Wang, Y. Luo, Y. Li, G. Lin. (2018). Differential responses of litter decomposition to regional excessive nitrogen input and global warming between two mangrove species. Estuarine, Coastal and Shelf Science. 214, 141-148.

246 C. Wang, G. Han, Y. Jia, X. Feng, P. Guo, X. Tian. (2011). Response of litter decomposition and related soil enzyme activities to different forms of nitrogen fertilization in a subtropical forest. Ecological Research. 26(3), 505-513.

247 C. E. Riggs, S. E. Hobbie, J. Cavender-Bares, J. A. Savage, X. Wei. (2015). Contrasting effects of plant species traits and moisture on the decomposition of multiple litter fractions. Oecologia. 179(2), 573-584.

248 S. Rouifed, I. T. Handa, J.-F. David, S. Hättenschwiler. (2010). The importance of biotic factors in predicting global change effects on decomposition of temperate forest leaf litter. Oecologia. 163(1), 247-256.

249 W. He, W. Yang. (2020). Loss of total phenols from leaf litter of two shrub species: dual responses to alpine forest gap disturbance during winter and the growing season. Journal of Plant Ecology. 13(3), 369-377.

250 X.-z. Song, H. Jiang, H.-l. Zhang, C.-h. Peng, S.-q. Yu. (2011). Elevated UV-B radiation did not affect decomposition rates of needles of two coniferous species in subtropical China. European Journal of Soil Biology. 47(6), 343-348.

251 F. Trum, H. Titeux, J. Ranger, B. Delvaux. (2011). Influence of tree species on carbon and nitrogen transformation patterns in forest floor profiles. Annals of Forest Science. 68(4), 837-847.

252 S. M. Uselman, K. A. Snyder, R. R. Blank, T. J. Jones. (2011). UVB exposure does not accelerate rates of litter decomposition in a semi-arid riparian ecosystem. Soil Biology and Biochemistry. 43(6), 1254-1265.

253 L. Vivanco, A. T. Austin. (2011). Nitrogen addition stimulates forest litter decomposition and disrupts species interactions in Patagonia, Argentina. Global Change Biology. 17(5), 1963-1974.

254 C. M. Prather, G. E. Belovsky, S. A. Cantrell, G. González. (2018). Tropical herbivorous phasmids, but not litter snails, alter decomposition rates by modifying litter bacteria. Ecology. 99(4), 782-791.

255 R. C. Cobb, D. M. Rizzo. (2016). Litter chemistry, community shift, and non-additive effects drive litter decomposition changes following invasion by a generalist pathogen. Ecosystems. 19(8), 1478-1490.

256 M. E. Harmon, G. A. Baker, G. Spycher, S. E. Greene. (1990). Leaf-litter decomposition in the Picea/tsuga forests of Olympic National Park, Washington, USA. Forest Ecology and Management. 31(1-2), 55-66.

257 W. Purahong, D. Kapturska, M. J. Pecyna, E. Schulz, M. Schloter, F. Buscot, M. Hofrichter, D. Krüger. (2014). Influence of different forest system management practices on leaf litter decomposition rates, nutrient dynamics and the activity of ligninolytic enzymes: a case study from Central European forests. PLoS One. 9(4), e93700.

258 N. Mehta, J. Dinakaran, S. Patel, A. Laskar, M. Yadava, R. Ramesh, N. Krishnayya. (2013). Changes in litter decomposition and soil organic carbon in a reforested tropical deciduous cover (India). Ecological Research. 28(2), 239-248.

259 A. Rai, A. K. Singh, N. Singh, N. Ghoshal. (2020). Effect of elevated CO2 on litter functional traits, mass loss and nutrient release of two subtropical species in free air carbon enrichment facility. Environmental and Experimental Botany. 172, 103994.

260 C. de Toledo Castanho, A. A. de Oliveira. (2008). Relative effect of litter quality, forest type and their interaction on leaf decomposition in south-east Brazilian forests. Journal of Tropical Ecology. 24(2), 149-156.

261 Y. Liu, G. Li. (2008). Decomposition characteristics affected by forest age in pinus tabulaeformis plantations. Forest Research. 21(4), 500-505. (in Chinese with English abstract)

262 Y. Xiang, C. Huang, T. Hu, L. Tu, L. Yuan, Z. Zhang, W. Xu. (2015). Changes in masses of substrates during litter decomposition in Eucalyptus grandis plantations with different densities. Journal of Northwest A&F University (Natural Science Edution). 43(4), 65-72. (in Chinese with English abstract)

263 W. Xiao, J. Diao, F. Fei, Q. Guan. (2016). Effects of different intensity thinning on litter decomposition in Chinese fir plantations. Ecology and Environmental Sciences. 25(8), 1291-1299. (in Chinese with English abstract)

264 L. Wang, R. He, L. Yang, Y. Chen, Y. Liu, J. Zhang. (2016). Contribution of soil fauna to litter decomposition of Abies faxoniana and Rhododendron lapponicum across an alpine timberline ecotone in Western Sichuan, China. Chinese Journal of Applied Ecology. 27(11), 3689-3697. (in Chinese with English abstract)

265 R. Liu, W. Yang, F. Wu, B. Tan, W. Wang. (2014). Soil fauna community structure and diversity during foliar litter decomposition in the subalpine/alpine forests of western Sichuan. Chinese Journal of Applied and Environmental Biology. 20(3), 499-507. (in Chinese with English abstract)

266 L. Lan, W. Yang, F. Wu, Y. Liu, F. Yang, C. Guo, Y. Cheng, B. Tan. (2019). Effects of soil fauna on microbial community during litter decomposition of Populus simonii and Fargesia spathacea in the subalpine forest of western Sichuan, China. Chinese Journal of Applied Ecology. 30(9), 2983-2991. (in Chinese with English abstract)

267 Y. Shi, F. Chen. (2016). Litter decomposition and nutrient release of Larix kaempferi forest in Dalaoling Nature Reserve. Forest Research. 29(3), 430-435. (in Chinese with English abstract)

268 K. Li. (2007). Effect of nitrogen deposition on litter decomposition of two main coniferous tree species in Changbai Mountain. Journal of Northeast Forestry University. 35(2), 17-19. (in Chinese with English abstract)

269 J. Zheng, R. Guo, D. Li, D. Li, J. Li, B. Zhu, S. Han. (2016). Effects of nitrogen deposition and drought on litter decomposition in a temperate forest. Journal of Beijing Forestry University. 38(4), 21-28. (in Chinese with English abstract)

270 Y. Zhang, K. Wang, Y. Song, X. Zheng, Y. Pan, M. Su, X. Li. (2020). Response of litter decomposition of zonal vegetation to simulated nitrogen deposition in central Yunnan, China. Acta Ecologica Sinica. 40(22), 8274-8286. (in Chinese with English abstract)

271 Narigele, Z. Sheng, K. He, Y. Huang. (2018). Effects of nitrogen addition on litter decomposition of two dominant species in temperate steppe, Inner Mongolia. Journal of Beijing Normal University (Natural Science). 54(5), 674-681. (in Chinese with English abstract)

272 Y. Zhang, Y. Song, K. Wang, X. Yang, J. Xing, Z. Zhang. (2020). Responses of litter decomposition in two subalpine plantations to simulated nitrogen deposition in central Yunnan, China. Chinese Journal of Applied Ecology. 31(8), 2523-2532. (in Chinese with English abstract)

273 J. Yang, Z. Zhou, R. Lyu, J. Liang, X. Wang. (2019). Dynamic decomposition of foliar litters of three plant species in arid habitats. Arid Zone Research. 36(4), 916-923. (in Chinese with English abstract)

274 J. Li, X. Sun, Y. Hu, S. Zhao, D. Zeng. (2017). Effects of drying-rewetting on leaf litter decomposition and nutrient releases in forest plantations in Horqin Sandy Land, China. Chinese Journal of Applied Ecology. 28(6), 1743-1752. (in Chinese with English abstract)

275 C. Deng, X. Jiang, Y. Liu, J. Zhang, Y. Chen, R. He. (2015). Litter decomposition of Rhododendron lapponicum in alpine timberline ecotone. Acta Ecologica Sinica. 35(6), 1769-1778. (in Chinese with English abstract)

276 X. Zhou, F. Wu, W. Yang, J. Zhu. (2011). Dynamics of microbial biomass during litter decomposition in the alpine forest. Acta Ecologica Sinica. 31(14), 4144-4152. (in Chinese with English abstract)

277 H. Zhao, J. Cheng, W. Zhang, Y. Su, C. Zhang, J. SHeng. (2020). Litters decomposition characteristics of five species in the Gurbantunggut Desert. Journal of Desert Research. 40(2), 165-176. (in Chinese with English abstract)

278 J. Wu, Q. Yu, D. Liang, J. Zhang, Z. Li, S. Zhang. (2015). Effect of light radiation on litter decomposition in alpine meadow of the Tibetan Plateau. Chinese Journal of Ecology. 34(11), 2990-2994. (in Chinese with English abstract)

279 H. Wen, H. Fu, D. Guo. (2017). Influence of nitrogen addition on Stipa bungeana and Heteropappus altaicus litter decomposition and nutrient release in a steppe located on the Loess Plateau. Acta Ecologica Sinica. 37(6), 2014-2022. (in Chinese with English abstract)

280 C. Li, X. Li, J. Henry, L. Li, L. Lin. (2019). Decomposition Characteristics of Karelinia caspia, Alhagi sparsifolia and Populus euphratica Leaves in Extremely Arid Areas. Journal of Desert Research. 39(2), 193-201. (in Chinese with English abstract)

281 H. Li, G. Yu, J. Li, T. Liang, Y. Chen. (2007). Dynamics of litter decomposition and phosphorus and potassium release in Jinggang Mountain region of Jiangxi Province, China. Chinese Journal of Applied Ecology. 18(2), 233-240. (in Chinese with English abstract)

282 J. Bi, Y. Li, Z. Ning, X. Zhao. (2016). Carbon mineralization and decomposition oof litters from dominant plants in the Horqin Sandy Land: Effects of litter quality. Journal of Desert Research. 36(1), 85-92. (in Chinese with English abstract)

283 Z. Wei, H. Li, K. Li, D. Yang, C. Huangfu. (2017). Effects of simulated N deposition and burial on Flaveria bidentis litter decomposition and nutrient release. Chinese Journal of Ecology. 36(9), 2412-2422. (in Chinese with English abstract)

284 L. Tu, H. Dai, T. Hu, J. Zhang, J. Luo, S. Luo, Y. Cheng. (2011). Effect of simulated nitrogen deposition on litter decomposition in a Bambusa pervariabilis × Dendrocala mopsi plantation, Rainy Area of West China. Acta Ecologica Sinica. 31(5), 1277-1284. (in Chinese with English abstract)

285 Y. Jia, X. Du, W. Wang, X. Kong, X. Tian. (2017). Effects of sodium addition on litter decomposition and soil microbial activities in subtropical forests. Journal of Northeast Forestry University. 45(11), 39-44. (in Chinese with English abstract)

286 Y. Mu, Y. Geng. (2016). The dynamics of nutrients during litter decomposition in Leymus chinensis grassland in Inner Mongolia, China. Ecology and Environmental Sciences. 25(7), 1154-1163. (in Chinese with English abstract)

287 J. Wang, J. Huang. (2001). Comparison of major nutrient release patterns in leaf litter decomposition in warm temperate zone of China. Chinese Journal of Plant Ecology. 25(3), 375-380. (in Chinese with English abstract)

288 L. Huo, M. Hong, B. Zhao, H. Gao, H. Ye. (2018). Effects of water and nitrogen control on the decomposition of litter in desert grassland. Chinese Journal of Grassland. 40(5), 57-63. (in Chinese with English abstract)

289 W. Wang, W. Yang, B. Tan, R. Liu, F. Wu. (2013). The dynamics of soil fauna community during litter decomposition at different phenological stages in the subtropical evergreen broad-leaved forests in Sichuan basin. Acta Ecologica Sinica. 33(18), 5737-5750. (in Chinese with English abstract)

290 X. Zhang, Y. Wang, X. Xu, J. Lei, J. Sun. (2017). Effects of fertilizer addition on surface litter decomposition in the Tarim Desert Highway shelterbelt. Acta Ecologica Sinica. 37(5), 1506-1514. (in Chinese with English abstract)

291 Y. Lu, K. Li, Q. Liang, C. Li, C. Zhang. (2019). Effects of leaf litter decomposition on bacterial community structure in the leaf litter of four dominant tree species in Mount Tai. Acta Ecologica Sinica. 39(9), 3175-3186. (in Chinese with English abstract)

292 R. Liu, W. Li, W. Yang, B. Tan, W. Wang, Z. Xu, F. Wu. (2013). Contributions of soil fauna to litter decomposition in alpine /subalpine forests. Chinese Journal of Applied Ecology. 24(12), 3354-3360. (in Chinese with English abstract)

293 Y. Meng, L. Zhang, Y. Yuan, X. Jia, H. Cheng, C. Huangfu. (2021). Effects of soil moisture content and litter quality on decomposition of Carex thunbergii fine roots and leaf litter. Research of Environmental Sciences. 34(3), 707-714. (in Chinese with English abstract)

294 S. Zhang, F. Chen, H. Zheng. (2011). Response of soil microbial community structure to the leaf litter decomposition of three typical broadleaf species in mid-subtropical area，southern China. Acta Ecologica Sinica. 31(11), 3020-3026. (in Chinese with English abstract)

295 H. Gao, M. Hong, L. Huo, H. Ye, B. Zhao, H. De. (2018). Effects of exogenous nitrogen input and water change on litter decomposition in a desert grassland. Chinese Journal of Applied Ecology. 29(10), 3167-3174. (in Chinese with English abstract)

296 C. Zhang, H. Zhao, H. Liu, W. Zhang, Y. Su, J. Cheng, W. Yang. (2020). Effects of exogenous nitrogen addition on litter decomposition and nutrient release in a temperate desert. Chinese Journal of Applied Ecology. 31(11), 3631-3638. (in Chinese with English abstract)

297 H. Li, F. Wu, W. Yang, L. Xu, X. Ni, J. He, Y. Hu. (2015). Effects of forest gap on hemicellulose dynamics during foliar litter decomposition in an subalpine forest. Chinese Journal of Plant Ecology. 39(3), 229-238. (in Chinese with English abstract)

298 X. Fan, Y. Ma, J. Feng, Q. Dang, J. Qian, Z. Liu. (2020). Decomposition and nutrient release characteristics of ten common tree leaves in northern cities. Journal of Northwest Forestry University. 35(6), 25-31. (in Chinese with English abstract)

299 L. Yang, C. Deng, Y. Chen, R. He, J. Zhang, Y. Liu. (2015). Relationships between decomposition rate of leaf litter and initial quality across the alpine timberline ecotone in Western Sichuan, China. Chinese Journal of Applied Ecology. 26(12), 3602-3610. (in Chinese with English abstract)

300 P. Wu, K. Lin, N. Xu, L. Shi, M. Chen, W. Zheng, S. Liu. (2017). Effects of litter diversity on Cunninghamia lanceolata litter decomposition. Journal of Southwest Forestry University. 37(2), 103-108. (in Chinese with English abstract)

301 Y. Zhao, Y. Yang, G. Chen, X. Zhong. (2009). Decomposition of leaf litter of Castanopsis carlesii and Cunninghamia lanceolata and their mixtures in Wanmulin Natural Reserve, Fujian Province. Journal of Subtropical Resources and Environment. 4(2), 53-59. (in Chinese with English abstract)

302 Q. Wei, L. Ling, D. Wang, G. Li, J. Qi, C. Chai. (2019). Relationships between decomposition rate of leaf litter and initial quality of main tree species in Xinglong Mountain of Gansu Province. Bulletin of Soil and Water Conservation. 39(1), 9-15+36. (in Chinese with English abstract)

303 J. Lu, T. Bu, B. Guo, P. Zhu, H. Jiang, X. Song. (2013). Effect of UV-B radiation on chemical composition and subsequent decomposition of leaf litter of Cinnamomum camphora. Journal of Zhejiang Forestry Science and Technology. 33(4), 68-73. (in Chinese with English abstract)

304 Q. Wu, F. Wu, B. Tan, W. Yang, W. He, X. Ni. (2016). Effects of gap sizes on foliar litter decomposition in alpine forests. Acta Ecologica Sinica. 36(12), 3537-3545. (in Chinese with English abstract)

305 Y. Yang, J. Guo, P. Lin, G. Chen, Z. He, J. Xie. (2004). Nutrient dynamics of decomposing leaf litter in natural and monoculture plantation forests of Castanopsis kawakamii in subtropical China. Acta Ecologica Sinica. 24(2), 201-208.

306 Z. Xu, X. Wan, Y. Liang, X. Shi. (2021). Effects of root growth on leaf litter decomposition and enzyme activity in litter layer. Chinese Journal of Applied Ecology. 32(1), 31-38. (in Chinese with English abstract)

307 C. Tu, B. Huang, H. Yang, B. Bai, B. Mo, J. Cao. (2018). Study on leaf litter decomposition of Loropetalum chinense and Pinus massoniana in karst areas and clastic rock areas in Maocun, Guilin. Carsologica Sinica. 37(3), 379-387. (in Chinese with English abstract)

308 L. Tu, H. Hu, T. Hu, J. Zhang, S. Luo, H. Dai. (2012). Response of Betula luminifera leaf litter decomposition to simulated nitrogen deposition in the Rainy Area of West China. Chinese Journal of Plant Ecology. 36(2), 99-108. (in Chinese with English abstract)

309 X. Zhang, K. Pan, J. Wang, Q. Chen. (2011). Effects of decomposition of mixed leaf litters of the Castanopsis platyacanthaSchima sinensis forest on soil organic carbon. Acta Ecologica Sinica. 31(6), 1582-1593. (in Chinese with English abstract)

310 Q. Wu, C. Wang. (2018). Dynamics in foliar litter decomposition for Pinus koraiensis and Quercus mongolica in a snow-depth manipulation experiment. Chinese Journal of Plant Ecology. 42(2), 153-163. (in Chinese with English abstract)

311 J. Dou, C. Wang, Q. Wu. (2019). Influence of snow-depth changes on leaf litter decomposition of Fraxinus mandshurica and Larix gmelinii. Chinese Journal of Applied Ecology. 30(1), 77-84. (in Chinese with English abstract)

312 W. Wang, Z. Shi, D. Luo, S. Liu. (2016). Decomposition characteristics of leaf litter and fine roots of Erythrophleum fordii and Castanopsis hystrix in subtropical China. Acta Ecologica Sinica. 36(12), 3479-3487. (in Chinese with English abstract)

313 Y. Wang, X. Rao, S. Tong, X. Han, J. Wang, S. Jian, H. Ren, W. Shen. (2020). Leaf litter decomposition and soil mesofauna contribution in Pisonia grandis and Guettarda speciosa plant communities in the Yongxing Island of South China Sea. Acta Ecologica Sinica. 40(23), 8805-8815. (in Chinese with English abstract)

314 X. Song, T. Pu, S. Zhang, H. Jiang, Z. Wang, M. Zhao, Y. Liu. (2013). Effect of UV-B Radiation on the chemical composition and subsequent decomposition of Cyclobalanopsis glauca leaf litter. Environmental Science. 34(6), 2355-2360. (in Chinese with English abstract)

315 P. Guo, H. Jiang, S. Yu, Y. Ma, R. Dou, X. Song. (2009). Comparison of litter decomposition of six species of coniferous and broad-leaved trees in subtropical China. Chinese Journal of Applied & Environmental Biology. 15(5), 655-659. (in Chinese with English abstract)

316 S. Chen, J. Cai, C. Lin, H. Song, Y. Yang. (2020). Response of leaf litter decomposition of different tree species to nitrogen addition in a subtropical forest. Chinese Journal of Plant Ecology. 44(3), 214-227. (in Chinese with English abstract)

317 X. Song, H. Zhang, H. Jiang, S. Yu. (2012). Effects of UV-B radiation on leaf litter decomposition in humid subtropical region in China. Scientia Silvae Sinicae. 48(1), 13-17. (in Chinese with English abstract)

318 J. Liu, K. Guo, Y. Chen, H. Li, L. Zhang, C. You, Z. Xu, B. Tan. (2021). Changes of soil arthropod community during litter decomposition in a subtropical forest. Acta Ecologica Sinica. 41(7), 2770-2782. (in Chinese with English abstract)

319 B. Guo, T. Bu, Z. Wang, X. Song, G. Liu, S. Fan. (2013). Effects of UV-B radiation on the leaf litter chemistry and subsequent decomposition of Elaeocarpus sylvestris. Chinese Journal of Ecology. 32(9), 2314-2319. (in Chinese with English abstract)

320 M. Zhong, Q. Wang, H. Gao, X. Yu. (2013). Decomposition and nitrogen-and phosphorus release of leaf litters from main tree species in a mid-subtropical forest. Chinese Journal of Ecology. 32(7), 1653-1659. (in Chinese with English abstract)

321 Y. S. Yang, J. F. Guo, G. S. Chen, J. S. Xie, L. P. Cai, P. Lin. (2004). Litterfall, nutrient return, and leaf-litter decomposition in four plantations compared with a natural forest in subtropical China. Annals of Forest Science. 61(5), 465-476.

322 J. Bayala, A. Mando, Z. Teklehaimanot, S. Ouedraogo. (2005). Nutrient release from decomposing leaf mulches of karité (Vitellaria paradoxa) and néré (Parkia biglobosa) under semi-arid conditions in Burkina Faso, West Africa. Soil Biology and Biochemistry. 37(3), 533-539.

323 U. Bjelke, J. Herrmann. (2005). Processing of two detritus types by lake-dwelling shredders: species-specific impacts and effects of species richness. Journal of Animal Ecology. 74, 92-98.

324 M. F. Cotrufo, P. De Angelis, A. Polle. (2005). Leaf litter production and decomposition in a poplar short‐rotation coppice exposed to free air CO2 enrichment (POPFACE). Global Change Biology. 11(6), 971-982.

325 M.-D. Diallo, A. Guisse, A. Badiane-Niane, S. Sall, J.-L. Chotte. (2005). In situ effect of some tropical litters on N mineralization. Arid Land Research and Management. 19(2), 173-181.

326 S. J. Fonte, T. D. Schowalter. (2004). Decomposition of Greenfall vs. Senescent Foliage in a Tropical Forest Ecosystem in Puerto Rico. Biotropica. 36(4), 474-482.

327 P. C. Frost, N. C. Tuchman. (2005). Nutrient release rates and ratios by two stream detritivores fed leaf litter grown under elevated atmospheric CO2. Archiv Fur Hydrobiologie. 163(4), 463-478.

328 M. C. Hall, P. Stiling, B. A. Hungate, B. G. Drake, M. D. Hunter. (2005). Effects of elevated CO2 and herbivore damage on litter quality in a scrub oak ecosystem. Journal of Chemical Ecology. 31(10), 2343-2356.

329 S. E. Hobbie. (2005). Contrasting effects of substrate and fertilizer nitrogen on the early stages of litter decomposition. Ecosystems. 8(6), 644-656.

330 S. R. Isaac, M. A. Nair. (2005). Biodegradation of leaf litter in the warm humid tropics of Kerala, India. Soil Biology and Biochemistry. 37(9), 1656-1664.

331 M. D. Madritch, M. D. Hunter. (2005). Phenotypic variation in oak litter influences short-and long-term nutrient cycling through litter chemistry. Soil Biology and Biochemistry. 37(2), 319-327.

332 R. F. Hughes, A. Uowolo. (2006). Impacts of Falcataria moluccana invasion on decomposition in Hawaiian lowland wet forests: the importance of stand-level controls. Ecosystems. 9(6), 977-991.

333 A. de Neergaard, C. Saarnak, T. Hill, M. Khanyile, A. M. Berzosa, T. Birch-Thomsen. (2005). Australian wattle species in the Drakensberg region of South Africa–An invasive alien or a natural resource? Agricultural systems. 85(3), 216-233.

334 T. Osono, H. Takeda. (2005). Decomposition of organic chemical components in relation to nitrogen dynamics in leaf litter of 14 tree species in a cool temperate forest. Ecological Research. 20(1), 41-49.

335 H. M. Quested, T. V. Callaghan, J. H. C. Cornelissen, M. C. Press. (2005). The impact of hemiparasitic plant litter on decomposition: direct, seasonal and litter mixing effects. Journal of Ecology. 93, 87-98.

336 P. B. Reich, J. Oleksyn, J. Modrzynski, P. Mrozinski, S. E. Hobbie, D. M. Eissenstat, J. Chorover, O. A. Chadwick, C. M. Hale, M. G. Tjoelker. (2005). Linking litter calcium, earthworms and soil properties: a common garden test with 14 tree species. Ecology Letters. 8(8), 811-818.

337 H. Steltzer, W. D. Bowman. (2005). Litter N retention over winter for a low and a high phenolic species in the alpine tundra. Plant and Soil. 275(1), 361-370.

338 X. Xu, E. Hirata. (2005). Decomposition patterns of leaf litter of seven common canopy species in a subtropical forest: N and P dynamics. Plant and Soil. 273(1), 279-289.

339 M. Zimmer, G. Kautz, W. Topp. (2005). Do woodlice and earthworms interact synergistically in leaf litter decomposition? Functional Ecology. 19(1), 7-16.

340 M. Ardon, L. A. Stallcup, C. M. Pringle. (2006). Does leaf quality mediate the stimulation of leaf breakdown by phosphorus in Neotropical streams? Freshwater Biology. 51(4), 618-633.

341 C. Petisco, B. García-Criado, S. Mediavilla, B. R. Vázquez de Aldana, I. Zabalgogeazcoa. (2006). Near-infrared reflectance spectroscopy as a fast and non-destructive tool to predict foliar organic constituents of several woody species. Analytical and Bioanalytical Chemistry. 386(6), 1823-1833.

342 T. B. Gartner, Z. G. Cardon. (2006). Site of leaf origin affects how mixed litter decomposes. Soil Biology and Biochemistry. 38(8), 2307-2317.

343 M. C. Hall, P. Stiling, D. C. Moon, B. G. Drake, M. D. Hunter. (2006). Elevated CO2 increases the long‐term decomposition rate of Quercus myrtifolia leaf litter. Global Change Biology. 12(3), 568-577.

344 S. R. Isaac, M. A. Nair. (2006). Litter dynamics of six multipurpose trees in a homegarden in Southern Kerala, India. Agroforestry systems. 67(3), 203-213.

345 R. Jalota, R. Dalal, B. Harms, K. Page, N. Mathers, W. Wang. (2006). Effects of litter and fine root composition on their decomposition in a Rhodic Paleustalf under different land uses. Communications in Soil Science and Plant Analysis. 37(13-14), 1859-1875.

346 J. Adam Langley, S. K. Chapman, B. A. Hungate. (2006). Ectomycorrhizal colonization slows root decomposition: the post‐mortem fungal legacy. Ecology Letters. 9(8), 955-959.

347 H. A. Leblanc, P. Nygren, R. L. McGraw. (2006). Green mulch decomposition and nitrogen release from leaves of two Inga spp. in an organic alley-cropping practice in the humid tropics. Soil Biology and Biochemistry. 38(2), 349-358.

348 K. Kalbitz, K. Kaiser, J. Bargholz, P. Dardenne. (2006). Lignin degradation controls the production of dissolved organic matter in decomposing foliar litter. European Journal of Soil Science. 57(4), 504-516.

349 T. Osono, S. Hobara, K. Koba, K. Kameda, H. Takeda. (2006). Immobilization of avian excreta-derived nutrients and reduced lignin decomposition in needle and twig litter in a temperate coniferous forest. Soil Biology and Biochemistry. 38(3), 517-525.

350 C. Pernin, J.-P. Ambrosi, J. Cortet, R. Joffre, J. Le Petit, E. Tabone, F. Torre, P. H. Krogh. (2006). Effects of sewage sludge and copper enrichment on both soil mesofauna community and decomposition of oak leaves (Quercus suber) in a mesocosm. Biology and Fertility of Soils. 43(1), 39-50.

351 M. Semmartin, C. M. Ghersa. (2006). Intraspecific changes in plant morphology, associated with grazing, and effects on litter quality, carbon and nutrient dynamics during decomposition. Austral Ecology. 31(1), 99-105.

352 J. Goma-Tchimbakala, F. Bernhard-Reversat. (2006). Comparison of litter dynamics in three plantations of an indigenous timber-tree species (Terminalia superba) and a natural tropical forest in Mayombe, Congo. Forest Ecology and Management. 229(1-3), 304-313.

353 D. N. Vargas, M. B. Bertiller, J. O. Ares, A. L. Carrera, C. L. Sain. (2006). Soil C and N dynamics induced by leaf-litter decomposition of shrubs and perennial grasses of the Patagonian Monte. Soil Biology and Biochemistry. 38(8), 2401-2410.

354 J. Weerakkody, D. Parkinson. (2006). Leaf litter decomposition in an upper montane rainforest in Sri Lanka. Pedobiologia. 50(5), 387-395.

355 E. R. Crawford, F. P. Day, R. B. Atkinson. (2007). Influence of environment and substrate quality on root decomposition in naturally regenerating and restored Atlantic white cedar wetlands. Wetlands. 27(1), 1-11.

356 M. P. Davey, B. Berg, B. A. Emmett, P. Rowland. (2007). Decomposition of oak leaf litter is related to initial litter Mn concentrations. Botany. 85(1), 16-24.

357 T. A. Day, E. T. Zhang, C. T. Ruhland. (2007). Exposure to solar UV-B radiation accelerates mass and lignin loss of Larrea tridentata litter in the Sonoran Desert. Plant Ecology. 193(2), 185-194.

358 A. Sarjubala Devi, P. Yadava. (2007). Wood and leaf litter decomposition of Dipterocarpus tuberculatus Roxb. in a tropical deciduous forest of Manipur, Northeast India. Current Science. 93(2),

359 A. Fioretto, S. Papa, A. Pellegrino, A. Fuggi. (2007). Decomposition dynamics of Myrtus communis and Quercus ilex leaf litter: Mass loss, microbial activity and quality change. Applied Soil Ecology. 36(1), 32-40.

360 A. M. Gama, C. Canhoto. (2007). The breakdown of blue gum (Eucalyptus globulus Labill.) bark in a Portuguese stream. Fundamental and Applied Limnology. 168(4), 307-315.

361 J. E. Hancock, W. M. Loya, C. P. Giardina, L. Li, V. L. Chiang, K. S. Pregitzer. (2007). Plant growth, biomass partitioning and soil carbon formation in response to altered lignin biosynthesis in Populus tremuloides. New Phytologist. 173(4), 732-742.

362 B. R. Rao, T. N. Shivananda, S. Joshi, R. Siddaramappa. (2007). Effects of allelochemicals from selected carbon sources under flooded soil conditions on histological abnormalities induced in rice (Oryza sativa L.) roots. Allelopathy Journal. 20(2), 347.

363 J. S. Kominoski, C. M. Pringle, B. A. Ball, M. Bradford, D. Coleman, D. Hall, M. Hunter. (2007). Nonadditive effects of leaf litter species diversity on breakdown dynamics in a detritus‐based stream. Ecology. 88(5), 1167-1176.

364 G. Loranger-Merciris, D. Imbert, F. Bernhard-Reversat, J.-F. Ponge, P. Lavelle. (2007). Soil fauna abundance and diversity in a secondary semi-evergreen forest in Guadeloupe (Lesser Antilles): influence of soil type and dominant tree species. Biology and Fertility of Soils. 44(2), 269-276.

365 R. Pandey, G. Sharma, S. Tripathi, A. Singh. (2007). Litterfall, litter decomposition and nutrient dynamics in a subtropical natural oak forest and managed plantation in northeastern India. Forest Ecology and Management. 240(1-3), 96-104.

366 U. Puttsepp, K. Lõhmus, A. Koppel. (2007). Decomposition of fine roots and alpha-cellulose in a short rotation willow (Salix spp.) plantation on abandoned agricultural land. Silva Fennica. 41(2), 247.

367 T. Teklay, A. Nordgren, G. Nyberg, A. Malmer. (2007). Carbon mineralization of leaves from four Ethiopian agroforestry species under laboratory and field conditions. Applied Soil Ecology. 35(1), 193-202.

368 T. Teklay. (2007). Decomposition and nutrient release from pruning residues of two indigenous agroforestry species during the wet and dry seasons. Nutrient Cycling in Agroecosystems. 77(2), 115-126.

369 G. Tian, M. Badejo, A. Okoh, F. Ishida, G. Kolawole, Y. Hayashi, F. Salako. (2007). Effects of residue quality and climate on plant residue decomposition and nutrient release along the transect from humid forest to Sahel of West Africa. Biogeochemistry. 86(2), 217-229.

370 S. S. Vasconcelos, D. J. Zarin, M. B. S. Da Rosa, F. de Assis Oliveira, C. J. R. De Carvalho. (2007). Leaf decomposition in a dry season irrigation experiment in Eastern Amazonian forest regrowth. Biotropica. 39(5), 593-600.

371 A. Martinez-Yrizar, S. Nuñez, A. Burquez. (2007). Leaf litter decomposition in a southern Sonoran Desert ecosystem, northwestern Mexico: Effects of habitat and litter quality. Acta Oecologica. 32(3), 291-300.

372 R. J. Andrioli, R. A. Distel. (2008). Litter quality of C3 perennial grasses and soil inorganic nitrogen in a semiarid rangeland of central Argentina. Journal of Arid Environments. 72(9), 1684-1689.

373 B. A. Ball, M. D. Hunter, J. S. Kominoski, C. M. Swan, M. A. Bradford. (2008). Consequences of non‐random species loss for decomposition dynamics: experimental evidence for additive and non‐additive effects. Journal of Ecology. 96(2), 303-313.

374 A. R. Barbhuiya, A. Arunachalam, P. C. Nath, M. L. Khan, K. Arunachalam. (2008). Leaf litter decomposition of dominant tree species of Namdapha National Park, Arunachal Pradesh, northeast India. Journal of Forest Research. 13(1), 25-34.

375 J. G. Cobo, E. Barrios, R. Delve. (2008). Decomposition and nutrient release from intra-specific mixtures of legume plant materials. Communications in Soil Science and Plant Analysis. 39(3-4), 616-625.

376 D. L. Hernández, S. E. Hobbie. (2008). Effects of fire frequency on oak litter decomposition and nitrogen dynamics. Oecologia. 158(3), 535-543.

377 A. R. Holdsworth, L. E. Frelich, P. B. Reich. (2008). Litter decomposition in earthworm-invaded northern hardwood forests: role of invasion degree and litter chemistry. Ecoscience. 15(4), 536-544.

378 T. D. Hooker, J. M. Stark, U. Norton, A. Joshua Leffler, M. Peek, R. Ryel. (2008). Distribution of ecosystem C and N within contrasting vegetation types in a semiarid rangeland in the Great Basin, USA. Biogeochemistry. 90(3), 291-308.

379 B. D. Page, M. J. Mitchell. (2008). Influences of a calcium gradient on soil inorganic nitrogen in the Adirondack Mountains, New York. Ecological Applications. 18(7), 1604-1614.

380 S. A. Parsons, R. A. Congdon. (2008). Plant litter decomposition and nutrient cycling in north Queensland tropical rain-forest communities of differing successional status. Journal of Tropical Ecology. 24(3), 317-327.

381 F. Raimundo, A. Martins, M. Madeira. (2008). Decomposition of chestnut litterfall and eight-year soil chemical changes under a no-tillage management system in Northern Portugal. Annals of Forest Science. 65(4), 1.

382 S. C. Reed, C. C. Cleveland, A. R. Townsend. (2008). Tree species control rates of free‐living nitrogen fixation in a tropical rain forest. Ecology. 89(10), 2924-2934.

383 P. Rovira, C. Kurz-Besson, P. Hernàndez, M.-M. Coûteaux, V. R. Vallejo. (2008). Searching for an indicator of N evolution during organic matter decomposition based on amino acids behaviour: a study on litter layers of pine forests. Plant and Soil. 307(1), 149-166.

384 T. Sariyildiz. (2008). Effects of tree canopy on litter decomposition rates of Abies nordmanniana, Picea orientalis and Pinus sylvestris. Scandinavian Journal of Forest Research. 23(4), 330-338.

385 E. E. Bontti, J. P. Decant, S. M. Munson, M. A. Gathany, A. Przeszlowska, M. L. Haddix, S. Owens, I. C. Burke, W. J. Parton, M. E. Harmon. (2009). Litter decomposition in grasslands of central North America (US Great Plains). Global Change Biology. 15(5), 1356-1363.

386 L. Brandt, C. Bohnet, J. King. (2009). Photochemically induced carbon dioxide production as a mechanism for carbon loss from plant litter in arid ecosystems. Journal of Geophysical Research: Biogeosciences. 114(G2), G02004.

387 S. Diedhiou, E. Dossa, A. Badiane, I. Diedhiou, M. Sene, R. Dick. (2009). Decomposition and spatial microbial heterogeneity associated with native shrubs in soils of agroecosystems in semi-arid Senegal. Pedobiologia. 52(4), 273-286.

388 S. Das, V. C. Joy. (2009). Chemical quality impacts of tropical forest tree leaf litters on the growth and fecundity of soil Collembola. European Journal of Soil Biology. 45(5-6), 448-454.

389 X. He, P. Zhang, Y. Lin, A. Li, X. Tian, Q.-H. Zhang. (2009). Responses of litter decomposition to temperature along a chronosequence of tropical montane rainforest in a microcosm experiment. Ecological Research. 24(4), 781-789.

390 J. Kamei, H. Pandey, S. Barik. (2009). Tree species distribution and its impact on soil properties, and nitrogen and phosphorus mineralization in a humid subtropical forest ecosystem of northeastern India. Canadian Journal of Forest Research. 39(1), 36-47.

391 W. Li, K.-w. Pan, N. Wu, J.-c. Wang, C.-m. Han, X.-l. Liang. (2009). Effects of mixing pine and broadleaved tree/shrub litter on decomposition and N dynamics in laboratory microcosms. Ecological Research. 24(4), 761-769.

392 A. Kubartová, J. Ranger, J. Berthelin, T. Beguiristain. (2009). Diversity and decomposing ability of saprophytic fungi from temperate forest litter. Microbial Ecology. 58(1), 98-107.

393 T. Kunito, Y. Akagi, H.-D. Park, H. Toda. (2009). Influences of nitrogen and phosphorus addition on polyphenol oxidase activity in a forested Andisol. European Journal of Forest Research. 128(4), 361-366.

394 Q. Li, D. L. Moorhead, J. L. DeForest, R. Henderson, J. Chen, R. Jensen. (2009). Mixed litter decomposition in a managed Missouri Ozark forest ecosystem. Forest Ecology and Management. 257(2), 688-694.

395 J. Lindedam, J. Magid, P. Poulsen, J. Luxhøi. (2009). Tissue architecture and soil fertility controls on decomposer communities and decomposition of roots. Soil Biology and Biochemistry. 41(6), 1040-1049.

396 P. Liu, J. Huang, X. Han, O. J. Sun. (2009). Litter decomposition in semiarid grassland of Inner Mongolia, China. Rangeland Ecology & Management. 62(4), 305-313.

397 R. L. McCulley, I. C. Burke, W. K. Lauenroth. (2009). Conservation of nitrogen increases with precipitation across a major grassland gradient in the Central Great Plains of North America. Oecologia. 159(3), 571-581.

398 S. Ndaw, A. Gama-Rodrigues, E. Gama-Rodrigues, K. Sales, A. Rosado. (2009). Relationships between bacterial diversity, microbial biomass, and litter quality in soils under different plant covers in northern Rio de Janeiro State, Brazil. Canadian Journal of Microbiology. 55(9), 1089-1095.

399 K. Piatek, P. Munasinghe, W. Peterjohn, M. Adams, J. Cumming. (2009). Oak contribution to litter nutrient dynamics in an Appalachian forest receiving elevated nitrogen and dolomite. Canadian Journal of Forest Research. 39(5), 936-944.

400 C. Swan, M. Gluth, C. Horne. (2009). Leaf litter species evenness influences nonadditive breakdown in a headwater stream. Ecology. 90(6), 1650-1658.

401 N. C. Tuttle, K. H. Beard, W. C. Pitt. (2009). Invasive litter, not an invasive insectivore, determines invertebrate communities in Hawaiian forests. Biological Invasions. 11(4), 845-855.

402 W. R. Wieder, C. C. Cleveland, A. R. Townsend. (2009). Controls over leaf litter decomposition in wet tropical forests. Ecology. 90(12), 3333-3341.

403 L. Brandt, J. King, S. Hobbie, D. Milchunas, R. Sinsabaugh. (2010). The role of photodegradation in surface litter decomposition across a grassland ecosystem precipitation gradient. Ecosystems. 13(5), 765-781.

404 X. Cheng, Y. Luo, B. Su, X. Zhou, S. Niu, R. Sherry, E. Weng, Q. Zhang. (2010). Experimental warming and clipping altered litter carbon and nitrogen dynamics in a tallgrass prairie. Agriculture, Ecosystems & Environment. 138(3-4), 206-213.

405 I. Dirks, Y. Navon, D. Kanas, R. Dumbur, J. M. Gruenzweig. (2010). Atmospheric water vapor as driver of litter decomposition in Mediterranean shrubland and grassland during rainless seasons. Global Change Biology. 16(10), 2799-2812.

406 C. M. Gray, R. K. Monson, N. Fierer. (2010). Emissions of volatile organic compounds during the decomposition of plant litter. Journal of Geophysical Research: Biogeosciences. 115(G3), G03015.

407 R. Aerts, R. van Logtestijn, P. Karlsson. (2006). Nitrogen supply differentially affects litter decomposition rates and nitrogen dynamics of sub-arctic bog species. Oecologia. 146(4), 652-658.

408 M. Jacob, K. Viedenz, A. Polle, F. M. Thomas. (2010). Leaf litter decomposition in temperate deciduous forest stands with a decreasing fraction of beech (Fagus sylvatica). Oecologia. 164(4), 1083-1094.

409 T. W. Berger, P. Berger. (2014). Does mixing of beech (Fagus sylvatica) and spruce (Picea abies) litter hasten decomposition? Plant and Soil. 377(1), 217-234.

410 J. J. Kelly, A. Bansal, J. Winkelman, L. R. Janus, S. Hell, M. Wencel, P. Belt, K. A. Kuehn, S. T. Rier, N. C. Tuchman. (2010). Alteration of microbial communities colonizing leaf litter in a temperate woodland stream by growth of trees under conditions of elevated atmospheric CO2. Applied and Environmental Microbiology. 76(15), 4950-4959.

411 M. Majumder, A. Shukla, A. Arunachalam. (2010). Nutrient release and fungal succession during decomposition of crop residues in a shifting cultivation system. Communications in Soil Science and Plant Analysis. 41(4), 497-515.

412 T. D. Meehan, M. S. Crossley, R. L. Lindroth. (2010). Impacts of elevated CO2 and O3 on aspen leaf litter chemistry and earthworm and springtail productivity. Soil Biology and Biochemistry. 42(7), 1132-1137.

413 K. B. Piatek, P. Munasinghe, W. T. Peterjohn, M. B. Adams, J. R. Cumming. (2010). A decrease in oak litter mass changes nutrient dynamics in the litter layer of a central hardwood forest. Northern Journal of Applied Forestry. 27(3), 97-104.

414 K. Potthast, U. Hamer, F. Makeschin. (2010). Impact of litter quality on mineralization processes in managed and abandoned pasture soils in Southern Ecuador. Soil Biology and Biochemistry. 42(1), 56-64.

415 F. Song, X. Tian, X. Fan, X. He. (2010). Decomposing ability of filamentous fungi on litter is involved in a subtropical mixed forest. Mycologia. 102(1), 20-26.

416 M. M. Kohmann, J. M. Sanchez, M. L. Silveira, C. B. Brandani, A. Baldo, B. M. Borges, V. S. Ribeirinho, J. M. Vendramini. (2020). Intensification enhances litter carbon and nitrogen decomposition dynamics in subtropical grazinglands. Agrosystems, Geosciences & Environment. 3(1), e20075.

417 M. S. Strickland, J. L. Devore, J. C. Maerz, M. A. Bradford. (2010). Grass invasion of a hardwood forest is associated with declines in belowground carbon pools. Global Change Biology. 16(4), 1338-1350.

418 V. M. Ruíz-Valdiviezo, M. Luna-Guido, A. Galzy, F. A. Gutiérrez-Miceli, L. Dendooven. (2010). Greenhouse gas emissions and C and N mineralization in soils of Chiapas (México) amended with leaves of Jatropha curcas L. Applied Soil Ecology. 46(1), 17-25.

419 E. M. Wolkovich, D. A. Lipson, R. A. Virginia, K. L. Cottingham, D. T. Bolger. (2010). Grass invasion causes rapid increases in ecosystem carbon and nitrogen storage in a semiarid shrubland. Global Change Biology. 16(4), 1351-1365.

420 C. Xiao, I. Janssens, W. Sang, R. Wang, Z. Xie, Z. Pei, Y. Yi. (2010). Belowground carbon pools and dynamics in China's warm temperate and sub-tropical deciduous forests. Biogeosciences. 7(1), 275-287.

421 G. Xu, Y. Hu, S. Wang, Z. Zhang, X. Chang, J. Duan, C. Luo, Z. Chao, A. Su, Q. Lin. (2010). Effects of litter quality and climate change along an elevation gradient on litter mass loss in an alpine meadow ecosystem on the Tibetan plateau. Plant Ecology. 209(2), 257-268.

422 J. Zheng, S. Han, Y. Wang, C. Zhang, M. Li. (2010). Composition and function of microbial communities during the early decomposition stages of foliar litter exposed to elevated CO2 concentrations. European Journal of Soil Science. 61(6), 914-925.

423 D. Baldantoni, M. Fagnano, A. Alfani. (2011). Tropospheric ozone effects on chemical composition and decomposition rate of Quercus ilex L. leaves. Science of the Total Environment. 409(5), 979-984.

424 Y. Carrillo, B. A. Ball, M. A. Bradford, C. F. Jordan, M. Molina. (2011). Soil fauna alter the effects of litter composition on nitrogen cycling in a mineral soil. Soil Biology and Biochemistry. 43(7), 1440-1449.

425 P. Chivenge, B. Vanlauwe, R. Gentile, J. Six. (2011). Organic resource quality influences short-term aggregate dynamics and soil organic carbon and nitrogen accumulation. Soil Biology and Biochemistry. 43(3), 657-666.

426 D. Dieter, D. von Schiller, E. García-Roger, M. Sánchez-Montoya, R. Gómez, J. Mora-Gómez, F. Sangiorgio, J. Gelbrecht, K. Tockner. (2011). Preconditioning effects of intermittent stream flow on leaf litter decomposition. Aquatic Sciences. 73(4), 599-609.

427 L. Galicia, F. Garcia-Oliva. (2011). Litter quality of two remnant tree species affects soil microbial activity in tropical seasonal pastures in Western Mexico. Arid Land Research and Management. 25(1), 75-86.

428 M. Goebel, S. E. Hobbie, B. Bulaj, M. Zadworny, D. D. Archibald, J. Oleksyn, P. B. Reich, D. M. Eissenstat. (2011). Decomposition of the finest root branching orders: linking belowground dynamics to fine‐root function and structure. Ecological Monographs. 81(1), 89-102.

429 A. Kammer, F. Hagedorn. (2011). Mineralisation, leaching and stabilisation of 13 C-labelled leaf and twig litter in a beech forest soil. Biogeosciences. 8(8), 2195-2208.

430 A. O. Akinyele, U. Donald-Amaeshi. (2021). Leaf litter decomposition and nutrient release of three selected agroforestry tree species. Agroforestry Systems. 95(3), 559-570.

431 T. Osono, D. Hirose. (2011). Colonization and lignin decomposition of pine needle litter by Lophodermium pinastri. Forest Pathology. 41(2), 156-162.

432 J. S. Powers, S. Salute. (2011). Macro-and micronutrient effects on decomposition of leaf litter from two tropical tree species: inferences from a short-term laboratory incubation. Plant and Soil. 346(1), 245-257.

433 N. Amougou, I. Bertrand, S. Cadoux, S. Recous. (2012). Miscanthus× giganteus leaf senescence, decomposition and C and N inputs to soil. Global Change Biology Bioenergy. 4(6), 698-707.

434 M. Aulen, B. Shipley, R. Bradley. (2012). Prediction of in situ root decomposition rates in an interspecific context from chemical and morphological traits. Annals of Botany. 109(1), 287-297.

435 D. Balasubramanian, K. Arunachalam, A. Das, A. Arunachalam. (2012). Decomposition and nutrient release of Eichhornia crassipes (Mart.) Solms. under different trophic conditions in wetlands of eastern Himalayan foothills. Ecological Engineering. 44, 111-122.

436 S. R. Bray, K. Kitajima, M. C. Mack. (2012). Temporal dynamics of microbial communities on decomposing leaf litter of 10 plant species in relation to decomposition rate. Soil Biology and Biochemistry. 49, 30-37.

437 K. J. Elgersma, S. Yu, T. Vor, J. G. Ehrenfeld. (2012). Microbial-mediated feedbacks of leaf litter on invasive plant growth and interspecific competition. Plant and Soil. 356(1), 341-355.

438 C. Langenbruch, M. Helfrich, H. Flessa. (2012). Effects of beech (Fagus sylvatica), ash (Fraxinus excelsior) and lime (Tilia spec.) on soil chemical properties in a mixed deciduous forest. Plant and Soil. 352(1), 389-403.

439 J. Sierra, N. Motisi. (2012). Shift in C and N humification during legume litter decomposition in an acid tropical Ferralsol. Soil Research. 50(5), 380-389.

440 M. Pérez-Suárez, J. T. Arredondo-Moreno, E. Huber-Sannwald. (2012). Early stage of single and mixed leaf-litter decomposition in semiarid forest pine-oak: the role of rainfall and microsite. Biogeochemistry. 108(1), 245-258.

441 J. M. Talbot, K. K. Treseder. (2012). Interactions among lignin, cellulose, and nitrogen drive litter chemistry–decay relationships. Ecology. 93(2), 345-354.

442 T. L. Trammell, H. A. Ralston, S. A. Scroggins, M. M. Carreiro. (2012). Foliar production and decomposition rates in urban forests invaded by the exotic invasive shrub, Lonicera maackii. Biological Invasions. 14(3), 529-545.

443 M. D. Wallenstein, M. L. Haddix, D. D. Lee, R. T. Conant, E. A. Paul. (2012). A litter-slurry technique elucidates the key role of enzyme production and microbial dynamics in temperature sensitivity of organic matter decomposition. Soil Biology and Biochemistry. 47, 18-26.

444 M. Zech, R. A. Werner, D. Juchelka, K. Kalbitz, B. Buggle, B. Glaser. (2012). Absence of oxygen isotope fractionation/exchange of (hemi-) cellulose derived sugars during litter decomposition. Organic Geochemistry. 42(12), 1470-1475.

445 J. Zhu, X. He, F. Wu, W. Yang, B. Tan. (2012). Decomposition of Abies faxoniana litter varies with freeze–thaw stages and altitudes in subalpine/alpine forests of southwest China. Scandinavian Journal of Forest Research. 27(6), 586-596.

446 J. S. Cohen, B. Blossey. (2013). No apparent effects of soil inoculum on green frog (Lithobates clamitans Latreille) tadpole performance. Aquatic Ecology. 47(4), 425-431.

447 N. Fanin, S. Barantal, N. Fromin, H. Schimann, P. Schevin, S. Hättenschwiler. (2012). Distinct microbial limitations in litter and underlying soil revealed by carbon and nutrient fertilization in a tropical rainforest. PLoS One. 7(12), e49990.

448 Z. Huang, X. Wan, Z. He, Z. Yu, M. Wang, Z. Hu, Y. Yang. (2013). Soil microbial biomass, community composition and soil nitrogen cycling in relation to tree species in subtropical China. Soil Biology and Biochemistry. 62, 68-75.

449 A. D. Keiser, J. D. Knoepp, M. A. Bradford. (2013). Microbial communities may modify how litter quality affects potential decomposition rates as tree species migrate. Plant and Soil. 372(1), 167-176.

450 Y. Lv, C. Wang, F. Wang, G. Zhao, G. Pu, X. Ma, X. Tian. (2013). Effects of nitrogen addition on litter decomposition, soil microbial biomass, and enzyme activities between leguminous and non-leguminous forests. Ecological Research. 28(5), 793-800.

451 H. G. Ndagurwa, J. S. Dube, D. Mlambo. (2013). The influence of mistletoes on nitrogen cycling in a semi-arid savanna, south-west Zimbabwe. Journal of Tropical Ecology. 29(2), 147-159.

452 I. S. Pearse, R. C. Cobb, R. Karban. (2014). The phenology–substrate‐match hypothesis explains decomposition rates of evergreen and deciduous oak leaves. Journal of Ecology. 102(1), 28-35.

453 X. Song, H. Zhang, H. Jiang, L. A. Donaldson, H. Wang. (2013). Influence of elevated UV-B radiation on leaf litter chemistry and subsequent decomposition in humid subtropical China. Journal of Soils and Sediments. 13(5), 846-853.

454 J. C. Loaiza-Usuga, J. D. León-Peláez, M. I. González-Hernández, J. F. Gallardo-Lancho, W. Osorio-Vega, G. Correa-Londoño. (2013). Alterations in litter decomposition patterns in tropical montane forests of Colombia: a comparison of oak forests and coniferous plantations. Canadian Journal of Forest Research. 43(6), 528-533.

455 M. D. Wallenstein, M. L. Haddix, E. Ayres, H. Steltzer, K. A. Magrini-Bair, E. A. Paul. (2013). Litter chemistry changes more rapidly when decomposed at home but converges during decomposition–transformation. Soil Biology and Biochemistry. 57, 311-319.

456 Q. Wang, F. Xiao, F. Zhang, S. Wang. (2013). Labile soil organic carbon and microbial activity in three subtropical plantations. Forestry. 86(5), 569-574.

457 L. Zhao, Y.-L. Hu, G.-G. Lin, Y.-c. Gao, Y.-T. Fang, D.-H. Zeng. (2013). Mixing effects of understory plant litter on decomposition and nutrient release of tree litter in two plantations in Northeast China. PLoS One. 8(10), e76334.

458 V. X. Apolinário, J. C. Dubeux Jr, A. C. Mello, J. M. Vendramini, M. A. Lira, M. V. Santos, J. P. Muir. (2014). Litter decomposition of signalgrass grazed with different stocking rates and nitrogen fertilizer levels. Agronomy Journal. 106(2), 622-627.

459 M. Bejarano, M. M. Crosby, V. Parra, J. D. Etchevers, J. Campo. (2014). Precipitation regime and nitrogen addition effects on leaf litter decomposition in tropical dry forests. Biotropica. 46(4), 415-424.

460 D. Chaudhary, J. Chikara, A. Ghosh. (2014). Carbon and nitrogen mineralization potential of biofuel crop (Jatropha curcas L.) residues in soil. Journal of Soil Science and Plant Nutrition. 14(1), 15-30.

461 B. R. Clarkson, T. R. Moore, N. B. Fitzgerald, D. Thornburrow, C. H. Watts, S. Miller. (2014). Water table regime regulates litter decomposition in restiad peatlands, New Zealand. Ecosystems. 17(2), 317-326.

462 C. Furey, P. A. Tecco, N. Perez-Harguindeguy, M. A. Giorgis, M. Grossi. (2014). The importance of native and exotic plant identity and dominance on decomposition patterns in mountain woodlands of central Argentina. Acta Oecologica. 54, 13-20.

463 W. Fuzhong, P. Changhui, Z. Jianxiao, Z. Jian, T. Bo, Y. Wanqin. (2014). Impacts of freezing and thawing dynamics on foliar litter carbon release in alpine/subalpine forests along an altitudinal gradient in the eastern Tibetan Plateau. Biogeosciences. 11(22), 6471-6481.

464 G. S. Guendehou, J. Liski, M. Tuomi, M. Moudachirou, B. Sinsin, R. Makipaa. (2014). Decomposition and changes in chemical composition of leaf litter of five dominant tree species in a West African tropical forest. Tropical Ecology. 55(2), 207-220.

465 C. Herzog, J. Steffen, E. Graf Pannatier, I. Hajdas, I. Brunner. (2014). Nine years of irrigation cause vegetation and fine root shifts in a water-limited pine forest. PLoS One. 9(5), e96321.

466 S. E. Hobbie, L. A. Baker, C. Buyarski, D. Nidzgorski, J. C. Finlay. (2014). Decomposition of tree leaf litter on pavement: implications for urban water quality. Urban Ecosystems. 17(2), 369-385.

467 O. Kara, I. Bolat, K. Cakıroglu, M. Senturk. (2014). Litter decomposition and microbial biomass in temperate forests in Northwestern Turkey. Journal of Soil Science and Plant Nutrition. 14(1), 31-41.

468 C. Langenbruch, M. Helfrich, R. G. Joergensen, J. Gordon, H. Flessa. (2014). Partitioning of carbon and nitrogen during decomposition of 13C15N‐labeled beech and ash leaf litter. Journal of Plant Nutrition and Soil Science. 177(2), 178-188.

469 M. L. Moreno, M. L. Bernaschini, N. Pérez-Harguindeguy, G. Valladares. (2014). Area and edge effects on leaf-litter decomposition in a fragmented subtropical dry forest. Acta Oecologica. 60, 26-29.

470 S. Mukhopadhyay, S. Roy, V. Joy. (2014). Enhancement of soil enzyme activities by the feeding impact of detritivore arthropods on tropical forest tree leaf litters. Tropical Ecology. 55(1), 93-108.

471 S. Prokushkin, A. Prokushkin, N. Sorokin. (2014). The intensity of phytodetrite decomposition in larch forest of the permafrost zone in Central Siberia. Biology Bulletin. 41(1), 89-97.

472 J. Ramírez, J. León-Peláez, D. Craven, D. Herrera, C. Zapata, M. González-Hernández, J. Gallardo-Lancho, W. Osorio. (2014). Effects on nutrient cycling of conifer restoration in a degraded tropical montane forest. Plant and Soil. 378(1), 215-226.

473 T. L. Sausen, G. F. d. P. Schaefer, M. Tomazi, L. S. d. Santos, C. Bayer, L. M. G. Rosa. (2014). Clay content drives carbon stocks in soils under a plantation of Eucalyptus saligna Labill. in southern Brazil. Acta Botanica Brasilica. 28(2), 266-273.

474 X. Song, H. Zhang, H. Jiang, C. Peng. (2014). Combination of nitrogen deposition and ultraviolet-B radiation decreased litter decomposition in subtropical China. Plant and Soil. 380(1), 349-359.

475 L.-h. Tu, H.-l. Hu, G. Chen, Y. Peng, Y.-l. Xiao, T.-x. Hu, J. Zhang, X.-w. Li, L. Liu, Y. Tang. (2014). Nitrogen addition significantly affects forest litter decomposition under high levels of ambient nitrogen deposition. PLoS One. 9(2), e88752.

476 Q. Wu, F. Wu, W. Yang, Y. Zhao, W. He, B. Tan. (2014). Foliar litter nitrogen dynamics as affected by forest gap in the alpine forest of Eastern Tibet Plateau. PLoS One. 9(5), e97112.

477 W. Xiao, X. Ge, L. Zeng, Z. Huang, J. Lei, B. Zhou, M. Li. (2014). Rates of litter decomposition and soil respiration in relation to soil temperature and water in different-aged Pinus massoniana forests in the three gorges reservoir area, China. PLoS One. 9(7), e101890.

478 J. Zheng, Z. Xu, Y. Wang, H. Dong, C. Chen, S. Han. (2014). Non-additive effects of mixing different sources of dissolved organic matter on its biodegradation. Soil Biology and Biochemistry. 78, 160-169.

479 K. Yang, J.-J. Zhu. (2015). Impact of tree litter decomposition on soil biochemical properties obtained from a temperate secondary forest in Northeast China. Journal of Soils and Sediments. 15(1), 13-23.

480 M. Kaleeem Abbasi, M. Mahmood Tahir, N. Sabir, M. Khurshid. (2015). Impact of the addition of different plant residues on nitrogen mineralization–immobilization turnover and carbon content of a soil incubated under laboratory conditions. Solid Earth. 6(1), 197-205.

481 Y. Chen, J. Sun, F. Xie, X. Wang, G. Cheng, X. Lu. (2015). Litter chemical structure is more important than species richness in affecting soil carbon and nitrogen dynamics including gas emissions from an alpine soil. Biology and Fertility of Soils. 51(7), 791-800.

482 M. Coulis, N. Fromin, J. F. David, J. Gavinet, A. Clet, S. Devidal, J. Roy, S. Hättenschwiler. (2015). Functional dissimilarity across trophic levels as a driver of soil processes in a Mediterranean decomposer system exposed to two moisture levels. Oikos. 124(10), 1304-1316.

483 C. A. Creamer, A. B. de Menezes, E. S. Krull, J. Sanderman, R. Newton-Walters, M. Farrell. (2015). Microbial community structure mediates response of soil C decomposition to litter addition and warming. Soil Biology and Biochemistry. 80, 175-188.

484 T. A. Day, R. Guénon, C. T. Ruhland. (2015). Photodegradation of plant litter in the Sonoran Desert varies by litter type and age. Soil Biology and Biochemistry. 89, 109-122.

485 W. He, F. Wu, D. Zhang, W. Yang, B. Tan, Y. Zhao, Q. Wu. (2015). The effects of forest gaps on cellulose degradation in the foliar litter of two shrub species in an alpine fir forest. Plant and Soil. 393(1), 109-122.

486 S. Liao, W. Yang, Y. Tan, Y. Peng, J. Li, B. Tan, F. Wu. (2015). Soil fauna affects dissolved carbon and nitrogen in foliar litter in alpine forest and alpine meadow. PLoS One. 10(9), e0139099.

487 P. Lucisine, A. Lecerf, M. Danger, V. Felten, D. Aran, A. Auclerc, E. M. Gross, H. Huot, J.-L. Morel, S. Muller. (2015). Litter chemistry prevails over litter consumers in mediating effects of past steel industry activities on leaf litter decomposition. Science of the Total Environment. 537, 213-224.

488 N. Makita, S. Fujii. (2015). Tree species effects on microbial respiration from decomposing leaf and fine root litter. Soil Biology and Biochemistry. 88, 39-47.

489 B. Mao, Z.-Y. Yu, D.-H. Zeng. (2015). Non-additive effects of species mixing on litter mass loss and chemical properties in a Mongolian pine plantation of Northeast China. Plant and Soil. 396(1), 339-351.

490 E. Paudel, G. G. Dossa, M. de Blécourt, P. Beckschäfer, J. Xu, R. D. Harrison. (2015). Quantifying the factors affecting leaf litter decomposition across a tropical forest disturbance gradient. Ecosphere. 6(12), 1-20.

491 H. Zhao, G. Huang, Y. Li, J. Ma, J. Sheng, H. Jia, C. Li. (2015). Effects of increased summer precipitation and nitrogen addition on root decomposition in a temperate desert. PLoS One. 10(11), e0142380.

492 C. Biasi, C. Cerezer, S. Santos. (2016). Biological colonization and leaf decomposition in a subtropical stream. Ecología Austral. 26(2), 189-199.

493 S. Biswas, A. Ghosh, A. K. Sinha, P. Mukhopadhyay. (2016). Patterns of nitrogen mineralization under two water regimes in an acidic entisol as influenced by chemical composition of plant residues. Communications in Soil Science and Plant Analysis. 47(7), 851-862.

494 Y. Chen, E. J. Sayer, Z. Li, Q. Mo, Y. Li, Y. Ding, J. Wang, X. Lu, J. Tang, F. Wang. (2016). Nutrient limitation of woody debris decomposition in a tropical forest: contrasting effects of N and P addition. Functional Ecology. 30(2), 295-304.

495 C. Das, N. K. Mondal. (2016). Litterfall, decomposition and nutrient release of Shorea robusta and Tectona grandis in a sub-tropical forest of West Bengal, Eastern India. Journal of Forestry Research. 27(5), 1055-1065.

496 N. Fanin, S. Hättenschwiler, P. F. Chavez Soria, N. Fromin. (2016). (A) synchronous availabilities of N and P regulate the activity and structure of the microbial decomposer community. Frontiers in Microbiology. 6, 1507.

497 V. Ferreira, M. A. Graca. (2016). Effects of whole-stream nitrogen enrichment and litter species mixing on litter decomposition and associated fungi. Limnologica. 58, 69-77.

498 S. Fujii, N. Makita, A. S. Mori, H. Takeda. (2016). A stronger coordination of litter decomposability between leaves and fine roots for woody species in a warmer region. Trees. 30(2), 395-404.

499 J. Gao, F. Kang, H. Han. (2016). Effect of Litter Quality on Leaf-Litter Decomposition in the Context of Home-Field Advantage and Non-Additive Effects in Temperate Forests in China. Polish Journal of Environmental Studies. 25(5),

500 P. P. Gomes, A. O. Medeiros, J. F. G. Júnior. (2016). The replacement of native plants by exotic species may affect the colonization and reproduction of aquatic hyphomycetes. Limnologica. 59, 124-130.

501 W. He, F. Wu, W. Yang, B. Tan, Y. Zhao, Q. Wu, M. He. (2016). Lignin degradation in foliar litter of two shrub species from the gap center to the closed canopy in an alpine fir forest. Ecosystems. 19(1), 115-128.

502 M. Kasahara, S. Fujii, T. Tanikawa, A. S. Mori. (2016). Ungulates decelerate litter decomposition by altering litter quality above and below ground. European Journal of Forest Research. 135(5), 849-856.

503 Q. Li, G. Zhao, G. Cao, Z. Liu. (2016). Soil effects of six different two-species litter mixtures that include Ulmus pumila. Chemistry and Ecology. 32(8), 707-721.

504 H. Li, F. Wu, W. Yang, L. Xu, X. Ni, J. He, B. Tan, Y. Hu, M. F. Justin. (2016). The losses of condensed tannins in six foliar litters vary with gap position and season in an alpine forest. iForest-Biogeosciences and Forestry. 9(6), 910.

505 L. Zengqiang, Z. Bingzi, J. ZHANG. (2016). Effects of maize residue quality and soil water content on soil labile organic carbon fractions and microbial properties. Pedosphere. 26(6), 829-838.

506 T. Ohta, S. Matsunaga, S. Niwa, K. Kawamura, T. Hiura. (2016). Detritivore stoichiometric diversity alters litter processing efficiency in a freshwater ecosystem. Oikos. 125(8), 1162-1172.

507 H. C. A. Pinto, P. A. B. Barreto, E. F. d. Gama-Rodrigues, F. G. R. Oliveira, A. d. Paula, A. R. Amaral. (2016). Decomposição da serapilheira foliar de floresta nativa e plantios de Pterogyne nitens e Eucalyptus urophylla no Sudoeste da Bahia. Ciência Florestal. 26, 1141-1153.

508 A. Rai, A. K. Singh, N. Ghosal, N. Singh. (2016). Understanding the effectiveness of litter from tropical dry forests for the restoration of degraded lands. Ecological Engineering. 93, 76-81.

509 L. A. Leite-Rossi, V. S. Saito, M. B. Cunha-Santino, S. Trivinho-Strixino. (2016). How does leaf litter chemistry influence its decomposition and colonization by shredder Chironomidae (Diptera) larvae in a tropical stream? Hydrobiologia. 771(1), 119-130.

510 E. M. Schilling, B. G. Waring, J. S. Schilling, J. S. Powers. (2016). Forest composition modifies litter dynamics and decomposition in regenerating tropical dry forest. Oecologia. 182(1), 287-297.

511 Q.-n. Song, M. Ouyang, Q.-p. Yang, H. Lu, G.-y. Yang, F.-s. Chen, J.-M. Shi. (2016). Degradation of litter quality and decline of soil nitrogen mineralization after moso bamboo (Phyllostachys pubscens) expansion to neighboring broadleaved forest in subtropical China. Plant and Soil. 404(1), 113-124.

512 T. Sun, L. Dong, Z. Wang, X. Lü, Z. Mao. (2016). Effects of long-term nitrogen deposition on fine root decomposition and its extracellular enzyme activities in temperate forests. Soil Biology and Biochemistry. 93, 50-59.

513 A. Versini, J.-P. Laclau, L. Mareschal, C. Plassard, L. A. Diamesso, J. Ranger, B. Zeller. (2016). Nitrogen dynamics within and between decomposing leaves, bark and branches in Eucalyptus planted forests. Soil Biology and Biochemistry. 101, 55-64.

514 X. Yajun, X. Yonghong, C. Xinsheng, L. Feng, H. Zhiyong, L. Xu. (2016). Non-additive effects of water availability and litter quality on decomposition of litter mixtures. Journal of Freshwater Ecology. 31(2), 153-168.

515 Z. Xu, J. Zhu, F. Wu, Y. Liu, B. Tan, W. Yang. (2016). Effects of litter quality and climate change along an elevational gradient on litter decomposition of subalpine forests, Eastern Tibetan Plateau, China. Journal of Forestry Research. 27(3), 505-511.

516 K. Yue, F. Wu, W. Yang, C. Zhang, Y. Peng, B. Tan, Z. Xu, C. Huang. (2016). Cellulose dynamics during foliar litter decomposition in an alpine forest meta-ecosystem. Forests. 7(8), 176.

517 G. Pei, J. Liu, B. Peng, C. Wang, P. Jiang, E. Bai. (2020). Nonlinear coupling of carbon and nitrogen release during litter decomposition and its responses to nitrogen addition. Journal of Geophysical Research: Biogeosciences. 125(5), e2019JG005462.

518 C. Jiang, W. Yu. (2019). Combined influence of external nitrogen and soil contact on plant residue decomposition and indications from stable isotope signatures. Environmental Science and Pollution Research. 26(7), 6791-6800.

519 H. Jing, P. Zhang, J. Li, X. Yao, G. Liu, G. Wang. (2019). Effect of nitrogen addition on the decomposition and release of compounds from fine roots with different diameters: the importance of initial substrate chemistry. Plant and Soil. 438(1), 281-296.

520 G. Morozov, J. Aosaar, M. Varik, H. Becker, K. Lõhmus, A. Padari, K. Aun, V. Uri. (2019). Long-term dynamics of leaf and root decomposition and nitrogen release in a grey alder (Alnus incana (L.) Moench) and silver birch (Betula pendula Roth.) stands. Scandinavian Journal of Forest Research. 34(1), 12-25.

521 S. T. Partey, R. B. Zougmore, N. V. Thevathasan, R. F. Preziosi. (2019). Effects of plant residue decomposition on soil n availability, microbial biomass and β-glucosidase activity during soil fertility improvement in Ghana. Pedosphere. 29(5), 608-618.

522 L. H. Pazianoto, A. Solla, V. Ferreira. (2019). Leaf litter decomposition of sweet chestnut is affected more by oomycte infection of trees than by water temperature. Fungal Ecology. 41, 269-278.

523 B. Pey, C. Trân, P. Cruz, M. Hedde, C. Jouany, C. Laplanche, J. Nahmani, E. Chauvet, A. Lecerf. (2019). Nutritive value and physical and chemical deterrents of forage grass litter explain feeding performances of two soil macrodetritivores. Applied Soil Ecology. 133, 81-88.

524 I. Prieto, M. Almagro, F. Bastida, J. I. Querejeta. (2019). Altered leaf litter quality exacerbates the negative impact of climate change on decomposition. Journal of Ecology. 107(5), 2364-2382.

525 C. T. Ruhland, J. A. Niere. (2019). The effects of surface albedo and initial lignin concentration on photodegradation of two varieties of Sorghum bicolor litter. Scientific Reports. 9(1), 1-9.

526 A. Aupic-Samain, V. Baldy, C. Lecareux, C. Fernandez, M. Santonja. (2019). Tree litter identity and predator density control prey and predator demographic parameters in a Mediterranean litter-based multi-trophic system. Pedobiologia. 73, 1-9.

527 H.-H. Song, T. Yan, D.-H. Zeng. (2019). Establishment of mixed plantations of Pinus sylvestris var. mongolica and Populus× xiaozhuanica may not be appropriate: evidence from litter decomposition. Journal of Plant Ecology. 12(5), 857-870.

528 T. Sun, Y. Cui, B. Berg, Q. Zhang, L. Dong, Z. Wu, L. Zhang. (2019). A test of manganese effects on decomposition in forest and cropland sites. Soil Biology and Biochemistry. 129, 178-183.

529 Y. Tan, W. Yang, X. Ni, B. Tan, K. Yue, R. Cao, S. Liao, F. Wu. (2019). Soil fauna affects the optical properties in alkaline solutions extracted (humic acid-like) from forest litters during different phenological periods. Canadian Journal of Soil Science. 99(2), 195-207.

530 K. Tian, X. Kong, L. Yuan, H. Lin, Z. He, B. Yao, Y. Ji, J. Yang, S. Sun, X. Tian. (2019). Priming effect of litter mineralization: the role of root exudate depends on its interactions with litter quality and soil condition. Plant and Soil. 440(1), 457-471.

531 K. Upadhyaya, U. Sahoo, K. Vanlalhriatpuia, S. Roy. (2012). Decomposition dynamics and nutrient release pattern from leaf litters of five commonly occurring homegarden tree species in Mizoram, India. Journal of sustainable forestry. 31(8), 711-726.

532 E. Remy, K. Wuyts, L. Van Nevel, P. De Smedt, P. Boeckx, K. Verheyen. (2018). Driving factors behind litter decomposition and nutrient release at temperate forest edges. Ecosystems. 21(4), 755-771.

533 A. Alonso, N. González-Muñoz, P. Castro-Díez. (2010). Comparison of leaf decomposition and macroinvertebrate colonization between exotic and native trees in a freshwater ecosystem. Ecological Research. 25(3), 647-653.

534 A. Negussie, J. Degerickx, L. Norgrove, W. M. Achten, K. M. Hadgu, E. Aynekulu, B. Muys. (2015). Effects of accession, spacing and pruning management on in-situ leaf litter decomposition of Jatropha curcas L. in Zambia. Biomass and Bioenergy. 81, 505-513.

535 H. D. Alexander, M. A. Arthur. (2014). Increasing red maple leaf litter alters decomposition rates and nitrogen cycling in historically oak-dominated forests of the eastern US. Ecosystems. 17(8), 1371-1383.

536 M. Almagro, J. Martínez-López, F. T. Maestre, A. Rey. (2017). The contribution of photodegradation to litter decomposition in semiarid Mediterranean grasslands depends on its interaction with local humidity conditions, litter quality and position. Ecosystems. 20(3), 527-542.

537 M. L. A. Sayao. (2006). Effects of leaf litter quality on decomposition dynamics in lowland Neotropical streams. University of Georgia.

538 J. B. Yavitt, C. J. Williams. (2015). Linking tree species identity to anaerobic microbial activity in a forested wetland soil via leaf litter decomposition and leaf carbon fractions. Plant and Soil. 390(1), 293-305.

539 M. A. Arthur, S. R. Bray, C. R. Kuchle, R. W. McEwan. (2012). The influence of the invasive shrub, Lonicera maackii, on leaf decomposition and microbial community dynamics. Plant Ecology. 213(10), 1571-1582.

540 B. R. Taylor, D. Parkinson. (1988). Aspen and pine leaf litter decomposition in laboratory microcosms. II. Interactions of temperature and moisture level. Canadian Journal of Botany. 66(10), 1966-1973.

541 B. Berg, G. Ekbohm. (1991). Litter mass-loss rates and decomposition patterns in some needle and leaf litter types. Long-term decomposition in a Scots pine forest. VII. Canadian Journal of Botany. 69(7), 1449-1456.

542 B. Berg, M.-B. Johansson, R. C. d. Anta, A. Escudero, A. Gärdenäs, R. Laskowski, M. Madeira, E. Mälkönen, C. McClaugherty, V. Meentemeyer. (1995). The chemical composition of newly shed needle litter of Scots pine and some other pine species in a climatic transect. X Long-term decomposition in a Scots pine forest. Canadian Journal of Botany. 73(9), 1423-1435.

543 A. Balibrea, V. Ferreira, C. Balibrea, V. Gonçalves, P. M. Raposeiro. (2020). Contribution of macroinvertebrate shredders and aquatic hyphomycetes to litter decomposition in remote insular streams. Hydrobiologia. 847(10), 2337-2355.

544 J. M. Blair, D. Crossley Jr. (1988). Litter decomposition, nitrogen dynamics and litter microarthropods in a southern Appalachian hardwood forest 8 years following clearcutting. Journal of Applied Ecology. 25(2), 683-698.

545 G. Bonanomi, G. Cesarano, S. A. Gaglione, F. Ippolito, T. Sarker, M. A. Rao. (2017). Soil fertility promotes decomposition rate of nutrient poor, but not nutrient rich litter through nitrogen transfer. Plant and Soil. 412(1), 397-411.

546 L. Bragazza, P. Iacumin. (2009). Seasonal variation in carbon isotopic composition of bog plant litter during 3 years of field decomposition. Biology and Fertility of Soils. 46(1), 73-77.

547 L. A. Brandt. (2009). The role of photodegradation in plant litter decomposition in grassland ecosystems. University of Minnesota.

548 M. Cakır, E. Makineci. (2020). Litter decomposition in pure and mixed Quercus and Fagus stands as influenced by arthropods in Belgrad Forest, Turkey. Journal of Forestry Research. 31(4), 1123-1137.

549 B. Cassart, A. Angbonga Basia, M. Jonard, Q. Ponette. (2020). Average leaf litter quality drives the decomposition of single-species, mixed-species and transplanted leaf litters for two contrasting tropical forest types in the Congo Basin (DRC). Annals of Forest Science. 77(2), 1-20.

550 P. Castro-Díez, Á. Alonso, A. Romero-Blanco. (2019). Effects of litter mixing on litter decomposition and soil properties along simulated invasion gradients of non-native trees. Plant and Soil. 442(1), 79-96.

551 S. K. Chapman, G. W. Koch. (2007). What type of diversity yields synergy during mixed litter decomposition in a natural forest ecosystem? Plant and Soil. 299(1), 153-162.

552 X. Chuan, C. N. Carlyle, E. W. Bork, S. X. Chang, D. B. Hewins. (2018). Long-term grazing accelerated litter decomposition in northern temperate grasslands. Ecosystems. 21(7), 1321-1334.

553 S. Pereira, A. Burešová, J. Kopecky, P. Mádrová, A. Aupic-Samain, C. Fernandez, V. Baldy, M. Sagova-Mareckova. (2019). Litter traits and rainfall reduction alter microbial litter decomposers: the evidence from three Mediterranean forests. FEMS Microbiology Ecology. 95(12), fiz168.

554 L. Cizungu, J. Staelens, D. Huygens, J. Walangululu, D. Muhindo, O. Van Cleemput, P. Boeckx. (2014). Litterfall and leaf litter decomposition in a central African tropical mountain forest and Eucalyptus plantation. Forest Ecology and Management. 326, 109-116.

555 G. Yan, X. Dong, B. Huang, H. Wang, Z. Hong, J. Zhang, Y. Xing, Q. Wang. (2019). Effects of nitrogen deposition on litter decomposition and nutrient release mediated by litter types and seasonal change in a temperate forest. Canadian Journal of Soil Science. 100(1), 11-25.

556 M. Santonja, C. Fernandez, T. Gauquelin, V. Baldy. (2015). Climate change effects on litter decomposition: intensive drought leads to a strong decrease of litter mixture interactions. Plant and Soil. 393(1), 69-82.

557 E. Correa, L. Carvalhais, M. Utida, C. A. Oliveira, M. R. Scotti. (2015). Effect of plant species on P cycle-related microorganisms associated with litter decomposition and P soil availability: implications for agroforestry management. iForest-Biogeosciences and Forestry. 9(2), 294.

558 M. Coulis, S. Hättenschwiler, N. Fromin, J. David. (2013). Macroarthropod-microorganism interactions during the decomposition of Mediterranean shrub litter at different moisture levels. Soil Biology and Biochemistry. 64, 114-121.

559 E. Sheffer, C. D. Canham, J. Kigel, A. Perevolotsky. (2015). Countervailing effects on pine and oak leaf litter decomposition in human-altered Mediterranean ecosystems. Oecologia. 177(4), 1039-1051.

560 K. E. Lee, S. Cha, S. H. Lee, J. K. Shim. (2015). Decomposition of leaf litter of some evergreen broadleaf trees in Korea. Journal of Ecology and Environment. 38(4), 517-528.

561 S. Medina Villar, Á. Alonso Fernández, B. R. Vazquez de Aldana, M. E. Pérez Corona, M. P. Castro Díez. (2015). Decomposition and biological colonization of native and exotic leaf litter in a stream. Limnetica. 34(2), 293-310.

562 T. Lehto, A. Smolander, P. J. Aphalo. (2010). Decomposition and element concentrations of silver birch leaf litter as affected by boron status of litter and soil. Plant and Soil. 329(1), 195-208.

563 A. De Marco, R. Spaccini, A. Virzo De Santo. (2021). Differences in nutrients, organic components and decomposition pattern of Phillyrea angustifolia leaf litter across a low maquis. Plant and Soil. 464(1), 559-578.

564 X.-w. Deng, Y. Liu, S.-j. Han. (2009). Carbon and nitrogen dynamics in early stages of forest litter decomposition as affected by nitrogen addition. Journal of Forestry Research. 20(2), 111-116.

565 G. G. Dossa, E. Paudel, D. Schaefer, J.-L. Zhang, K.-F. Cao, J.-C. Xu, R. D. Harrison. (2020). Quantifying the factors affecting wood decomposition across a tropical forest disturbance gradient. Forest Ecology and Management. 468, 118166.

566 H. Wang, S. Zhuang, Z. Qian, J. Cao. (2019). Leaf litter decomposition dynamics of Pinus taiwanensis and Quercus variabilis associated with various elevations. Journal of Research in Ecology 7(1), 2486-2496.

567 N. Du, W. Li, L. Qiu, Y. Zhang, X. Wei, X. Zhang. (2020). Mass loss and nutrient release during the decomposition of sixteen types of plant litter with contrasting quality under three precipitation regimes. Ecology and Evolution. 10(7), 3367-3382.

568 F. Wang, D. Lin, W. Li, P. Dou, L. Han, M. Huang, S. Qian, J. Yao. (2020). Meiofauna promotes litter decomposition in stream ecosystems depending on leaf species. Ecology and Evolution. 10(17), 9257-9270.

569 J. R. McLaren, K. M. Buckeridge, M. J. van de Weg, G. R. Shaver, J. P. Schimel, L. Gough. (2017). Shrub encroachment in Arctic tundra: Betula nana effects on above‐and belowground litter decomposition. Ecology. 98(5), 1361-1376.

570 Y. Liu, J. Ma, Z. Bu, S. Wang, X. Zhang, T. Zhang, S. Liu, B. Fu, Y. Kang. (2018). Effect of geographical sources and biochemical traits on plant litter decomposition in a peatland. Chinese Journal of Plant Ecology. 42(7), 713-722. (in Chinese with English abstract)

571 C. Wang, P. Guo, G. Han, X. Feng, P. Zhang, X. Tian. (2010). Effect of simulated acid rain on the litter decomposition of Quercus acutissima and Pinus massoniana in forest soil microcosms and the relationship with soil enzyme activities. Science of the Total Environment. 408(13), 2706-2713.

572 C. Guo, W. Yang, F. Wu, Z. Xu, K. Yue, X. Ni, J. Yuan, F. Yang, B. Tan. (2018). Effects of forest gap size on initial decomposition of twig litter in the subalpine forest of western Sichuan, China. Chinese Journal of Plant Ecology. 42(1), 28-37. (in Chinese with English abstract)

573 S.-M. Lim, S.-S. Cha, J.-K. Shim. (2011). Effects of simulated acid rain on microbial activities and litter decomposition. Journal of Ecology and Environment. 34(4), 401-410.

574 E. F. Salamanca, N. Kaneko, S. Katagiri. (1998). Effects of leaf litter mixtures on the decomposition of Quercus serrata and Pinus densiflora using field and laboratory microcosm methods. Ecological Engineering. 10(1), 53-73.

575 L. Wang, J. Zhang, R. He, Y. Chen, L. Yang, H. Zheng, H. Li, J. Xiao, Y. Liu. (2018). Impacts of soil fauna on lignin and cellulose degradation in litter decomposition across an alpine forest-tundra ecotone. European Journal of Soil Biology. 87, 53-60.

576 S. S. Perakis, J. J. Matkins, D. E. Hibbs. (2012). Interactions of tissue and fertilizer nitrogen on decomposition dynamics of lignin‐rich conifer litter. Ecosphere. 3(6), 1-12.

577 U. Pandey, J. Singh. (1982). Leaf-litter decomposition in an oak-conifer forest in Himalaya: the effects of climate and chemical composition. Forestry: An International Journal of Forest Research. 55(1), 47-59.

578 J. Gao, F. Kang, T. Li, X. Song, W. Zhao, X. Yu, H. Han. (2015). Assessing the effect of leaf litter diversity on the decomposition and associated diversity of fungal assemblages. Forests. 6(7), 2371-2386.

579 H. M. Chae, S. H. Choi, S. H. Lee, S. Cha, K. C. Yang, J. K. Shim. (2019). Effect of litter quality on needle decomposition for four pine species in Korea. Forests. 10(5), 371.

580 J. Frouz, P. Jedlička, H. Šimáčková, Z. Lhotáková. (2015). The life cycle, population dynamics, and contribution to litter decomposition of Penthetria holosericea (Diptera: Bibionidae) in an alder forest. European Journal of Soil Biology. 71, 21-27.

581 V. Ferreira, P. M. Raposeiro, A. Pereira, A. M. Cruz, A. C. Costa, M. A. Graça, V. Gonçalves. (2016). Leaf litter decomposition in remote oceanic island streams is driven by microbes and depends on litter quality and environmental conditions. Freshwater Biology. 61(5), 783-799.

582 A. d. Gama-Rodrigues, N. F. Barros, M. L. Santos. (2003). Decomposição e liberação de nutrientes do folhedo de espécies florestais nativas em plantios puros e mistos no sudeste da Bahia. Revista Brasileira de Ciência do Solo. 27, 1021-1031.

583 J. Gao, H. Han, F. Kang. (2019). Factors Controlling Decomposition Rates of Needle Litter Across a Chronosequence of Chinese Pine (Pinus tabuliformis Carr.) Forests. Polish Journal of Environmental Studies. 28(1),

584 J. Gong, C. Zhu, L. Yang, B. Yang, B. Wang, T.-t. Baoyin, M. Liu, Z. Zhang, J. Shi. (2020). Effects of nitrogen addition on above-and belowground litter decomposition and nutrient dynamics in the litter-soil continuum in the temperate steppe of Inner Mongolia, China. Journal of Arid Environments. 172, 104036.

585 Z. R. Guerrero. (2015). THESIS SIGNATURE PAGE. California State University San Marcos.

586 R. Guo, J. Zheng, S. Han, J. Zhang, M.-H. Li. (2013). Carbon and nitrogen turnover in response to warming and nitrogen addition during early stages of forest litter decomposition—an incubation experiment. Journal of Soils and Sediments. 13(2), 312-324.

587 W. He, L. Lei, Z. Ma, M. Teng, P. Wang, Z. Yan, Z. Huang, L. Zeng, W. Xiao. (2020). Nonadditive effects of decomposing mixed foliar litter on the release of several metallic elements in a Pinus massoniana Lamb. forest. Annals of Forest Science. 77(2), 1-12.

588 S. E. Hobbie, J. Oleksyn, D. M. Eissenstat, P. B. Reich. (2010). Fine root decomposition rates do not mirror those of leaf litter among temperate tree species. Oecologia. 162(2), 505-513.

589 H. Yang, Y. Li, Z. Ning, Z. Zhang. (2019). Home-filed effects of leaf litter decomposition of dominate sand-fixing shrubs in the Horqin Sandy Land. Journal of Desert Research. 39(5), 62-70. (in Chinese with English abstract)

590 B. Hoorens, D. Coomes, R. Aerts. (2010). Neighbour identity hardly affects litter-mixture effects on decomposition rates of New Zealand forest species. Oecologia. 162(2), 479-489.

591 S.-L. Hou, X.-T. Lü. (2021). Mixing effects of litter decomposition at plant organ and species levels in a temperate grassland. Plant and Soil. 459(1), 387-396.

592 J.-H. Huang, X.-G. Han, L.-Z. Chen. (1998). Studies on litter decomposition processes in a temperate forest ecosystem. I. Change of organic matter in oak (Quercus liaotungensis Koidz.) twigs. Ecological Research. 13(2), 163-170.

593 G. Huang, H.-m. Zhao, Y. Li. (2017). Litter decomposition in hyper-arid deserts: Photodegradation is still important. Science of the Total Environment. 601, 784-792.

594 A. Ibrahima, D. Adda Magouo, O. Ibrahim, B. Hassana. (2017). Synergistic effect of earthworms and soil micro-organisms on litter decomposition in Sudano-Guinea savannah zone of Ngaoundere, Cameroun. International Journal of Agricultural Research, Sustainability, and Food Sufficiency. 4(1), 133-150.

595 Y. Xiang, S. An, M. Cheng, L. Liu, Y. Xie. (2018). Changes of soil microbiological properties during grass litter decomposition in loess hilly region, China. International Journal of Environmental Research and Public Health. 15(9), 1797.

596 M. Soni, N. Yadava, S. Bhardwaj. (2016). Dynamics of leaf litter decomposition of four tree species of arid western Rajasthan under varying soil moisture regimes. National Academy of Agricultural Science. 34(4), 955-960.

597 W. Lu, N. Liu, Y. Zhang, J. Zhou, Y. Guo, X. Yang. (2017). Impact of vegetation community on litter decomposition: evidence from a reciprocal transplant study with 13C labeled plant litter. Soil Biology and Biochemistry. 112, 248-257.

598 C.-y. Wang, Y.-n. Lü, L. Wang, X.-y. Liu, X.-j. Tian. (2013). Insights into seasonal variation of litter decomposition and related soil degradative enzyme activities in subtropical forest in China. Journal of Forestry Research. 24(4), 683-689.

599 E. Birhane, T. Desalegn, F. Kebede, K. Giday, H. Hishe, K. M. Hadgu. (2019). In situ leaf litter production, decomposition and nutrient release of dry Afromontane trees. East African Agricultural and Forestry Journal. 83(3), 176-190.

600 T. Sariyildiz, J. Anderson. (2003). Interactions between litter quality, decomposition and soil fertility: a laboratory study. Soil Biology and Biochemistry. 35(3), 391-399.

601 H. Ishikawa, T. Osono, H. Takeda. (2007). Effects of clear-cutting on decomposition processes in leaf litter and the nitrogen and lignin dynamics in a temperate secondary forest. Journal of Forest Research. 12(4), 247-254.

602 J. I. MUOGHALU, O. M. ADELEYE, R. T. BALOGUN. (1994). Litter decomposition and inorganic element dynamics in a secondary rainforest at Ile‐Ife, Nigeria. African Journal of Ecology. 32(3), 208-221.

603 M. S. Strickland, E. Osburn, C. Lauber, N. Fierer, M. A. Bradford. (2009). Litter quality is in the eye of the beholder: initial decomposition rates as a function of inoculum characteristics. Functional Ecology. 23(3), 627-636.

604 A. T. Austin, V. A. Marchesini. (2012). Gregarious flowering and death of understorey bamboo slow litter decomposition and nitrogen turnover in a southern temperate forest in Patagonia, Argentina. Functional Ecology. 26(1), 265-273.

605 A. C. Finzi, W. H. Schlesinger. (2002). Species control variation in litter decomposition in a pine forest exposed to elevated CO2. Global Change Biology. 8(12), 1217-1229.

606 L. Vivanco, A. T. Austin. (2008). Tree species identity alters forest litter decomposition through long‐term plant and soil interactions in Patagonia, Argentina. Journal of Ecology. 96(4), 727-736.

607 S. Hättenschwiler, H. B. Jørgensen. (2010). Carbon quality rather than stoichiometry controls litter decomposition in a tropical rain forest. Journal of Ecology. 98(4), 754-763.

608 B. Hoorens, R. Aerts, M. Stroetenga. (2004). Elevated UV‐B radiation has no effect on litter quality and decomposition of two dune grassland species: evidence from a long‐term field experiment. Global Change Biology. 10(2), 200-208.

609 S. M. Uselman, K. A. Snyder, R. R. Blank. (2011). Insect biological control accelerates leaf litter decomposition and alters short‐term nutrient dynamics in a Tamarix‐invaded riparian ecosystem. Oikos. 120(3), 409-417.

610 Q. Wang, J.-H. Kwak, W.-J. Choi, S. X. Chang. (2018). Decomposition of trembling aspen leaf litter under long-term nitrogen and sulfur deposition: effects of litter chemistry and forest floor microbial properties. Forest Ecology and Management. 412, 53-61.

611 T. Sariyildiz, J. Anderson, M. Kucuk. (2005). Effects of tree species and topography on soil chemistry, litter quality, and decomposition in Northeast Turkey. Soil Biology and Biochemistry. 37(9), 1695-1706.

612 C. Steffens, M. Helfrich, R. G. Joergensen, V. Eissfeller, H. Flessa. (2015). Translocation of 13C-labeled leaf or root litter carbon of beech (Fagus sylvatica L.) and ash (Fraxinus excelsior L.) during decomposition–A laboratory incubation experiment. Soil Biology and Biochemistry. 83, 125-137.

613 R. L. Melliger, H.-P. Rusterholz, B. Baur. (2017). Ecosystem functioning in cities: Combined effects of urbanisation and forest size on early-stage leaf litter decomposition of European beech (Fagus sylvatica L.). Urban Forestry & Urban Greening. 28, 88-96.

614 M. Manna, S. Jha, P. Ghosh, T. Ganguly, K. Singh. (2004). Litter Decomposition in Three Plantation Species in Semi-Arid and Sub-Humid Regions of Central India: Impact of Climate, Substrate Quality and Earthworm Activity. Journal of Sustainable Forestry. 18(1), 47-62.

615 J. Jabiol, E. Chauvet. (2015). Biodiversity and litter decomposition: a case study in a Mediterranean stream. Freshwater Science. 34(2), 423-430.

616 D. Janušauskaitè, V. Baliuckas, Z. Dabkevičius. (2013). Needle litter decomposition of native Pinus sylvestris L. and alien Pinus mugo at different ages affecting enzyme activities and soil properties on dune sands. Baltic Forestry. 19(1), 50-60.

617 P. Kemp, J. Reynolds, R. Virginia, W. Whitford. (2003). Decomposition of leaf and root litter of Chihuahuan desert shrubs: effects of three years of summer drought. Journal of Arid Environments. 53(1), 21-39.

618 Y. Ji, Q. Li, K. Tian, J. Yang, H. Hu, L. Yuan, W. Lu, B. Yao, X. Tian. (2020). Effect of sodium amendments on the home-field advantage of litter decomposition in a subtropical forest of China. Forest Ecology and Management. 468, 118148.

619 X. Jia, Z. He, M. D. Weiser, T. Yin, S. Akbar, X. Kong, K. Tian, Y. Jia, H. Lin, M. Yu. (2016). Indoor evidence for the contribution of soil microbes and corresponding environments to the decomposition of Pinus massoniana and Castanopsis sclerophylla litter from Thousand Island Lake. European Journal of Soil Biology. 77, 44-52.

620 Y. Jia, D. Gu, C. Wu, W. Yang, X. Du, T. Wen, Y. Lv, X. Kong, X. Tian. (2019). Nitrogen deposition slows down the litter decomposition induced by soil macrofauna in the soil of subtropical forests in China. Ecological Research. 34(3), 360-369.

621 X. Jiang, L. Cao, R. Zhang, L. Yan, Y. Mao, Y. Yang. (2014). Effects of nitrogen addition and litter properties on litter decomposition and enzyme activities of individual fungi. Applied Soil Ecology. 80, 108-115.

622 H. Jing, G. Wang. (2020). Temporal dynamics of Pinus tabulaeformis litter decomposition under nitrogen addition on the loess plateau of China. Forest Ecology and Management. 476, 118465.

623 W. Zech, M. B. Johansson, L. Haumaier, R. L. Malcolm. (1987). CPMAS 13C NMR and IR spectra of spruce and pine litter and of the Klason lignin fraction at different stages of decomposition. Zeitschrift für Pflanzenernährung und Bodenkunde. 150(4), 262-265.

624 R. Keenan, C. Prescott, J. Kimmins, J. Pastor, B. Dewey. (1996). Litter decomposition in western red cedar and western hemlock forests on northern Vancouver Island, British Columbia. Canadian Journal of Botany. 74(10), 1626-1634.

625 P. R. Kemp, D. G. Waldecker, C. E. Owensby, J. F. Reynolds, R. A. Virginia. (1994). Effects of elevated CO2 and nitrogen fertilization pretreatments on decomposition on tallgrass prairie leaf litter. Plant and Soil. 165(1), 115-127.

626 I. Laishram, P. Yadava. (1988). Lignin and nitrogen in the decomposition of leaf litter in a sub-tropical forest ecosystem at Shiroy hills in north-eastern India. Plant and Soil. 106(1), 59-64.

627 A. Arunachalam, K. Maithani, H. Pandey, R. Tripathi. (1998). Leaf litter decomposition and nutrient mineralization patterns in regrowing stands of a humid subtropical forest after tree cutting. Forest Ecology and Management. 109(1-3), 151-161.

628 A. Cuchietti, E. Marcotti, D. E. Gurvich, A. M. Cingolani, N. P. Harguindeguy. (2014). Leaf litter mixtures and neighbour effects: low-nitrogen and high-lignin species increase decomposition rate of high-nitrogen and low-lignin neighbours. Applied Soil Ecology. 82, 44-51.

629 S. Tripathi, A. Sumida, H. Shibata, K. Ono, S. Uemura, Y. Kodama, T. Hara. (2006). Leaf litterfall and decomposition of different above-and belowground parts of birch (Betula ermanii) trees and dwarf bamboo (Sasa kurilensis) shrubs in a young secondary forest in Northern Japan. Biology and Fertility of Soils. 43(2), 237-246.

630 X. Zhu, H. Chen, W. Zhang, J. Huang, S. Fu, Z. Liu, J. Mo. (2016). Effects of nitrogen addition on litter decomposition and nutrient release in two tropical plantations with N2-fixing vs. non-N2-fixing tree species. Plant and Soil. 399(1), 61-74.

631 W. Li, W. Yu, L. Bai, H. Liu, D. Yang. (2016). Effects of nitrogen addition on the mixed litter decomposition in Stipa baicalensis steppe in Inner Mongolia. American Journal of Plant Sciences. 7(03), 547.

632 Y. Li, Z. Ning, D. Cui, W. Mao, J. Bi, X. Zhao. (2016). Litter decomposition in a semiarid dune grassland: neutral effect of water supply and inhibitory effect of nitrogen addition. PLoS One. 11(9), e0162663.

633 R. Li, Y. Zhang, D. Yu, Y. Wang, X. Zhao, R. Zhang, W. Zhang, Q. Wang, M. Xu, L. Chen. (2021). The decomposition of green leaf litter is less temperature sensitive than that of senescent leaf litter: An incubation study. Geoderma. 381, 114691.

634 G.-G. Lin, R. Mao, L. Zhao, D.-H. Zeng. (2013). Litter decomposition of a pine plantation is affected by species evenness and soil nitrogen availability. Plant and Soil. 373(1), 649-657.

635 H. A. Bahamonde, P. L. Peri, R. Alvarez, A. Barneix, A. Moretto, G. Martínez Pastur. (2012). Litter decomposition and nutrients dynamics in Nothofagus antarctica forests under silvopastoral use in Southern Patagonia. Agroforestry Systems. 84(3), 345-360.

636 A. Bruder, M. H. Schindler, M. S. Moretti, M. O. Gessner. (2014). Litter decomposition in a temperate and a tropical stream: the effects of species mixing, litter quality and shredders. Freshwater Biology. 59(3), 438-449.

637 J. Chandrashekar. (2011). Litter decomposition of mixed community forest tree species of Garhwal Himalaya, India. Journal of Ecology and Environmental Sciences. 2(1),

638 K. Tian, X. Kong, J. Gao, Y. Jia, H. Lin, Z. He, Y. Ji, Z. Bei, X. Tian. (2018). Local root status: a neglected bio-factor that regulates the home-field advantage of leaf litter decomposition. Plant and Soil. 431(1), 175-189.

639 B. Mao, R. Mao, Y. Hu, Y. Huang, D. Zeng. (2016). Decomposition of Mongolian pine litter in the presence of understory species in semi-arid northeast China. Journal of Forestry Research. 27(2), 329-337.

640 A. V. Marano, M. C. N. Saparrat, M. M. Steciow, M. N. Cabello, F. Gleason, C. Pires-Zottarelli, J. I. de Souza, M. D. Barrera. (2013). Comparative analysis of leaf-litter decomposition from the native Pouteria salicifolia and the exotic invasive Ligustrum lucidum in a lowland stream (Buenos Aires, Argentina). Fundamental and Applied Limnology. 183(4), 297-307.

641 O. A. Marinho, L. A. Martinelli, P. J. Duarte-Neto, E. A. Mazzi, J. Y. King. (2020). Photodegradation influences litter decomposition rate in a humid tropical ecosystem, Brazil. Science of the Total Environment. 715, 136601.

642 C. A. S. Torres. (2015). Microbial decomposers diversity and litter decomposition along an altitudinal gradient in tropical and temperate stream ecosystems.

643 Y. Meng, D. Hui, C. Huangfu. (2020). Site conditions interact with litter quality to affect home-field advantage and rhizosphere effect of litter decomposition in a subtropical wetland ecosystem. Science of the Total Environment. 749, 141442.

644 P. Liu, O. J. Sun, J. Huang, L. Li, X. Han. (2007). Nonadditive effects of litter mixtures on decomposition and correlation with initial litter N and P concentrations in grassland plant species of northern China. Biology and Fertility of Soils. 44(1), 211-216.

645 F. Montané, J. Romanyà, P. Rovira, P. Casals. (2013). Mixtures with grass litter may hasten shrub litter decomposition after shrub encroachment into mountain grasslands. Plant and Soil. 368(1), 459-469.

646 T. Moore. (1984). Litter decomposition in a subarctic spruce‐lichen woodland, Eastern Canada. Ecology. 65(1), 299-308.

647 Y. Lu, K. Li, R. Ni, R. Han, C. Li, C. Zhang. (2020). Leaf and root litter species identity influences bacterial community composition in short-term litter decomposition.

648 R. Nakamura, J.-T. Cornelis, F. de Tombeur, A. Yoshinaga, M. Nakagawa, K. Kitajima. (2020). Diversity of silicon release rates among tropical tree species during leaf-litter decomposition. Geoderma. 368, 114288.

649 S. Nikula, E. Vapaavuori, S. Manninen. (2010). Urbanization-related changes in European aspen (Populus tremula L.): leaf traits and litter decomposition. Environmental Pollution. 158(6), 2132-2142.

650 C. A. Pérez, M. R. Carmona, J. J. Armesto. (2010). Non‐symbiotic nitrogen fixation during leaf litter decomposition in an old‐growth temperate rain forest of Chiloé Island, southern Chile: Effects of single versus mixed species litter. Austral Ecology. 35(2), 148-156.

651 M. L. Silveira, K. R. Reddy, N. B. Comerford. (2011). Litter decomposition and soluble carbon, nitrogen, and phosphorus release in a forest ecosystem. Open Journal of soil science. 1(3), 86-96.

652 Q. Wu. (2020). Short-and long-term effects of snow-depth on Korean Pine and Mongolian Oak litter decomposition in Northeastern China. Ecosystems. 23(3), 662-674.

653 D. Wu, T. Li, S. Wan. (2013). Time and litter species composition affect litter-mixing effects on decomposition rates. Plant and Soil. 371(1), 355-366.

654 D. L. White, B. L. Haines, L. R. Boring. (1988). Litter decomposition in southern Appalachian black locust and pine–hardwood stands: litter quality and nitrogen dynamics. Canadian Journal of Forest Research. 18(1), 54-63.

655 J. Fyles, I. Fyles. (1993). Interaction of Douglas-fir with red alder and salal foliage litter during decomposition. Canadian Journal of Forest Research. 23(3), 358-361.

656 X. Xu, L. Yin, C. Duan, Y. Jing. (2016). Effect of N addition, moisture, and temperature on soil microbial respiration and microbial biomass in forest soil at different stages of litter decomposition. Journal of Soils and Sediments. 16(5), 1421-1439.

657 X. Yang, Y.-b. Qu, N. Yang, H. Zhao, J.-l. Wang, N.-x. Zhao, Y.-b. Gao. (2019). Litter species diversity is more important than genotypic diversity of dominant grass species Stipa grandis in influencing litter decomposition in a bare field. Science of the Total Environment. 666, 490-498.

658 P. Zhang, X. Tian, X. He, F. Song, L. Ren, P. Jiang. (2008). Effect of litter quality on its decomposition in broadleaf and coniferous forest. European Journal of Soil Biology. 44(4), 392-399.

659 J. Zhang, D. Zhang, Z. Jian, H. Zhou, Y. Zhao, D. Wei. (2019). Litter decomposition and the degradation of recalcitrant components in Pinus massoniana plantations with various canopy densities. Journal of Forestry Research. 30(4), 1395-1405.

660 J. Zhang, J. Li, Y. Fan, Q. Mo, Y. Li, Y. Li, Z. Li, F. Wang. (2020). Effect of nitrogen and phosphorus addition on litter decomposition and nutrients release in a tropical forest. Plant and Soil. 454(1), 139-153.

661 X. Zhang, W. Jiang, S. Jiang, W. Tan, R. Mao. (2021). Differential responses of litter decomposition in the air and on the soil surface to shrub encroachment in a graminoid-dominated temperate wetland. Plant and Soil. 462(1), 477-488.

662 Q. Zhang, G. Zhang, X. Yu, Y. Liu, S. Xia, Z. Meng, C. Xu. (2021). How do droppings of wintering waterbird accelerate decomposition of Carex cinerascens Kükenth litter in seasonal floodplain Ramsar Site? Wetlands Ecology and Management. 29(4), 581-597.

663 H. Zhao, G. Huang, J. Ma, Y. Li, L. Tang. (2014). Decomposition of aboveground and root litter for three desert herbs: mass loss and dynamics of mineral nutrients. Biology and Fertility of Soils. 50(5), 745-753.

664 H. Zheng, Y. Chen, Y. Liu, P. Heděnec, Y. Peng, Z. Xu, B. Tan, L. Zhang, L. Guo, L. Wang. (2021). Effects of litter quality diminish and effects of vegetation type develop during litter decomposition of two shrub species in an alpine treeline ecotone. Ecosystems. 24(1), 197-210.

665 W. He, Z. Ma, J. Pei, M. Teng, L. Zeng, Z. Yan, Z. Huang, Z. Zhou, P. Wang, X. Luo. (2019). Effects of predominant tree species mixing on lignin and cellulose degradation during leaf litter decomposition in the Three Gorges Reservoir, China. Forests. 10(4), 360.

666 P. I. Araujo, A. T. Austin. (2015). A shady business: pine afforestation alters the primary controls on litter decomposition along a precipitation gradient in Patagonia, Argentina. Journal of Ecology. 103(6), 1408-1420.

667 R. L. Edmonds. (1987). Decomposition rates and nutrient dynamics in small-diameter woody litter in four forest ecosystems in Washington, USA. Canadian Journal of Forest Research. 17(6), 499-509.

668 F. Trum, H. Titeux, Q. Ponette, B. Berg. (2015). Influence of manganese on decomposition of common beech (Fagus sylvatica L.) leaf litter during field incubation. Biogeochemistry. 125(3), 349-358.

669 X. Ge, W. Xiao, L. Zeng, Z. Huang, T. Fu, X. Feng. (2012). Relationships between litter substrate quality and soil nutrients in different-aged Pinus massoniana stands. Acta Ecologica Sinica. 32(3), 852-862. (in Chinese with English abstract)

670 L. Wu, A. Zhang, L. Mou, Y. Li, H. Li, C. You, L. Zhang, B. Tan, Z. Xu. (2021). Litter decomposition of seven common colored-leaf tree species of western Sichuan. Chinese Journal of Applied & Environmental Biology. 27(3), 625-631. (in Chinese with English abstract)

671 Y. S. Yang, Y. Chen, Z. He, J. F. Guo. (2004). Comparatively Study on Litter Properties Between Plantations of Fokienia hodginsii and Cunninghamia lanceolata. Scientia Silvae Sinicae. 40(1), 2-10. (in Chinese with English abstract)

672 Z. Zeng, K. Wang, F. Zeng, T. Song, X. Liu, X. SOng. (2012). Litter decomposition and nutrient release in typical secondary and primary forests in karst region，Northwest of Guangxi. Acta Ecologica Sinica. 32(9), 2720-2728. (in Chinese with English abstract)

673 X. Li, Y. Liu, J. Zhang, W. Yang, Y. Zhang, C. Deng, M. Zhang, H. Liu. (2016). Effect of forest gap size on loss of dissolved organic carbon ( DOC) for four leaf litters. Journal of Northeast Forestry University. 44(1), 23-30. (in Chinese with English abstract)

674 R. Dou, H. Jiang, S. Yu, Y. Ma, P. Guo. (2010). Decomposition of Cryptomeria fortunei leaf litter in subtropical and tropical China. Acta Ecologica Sinica. 30(7), 1758-1763. (in Chinese with English abstract)

675 X. Li, Y. Liu, Y. Zhang, H. Liu, W. Yang, J. Zhang. (2016). Effects of soil fauna on C dynamics in different sized forest gaps in Pinus massoniana plantation. Bulletin of Botanical Research. 36(2), 195-203. (in Chinese with English abstract)

676 Y. Qin, D. Zhang, X. Li, Y. Zhang, Y. Yuan, L. Wang, Z. Pang, J. Zhang. (2018). Changes of total phenols and condensed tannins during the decomposition of mixed leaf litter of Pinus massoniana and broad-leaved trees. Chinese Journal of Applied Ecology. 29(7), 2224-2232. (in Chinese with English abstract)

677 Z. Liu, S. Wang, J. Han, Z. Chen. (2005). Decomposition and nutrients dynamics of plant litter and roots in Inner Mongolia steppe. Acta Prataculturae Sinica. 14(1), 24-30. (in Chinese with English abstract)

678 N. Lin. (2011). A study on decomposition characteristics of needle litter from Pinus tabulaeformis artificial plantations under different densities. Beijing Forestry University. (in Chinese with English abstract)

679 J. Wang. (2013). Study on decomposition of leaf litters from two dominant species under different geological background. Guangxi Normal University. (in Chinese with English abstract)

680 B. Lei. (2013). Study on the soil organic matter decomposition accumulation and storage prediction of Subalpine Abies faxoniana Forest in western Sichuan. Sichuan Agricultural University. (in Chinese with English abstract)

681 Y. Jia. (2015). Effects of nitrogen and sodium addition on soil fauna-litter decomposition system in subtropical forests. Nanjing University. (in Chinese with English abstract)

682 D. Liang. (2016). The effects of litter quality, soil fauna and dung addition on litter decomposition in a Tibetan alpine meadow. Lanzhou University. (in Chinese with English abstract)

683 C. Yang. (2014). Decomposition characteristics in decomposition process of Pinus armandii coniferous litter at Erlangshan Mount, and indoor decomposition experiment. Sichuan Agricultural University. (in Chinese with English abstract)

684 D. Liang. (2013). The additive and non-additive effects of the mixed litter decomposition and N, P releae in an apline meadow. Lanzhou University. (in Chinese with English abstract)

685 J. Zhu. (2011). Litter decomposition in response to seasonal freezing and thawing in the alpine/subalpine forest. Sichuan Agricultural University. (in Chinese with English abstract)

686 L. Jin. (2015). Research on litter decomposition characteristics of rubber plantation in Danzhou, Hainan. Hainan University. (in Chinese with English abstract)

687 Z. Wei. (2017). Responses of decomposition of Flaveria bidentis litters to simulated nitrogen deposition. Shenyang Agricultural University. (in Chinese with English abstract)

688 Y. Liang. (2016). Effects of simulated nitrogen deposition on belowground carbon cycling processes and soil biochemical characteristics in a secondary evergreen broad-leaved forest on Wawu Mountain. Sichuan Agricultural University. (in Chinese with English abstract)

689 J. Hong. (2011). Effect of simulated acid rain and nitrogen deposition respectively on the leaf litter decomposition of three dominant trees in the subtropical forests. Southwest University. (in Chinese with English abstract)

690 X. Ge. (2012). The influence of litter decomposition on soil carbon pools dynamics of Pinus massoniana stands in three gorges reservoir area. Chinese Academy of Forestry. (in Chinese with English abstract)

691 X. Zhang. (2017). Litter decomposition characteristics of main species in Tarim Desert Highway Shelterbelt. Xinjiang University. (in Chinese with English abstract)

692 H. Zhang, X. Song, Z. Zhang, H. Jiang, Y. Wang, S. Bai. (2011). Effects of UV-B radiation on the decomposition of Cunninghamia lanceolata leaf litter. Chinese Journal of Applied Ecology. 22(4), 845-850. (in Chinese with English abstract)

693 X. Song, H. Zhang, H. Jiang, S. Yu, Z. Zhang. (2011). Effect of UV-B radiation on the leaf litter decomposition and nutrient release of Pinus massoniana. Acta Botanica Brasilica. 31(8), 2106-2114.

694 C. Wang, W. Cui, P. Zhao, D. Xiao, X. Wang. (2021). Decomposition characteristics of common wetland emergent plants in Dianchi Lake. Guihaia. 1-19. (in Chinese with English abstract)

695 X. Wang, Y. Yang, N. Yang, X. Xin, Y. Qu, N. Zhao, Y.-b. Gao. (2019). Effects of litter diversity and composition on litter decomposition characteristics and soil microbial community. Acta Ecologica Sinica. 39(17), 6264-6272. (in Chinese with English abstract)

696 M. Tu, W. Yao, H. Weng, Z. Li. (1993). Characteristics of litter in evergreen broadleaved forest of the Dinghu Mountain. Acta Pedologica Sinica. 30(1), 34-42. (in Chinese with English abstract)

697 L. Chao, Z. Li, D. Yang, A. Wang, J. Zhang, B. Hu, Y. Liu. (2021). Comparison of chemical traits between drought-dead and natural litter leaves. Journal of Tropical and Subtropical Botany. 1-10. (in Chinese with English abstract)

698 Y. S. Yang, P. Lin, J. Guo, R. Lin, G. Chen, Z. He, J. Xin. (2003). Litter production, nutrient return and leaf-litter decomposition in natural and monoculture plantation forests of Castanopsis kawakamii in subtropical China. Acta Ecologica Sinica. 23(7), 1278-1289. (in Chinese with English abstract)

699 X. Yue, G. Ye, W. Gao, Z. Chen, M. Cheng, D. Li. (2021). Characteristics of litter decomposition and ecological stoichiometry of different forests on Coastal Sandy Land in Fujian Province. Research of Soil and Water Conservation. 28(4), 77-83. (in Chinese with English abstract)

700 Y. Peng, W. Yang, Q. Xue, J. Li, B. Wang, B. Tan, F. Wu. (2016). Impact of soil fauna on lignin degradation of two foliar litters in an alpine meadow during freezing-thawing period. Chinese Journal of Applied & Environmental Biology. 22(2), 300-306. (in Chinese with English abstract)

701 X. Li, N. Cui, J. Zhang, Y. Liu, Y. Zhang, C. Deng, M. Zhang, H. Liu. (2016). Mass loss of Pinus massoniana and Cinnamomum camphora leaf litter in forest gaps of different size. Chinese Journal of Applied & Environmental Biology. 22(2), 292-299. (in Chinese with English abstract)

702 H. Song, H. Hong, S. Chen, C. Lin, Y. Yang. (2021). Effects of phosphorus addition on fine root decomposition of Castanopsis carlesii, Cunninghamia lanceolata and mixed. Journal of Forest and Environment. 41(1), 1-9. (in Chinese with English abstract)

703 Y. Zhang, X. Li, H. Liu, M. Zhang, W. Yang, J. Zhang. (2016). Edge effects of forest gap in Pinus massoniana plantations on the decomposition of leaf litter recalcitrant components of Cinnamomum camphora and Toona ciliata. Chinese Journal of Applied Ecology. 27(4), 1116-1124. (in Chinese with English abstract)

704 H. Liu, D. Zhang, J. Zhang, W. Yang, X. Li, Y. Zhang, M. Zhang. (2017). Effects of forest gap size on leaf litter weight loss and nutrient release of four species in Pinus massoniana plantations. Acta Ecologica Sinica. 37(2), 513-522. (in Chinese with English abstract)

705 X. Li, Y. Liu, J. Zhang, Y. Zhang, M. ZHang. (2015). Seasonal variations on carbon release of three foliar litter in forest gap of Pinus massoniana plantations. Ecology and Environmental Sciences. 24(10), 1634-1639. (in Chinese with English abstract)

706 X. Ge, L. Zeng, W. Xiao, Z. Huang, B. Zhou. (2017). Relationship between leaf litter decomposition and soil C，N stoichiometry in different-aged Pinus massioniana stands exposed to simulated nitrogen deposition. Acta Ecologica Sinica. 37(4), 1147-1158. (in Chinese with English abstract)

707 M. Jiao, W. Shen. (2014). Effects of seasonal precipitation variation on litter-fall in lower subtropical evergreen broad-leaved forest. Journal of Tropical and Subtropical Botany. 22(6), 549-557. (in Chinese with English abstract)

708 X. Li, X. Wan, F. Zhou, B. Zou, S. Wang. (2020). Characteristics of soil microbial biomass and community structure under six different tree species plantations in southern Subtropical of China. Journal of Subtropical Resources and Environment. 15(1), 33-40. (in Chinese with English abstract)

709 N. Bijayalaxmi Devi, P. Yadava. (2010). Influence of climate and litter quality on litter decomposition and nutrient release in sub-tropical forest of Northeast India. Journal of Forestry Research. 21(2), 143-150.

710 C. Zhou, L. Liu, X. Shen, W. Gao, Q. Li, F. Sun, J. Zhao, J. Zhou. (2019). Spatial heterogeneity of the decomposition characteristics of the litter leaf of Salix cupularis in an alpine desertified meadow ecosystem in the eastern Tibetan Plateau. Chinese Journal of Applied & Environmental Biology. 25(4), 808-816. (in Chinese with English abstract)

711 P. Dou, G. Lin, F. Wang, Y. Huang, J. Gao, W. Yang, B. Wei, W. Li, J. Yao. (2021). Litter mixed decomposition effect of urban eutrophic streams in the Three Gorges Reservoir area. China Environmental Science. 41(8), 3775-3783. (in Chinese with English abstract)

712 Y. Lu, K. Li, R. Ni, Q. Liang, C. Li, C. Zhang. (2018). Effects of fine root decomposition on bacterial community structure of four dominated tree species in Mount Taishan, China. Chinese Journal of Plant Ecology. 42(12), 1200-1210. (in Chinese with English abstract)

713 R. Feng, Y. Liu, K. Guo, L. Lan, Y. Chen, J. Liu, B. Tan. (2020). Effects of soil arthropod on enzyme activities during leaf litter decomposition of bamboo (Fargesia spathacea). Chinese Journal of Ecology. 39(3), 775-785. (in Chinese with English abstract)

714 W. He, F. Wu, W. Yang, q. Wu, M. He, Y. Zhao. (2013). Effect of snow patches on leaf litter mass loss of two shrubs in an alpine forest Chinese Journal of Plant Ecology 37(4), 306-316. (in Chinese with English abstract)

715 J. Yang, X. Wan. (2020). Effects of the calcium of leaf litters of different afforestation species on soil characteristics in subtropical zone. Fujian Agricultural Science and Technology. (1), 24-32. (in Chinese with English abstract)

716 Z. Ma, S. Gao, W. Yang, F. Wu. (2015). Degradation characteristics of lignin and cellulose of foliar litter at different rainy stages in subtropical evergreen broadleaved forest. Chinese Journal of Ecology. 34(1), 122-129. (in Chinese with English abstract)

717 F. Montané, J. Romanyà, P. Rovira, P. Casals. (2010). Aboveground litter quality changes may drive soil organic carbon increase after shrub encroachment into mountain grasslands. Plant and Soil. 337(1), 151-165.

718 E. C. Adair, W. J. Parton, J. Y. King, L. A. Brandt, Y. Lin. (2017). Accounting for photodegradation dramatically improves prediction of carbon losses in dryland systems. Ecosphere. 8(7), e01892.

719 Y. Xie, H. Qin, D. Yu. (2004). Nutrient limitation to the decomposition of water hyacinth (Eichhornia crassipes). Hydrobiologia. 529(1), 105-112.

720 N. W. Mungai, P. P. Motavalli. (2006). Litter quality effects on soil carbon and nitrogen dynamics in temperate alley cropping systems. Applied Soil Ecology. 31(1-2), 32-42.

721 A. Poi de Neiff, J. J. Neiff, S. L. Casco. (2006). Leaf litter decomposition in three wetland types of the Paraná River floodplain. Wetlands. 26(2), 558-566.

722 F. Hagedorn, M. Machwitz. (2007). Controls on dissolved organic matter leaching from forest litter grown under elevated atmospheric CO2. Soil Biology and Biochemistry. 39(7), 1759-1769.

723 K. Wickings, A. S. Grandy, S. C. Reed, C. C. Cleveland. (2012). The origin of litter chemical complexity during decomposition. Ecology Letters. 15(10), 1180-1188.

724 A. B. Stoler, R. A. Relyea. (2013). Leaf litter quality induces morphological and developmental changes in larval amphibians. Ecology. 94(7), 1594-1603.

725 V. Upadhyay. (1990). Decomposition of oak leaf litter in a pine-mixed broadleaf forest in Himalaya. Indian Journal of Ecology. 17(1), 25-28.

726 V. Upadhyay. (1993). Effect of initial litter quality on decomposition rates of the tree leaf litter in Himalayan forest ecosystems. Tropical Ecology.

727 R. Ostertag, S. E. Hobbie. (1999). Early stages of root and leaf decomposition in Hawaiian forests: effects of nutrient availability. Oecologia. 121(4), 564-573.

728 M. R. Shaw, J. Harte. (2001). Control of litter decomposition in a subalpine meadow–sagebrush steppe ecotone under climate change. Ecological Applications. 11(4), 1206-1223.

729 E. F. Salamanca, N. Kaneko, S. Katagiri. (1997). Comparison of field and laboratory microcosm methods on the mass loss of Quercus serrata andPinus densiflora leaf litter. Journal of Forest Research. 2(3), 159-164.

730 T. E. Crews, H. Farrington, P. M. Vitousek. (2000). Changes in asymbiotic, heterotrophic nitrogen fixation on leaf litter of Metrosideros polymorpha with long-term ecosystem development in Hawaii. Ecosystems. 3(4), 386-395.

731 N. A. Scott, S. Saggar, P. D. McIntosh. (2001). Biogeochemical impact of Hieracium invasion in New Zealand's grazed tussock grasslands: sustainability implications. Ecological Applications. 11(5), 1311-1322.

732 E. Dorrepaal, J. H. Cornelissen, R. Aerts, B. Wallen, R. S. Van Logtestijn. (2005). Are growth forms consistent predictors of leaf litter quality and decomposability across peatlands along a latitudinal gradient? Journal of Ecology. 93(4), 817-828.

733 T. Osono, H. Takeda. (2004). Accumulation and release of nitrogen and phosphorus in relation to lignin decomposition in leaf litter of 14 tree species. Ecological Research. 19(6), 593-602.

734 Y. Hu, L. CHen, F. Kong, X. Bao. (1986). The litter decomposition rates of two endemic tree species of China. Acta Phytoecologica ET Geobotanica Sinica. 10(1), 35-43. (in Chinese with English abstract)

735 R. Kaushal, K. Verma, in Trabajo presentado en el XII World Forestry Congress, Quebec, Canada. (2003).

736 J. B. Yavitt, T. J. Fahey. (1986). Litter decay and leaching from the forest floor in Pinus contorta (lodgepole pine) ecosystems. The Journal of Ecology. 74, 525-545.

737 D. A. Wedin, D. Tilman. (1990). Species effects on nitrogen cycling: a test with perennial grasses. Oecologia. 84(4), 433-441.

738 L. Vivanco, A. T. Austin. (2006). Intrinsic effects of species on leaf litter and root decomposition: a comparison of temperate grasses from North and South America. Oecologia. 150(1), 97-107.

739 P. M. Vitousek, S. Hobbie. (2000). Heterotrophic nitrogen fixation in decomposing litter: patterns and regulation. Ecology. 81(9), 2366-2376.

740 P. M. Vitousek. (1998). Foliar and Litter Nutrients, Nutrient Resorption, and Decomposition in Hawaiian Me t rosideros polymorpha. Ecosystems. 1(4), 401-407.

741 N. H. Sullivan, W. B. Bowden, W. H. McDowell. (1999). Short‐Term Disappearance of Foliar Litter in Three Species Before and After a Hurricane. Biotropica. 31(3), 382-393.

742 L. M. Stump, D. Binkley. (1993). Relationships between litter quality and nitrogen availability in Rocky Mountain forests. Canadian Journal of Forest Research. 23(3), 492-502.

743 T. Sariyildiz, J. Anderson. (2005). Variation in the chemical composition of green leaves and leaf litters from three deciduous tree species growing on different soil types. Forest Ecology and Management. 210(1-3), 303-319.

744 H. L. Gholz, C. Perry, W. Cropper Jr, L. Hendry. (1985). Litterfall, decomposition, and nitrogen and phosphorus dynamics in a chronosequence of slash pine (Pinus elliottii) plantations. Forest Science. 31(2), 463-478.

745 T. Sariyildiz. (2003). Litter decomposition of Picea orientalis, Pinus sylvestris and Castanea sativa trees grown in Artvin in relation to their initial litter quality variables(re). Turkish Journal of Agriculture and Forestry. 27(4), 237-243.

746 P. Satti, M. J. Mazzarino, M. Gobbi, F. Funes, L. Roselli, H. Fernandez. (2003). Soil N dynamics in relation to leaf litter quality and soil fertility in north‐western Patagonian forests. Journal of Ecology. 91(2), 173-181.

747 M. Semmartin, M. R. Aguiar, R. A. Distel, A. S. Moretto, C. M. Ghersa. (2004). Litter quality and nutrient cycling affected by grazing‐induced species replacements along a precipitation gradient. Oikos. 107(1), 148-160.

748 J. B. Ferrari. (1999). Fine-scale patterns of leaf litterfall and nitrogen cycling in an old-growth forest. Canadian Journal of Forest Research. 29(3), 291-302.

749 M. R. Downs, K. J. Nadelhoffer, J. M. Melillo, J. D. Aber. (1996). Immobilization of a 15N-labeled nitrate addition by decomposing forest litter. Oecologia. 105(2), 141-150.

750 B. Berg, A. V. De Santo, F. A. Rutigliano, A. Fierro, G. Ekbohm. (2003). Limit values for plant litter decomposing in two contrasting soils—influence of litter elemental composition. Acta Oecologica. 24(5-6), 295-302.

751 G. Hirschel, C. Körner, J. Arnone Iii. (1997). Will rising atmospheric CO2 affect leaf litter quality and in situ decomposition rates in native plant communities? Oecologia. 110(3), 387-392.

752 S. E. Hobbie, P. B. Reich, J. Oleksyn, M. Ogdahl, R. Zytkowiak, C. Hale, P. Karolewski. (2006). Tree species effects on decomposition and forest floor dynamics in a common garden. Ecology. 87(9), 2288-2297.

753 J. M. Lavery, P. G. Comeau, C. E. Prescott. (2004). The influence of red alder patches on light, litterfall, and soil nutrients in adjacent conifer stands. Canadian Journal of Forest Research. 34(1), 56-64.

754 Y. Hu, L. Chen, Q. Chen, F. Kong, Y. Miao. (1987). Study on the litter decomposition rates of several plants. Acta Phytoecologica ET Geobotanica Sinica. 11(2), 124-132. (in Chinese with English abstract)

755 C. A. Lorena, V. D. Noé, C. M. Victoria, B. M. Beatriz, S. C. Leticia, M. M. Julia. (2005). Soil nitrogen in relation to quality and decomposability of plant litter in the Patagonian Monte, Argentina. Plant Ecology. 181(1), 139-151.

756 A. S. Moretto, R. A. Distel. (2002). Soil nitrogen availability under grasses of different palatability in a temperate semi‐arid rangeland of central Argentina. Austral Ecology. 27(5), 509-514.

757 B. Berg, M.-B. Johansson, V. Meentemeyer. (2000). Litter decomposition in a transect of Norway spruce forests: substrate quality and climate control. Canadian Journal of Forest Research. 30(7), 1136-1147.

758 N. Fierer, J. M. Craine, K. McLauchlan, J. P. Schimel. (2005). Litter quality and the temperature sensitivity of decomposition. Ecology. 86(2), 320-326.

759 B. Berg, M.-B. Johansson, G. Ekbohm, C. McClaugherty, F. Rutigliano, A. V. D. Santo. (1996). Maximum decomposition limits of forest litter types: a synthesis. Canadian Journal of Botany. 74(5), 659-672.

760 G. M. Lovett, K. C. Weathers, M. A. Arthur, J. C. Schultz. (2004). Nitrogen cycling in a northern hardwood forest: do species matter? Biogeochemistry. 67(3), 289-308.

761 A. Gallardo, J. Merino. (1992). Nitrogen immobilization in leaf litter at two Mediterranean ecosystems of SW Spain. Biogeochemistry. 15(3), 213-228.

762 A. O'Connell. (1988). Nutrient dynamics in decomposing litter in karri (Eucalyptus diversicolor F. Muell.) forests of south-western Australia. The Journal of Ecology. 76, 1186-1203.

763 R. Aerts, H. de Caluwe. (1997). Nutritional and plant‐mediated controls on leaf litter decomposition of Carex species. Ecology. 78(1), 244-260.

764 M.-B. Johansson. (1994). Decomposition rates of Scots pine needle litter related to site properties, litter quality, and climate. Canadian Journal of Forest Research. 24(9), 1771-1781.

765 Y. Hu, L. CHen, F. Kong, J. Ren. (1986). The study on the litter decomposition of Chinese pine oriental oak. Journal of Integrative Plant Biology. 28(1), 102-110. (in Chinese with English abstract)

766 W. H. Schlesinger, M. M. Hasey. (1981). Decomposition of chaparral shrub foliage: losses of organic and inorganic constituents from deciduous and evergreen leaves. Ecology. 62(3), 762-774.

767 B. Berg, J. E. Lundmark. (1987). Decomposition of needle litter in Pinus contorta and Pinus sylvestris monocultures—a comparison. Scandinavian Journal of Forest Research. 2(1-4), 3-12.

768 V. Upadhyay, J. Singh, V. Meentemeyer. (1989). Dynamics and weight loss of leaf litter in Central Himalayan forests: abiotic versus litter quality influences. The Journal of Ecology. 147-161.

769 A. Gallardo, J. Merino. (1993). Leaf decomposition in two Mediterranean ecosystems of southwest Spain: influence of substrate quality. Ecology. 74(1), 152-161.

770 P. M. Vitousek, D. R. Turner, W. J. Parton, R. L. Sanford. (1994). Litter decomposition on the Mauna Loa environmental matrix, Hawai'i: patterns, mechanisms, and models. Ecology. 75(2), 418-429.

771 H. M. Quested, M. C. Press, T. V. Callaghan. (2003). Litter of the hemiparasite Bartsia alpina enhances plant growth: evidence for a functional role in nutrient cycling. Oecologia. 135(4), 606-614.

772 L. Singh, D. Thakur, M. K. Sharma, A. Chawla. (2021). Dynamics of leaf litter decomposition in the timberline zone of western Himalaya. Acta Oecologica. 111, 103715.

773 H. J. Sarabi, B. Pilehvar, K. A. Vajari, S. M. Waez-Mousavi. (2021). Effects of tree species diversity on leaf litter decomposition process in semi-arid Mediterranean oak forests. European Journal of Forest Research. 140(6), 1377-1390.

774 D. P. Siqueira, G. C. M. W. de Carvalho, J. G. de Souza Silva, M. V. W. Caldeira, D. G. Barroso. (2021). Litter decomposition and nutrient release for two tropical N-fixing species in Rio de Janeiro, Brazil. Journal of Forestry Research. 1-10.

775 R. Pastorelli, V. Costagli, C. Forte, C. Viti, B. Rompato, G. Nannini, G. Certini. (2021). Litter decomposition: Little evidence of the “home-field advantage” in a mountain forest in Italy. Soil Biology and Biochemistry. 159, 108300.

776 F. Cuassolo, V. D. Villanueva, B. Modenutti. (2021). Low-decomposition rates of riparian litter in a North Patagonian ultraoligotrophic lake. Limnologica. 90, 125906.

777 Y. Su, J. Le, X. Ma, X. Zhou, Y. Zhang, Y. Gong, W. Han, K. Li, X. Liu. (2021). Soil burial has a greater effect on litter decomposition rate than nitrogen enrichment in alpine grasslands. Journal of Plant Ecology. 14(6), 1047-1059.

778 Z. Liang, M. Hong, H. De, H. Ye, Y. Zhang, J. Yan, J. Li. (2021). Effects of soil meso- and micro-fauna on litter decomposition under nitrogen deposition and rainfall changes. Chinese Journal of Applied Ecology. 32(12), 4279-4288. (in Chinese with English abstract)

779 S. Liu, R. Yang, C. Hou, J. Ma, J. Guo. (2021). Decomposition characteristics of lignin and cellulose in different litters of ecological tea gardens in mountainous areas of Guizhou. Journal of Tea Science. 41(5), 654-668. (in Chinese with English abstract)

780 X. Zhang, L. Wang, Y. Ji. (2021). Effects of simulated nitrogen deposition on decomposition of foliar litter of Robinia pseudoacacia L. and Hippophae rhamnoides L. Plant Science Journal. 39(4), 398-406. (in Chinese with English abstract)

781 D. A. Wardle, P. J. Bellingham, K. I. Bonner, C. P. Mulder. (2009). Indirect effects of invasive predators on litter decomposition and nutrient resorption on seabird‐dominated islands. Ecology. 90(2), 452-464.

782 B. Duan, J. Lu, M. Liu, M. Yang, Y. Wang, Z. Wang, H. Yang. (2016). Relationships between biological nitrogen fixation and leaf resorption of nitrogen phosphorus, and potassium in the rain-fed region of eastern Gansu, China. Acta Prataculturae Sinica. 25(12), 76-83. (in Chinese with English abstract)

783 P. Diehl, M. J. Mazzarino, S. Fontenla. (2008). Plant limiting nutrients in Andean-Patagonian woody species: effects of interannual rainfall variation, soil fertility and mycorrhizal infection. Forest Ecology and Management. 255(7), 2973-2980.

784 B. Duan, C. Dai, Y. Li, Z. Li, R. Chen, L. Wang, P. Zhou, Q. Li. (2011). Nutritional value of fallen leaves of Avicennia marina and Aegiceras corniculatum at Jiulong River Estuary Fujian Province. Journal of Quanzhou Normal University. 29(2), 28-30. (in Chinese with English abstract)

785 J. Wang. (2013). Study on decomposition of leaf litters from two dominant species under different geological background(re). Guangxi Normal University. (in Chinese with English abstract)

786 G. Li. (2007). Studies on understory vegetation and soil properties affected by tree density in coniferous plantations. Beijing Forestry University. (in Chinese with English abstract)

787 J. Zhao. (2018). the impact of temperature and acidity on decomposition and dissolved organic carbon release from leaf litter in Greater Xingan Mountains. Inner Mongolia Agricultural University. (in Chinese with English abstract)

788 S. Fujii, J. H. Cornelissen, M. P. Berg, A. S. Mori. (2018). Tree leaf and root traits mediate soil faunal contribution to litter decomposition across an elevational gradient. Functional Ecology. 32(3), 840-852.

789 M. D. Norris, P. G. Avis, P. B. Reich, S. E. Hobbie. (2013). Positive feedbacks between decomposition and soil nitrogen availability along fertility gradients. Plant and Soil. 367(1), 347-361.

790 S. Tripathi, K. Singh. (1992). Nutrient immobilization and release patterns during plant decomposition in a dry tropical bamboo savanna, India. Biology and Fertility of Soils. 14(3), 191-199.

791 K. Arunachalam, N. D. Singh, A. Arunachalam. (2003). Decomposition of leguminous crop residues in a ‘jhum’cultivation system in Arunachal Pradesh, India. Journal of Plant Nutrition and Soil Science. 166(6), 731-736.

792 T. L. van Huysen, M. E. Harmon, S. S. Perakis, H. Chen. (2013). Decomposition and nitrogen dynamics of 15N-labeled leaf, root, and twig litter in temperate coniferous forests. Oecologia. 173(4), 1563-1573.

793 J. Bloomfield, K. A. Vogt, D. J. Vogt. (1993). Decay rate and substrate quality of fine roots and foliage of two tropical tree species in the Luquillo Experimental Forest, Puerto Rico. Plant and Soil. 150(2), 233-245.

794 Z. Liu, S. Wang, J. Han, Z. Chen. (2005). Decomposition and nutrients dynamics of plant litter and roots in Inner Mongolia steppe(re). Acta Prataculturae Sinica. 14(1), 24-30. (in Chinese with English abstract)

795 W. Zhu, G. Zhang, P. Zhang, Q. Zhang, J. Ren, B. Xu, H. Qing. (2021). Decomposition characteristics of leaf litters and roots of six main plant species and their relationships with functional traits in Stipa grandis steppe. Chinese Journal of Plant Ecology. 45(6), 606-616. (in Chinese with English abstract)
